# Supplementary figures and images for: Discovery and characterization of a specific inhibitor of serine-threonine kinase cyclin-dependent kinase-like 5 (CDKL5) demonstrates role in hippocampal CA1 physiology (part 1 of 2)
Source: eLife. 2023 Jul 25;12:e88206. doi: 10.7554/eLife.88206 (PMC10406435; doi:10.7554/eLife.88206)

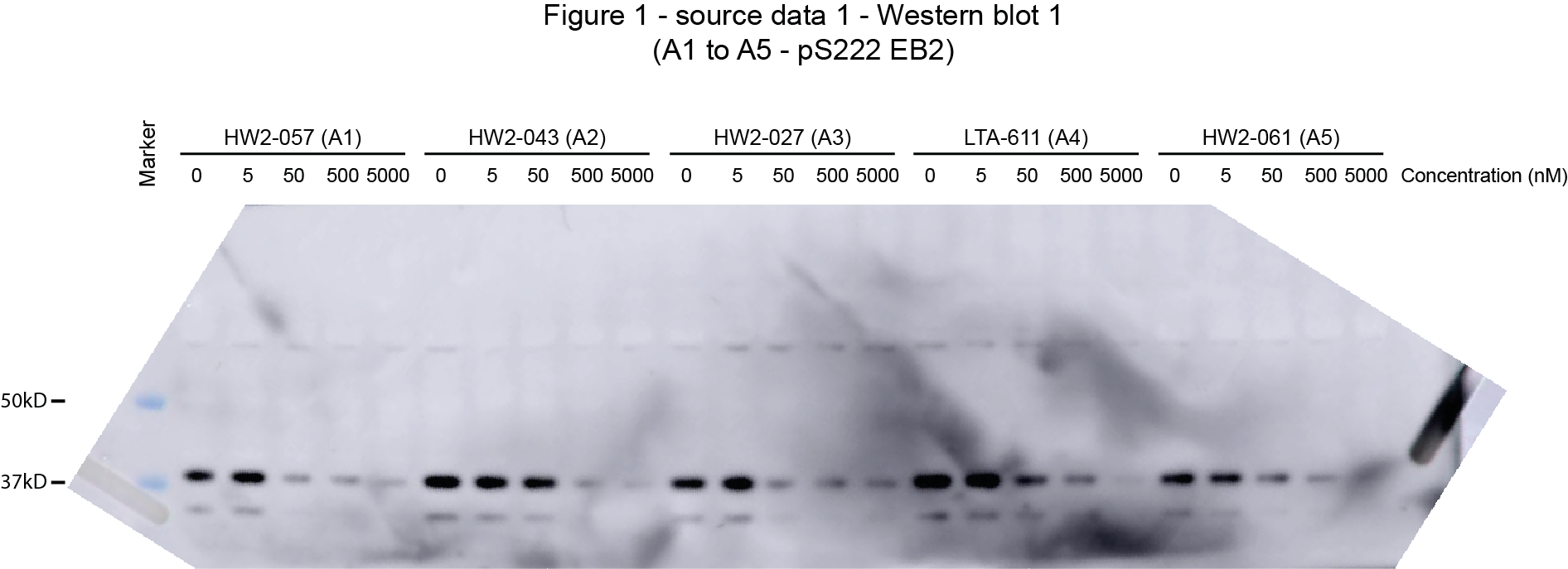

Supplement: Figure 1—source data 1. [file elife-88206-fig1-data1.zip › Figure 1 - source data/Figure 1 - source data 1/Western blot 1 - A1 to A5 - pEB2 - labeled.png]

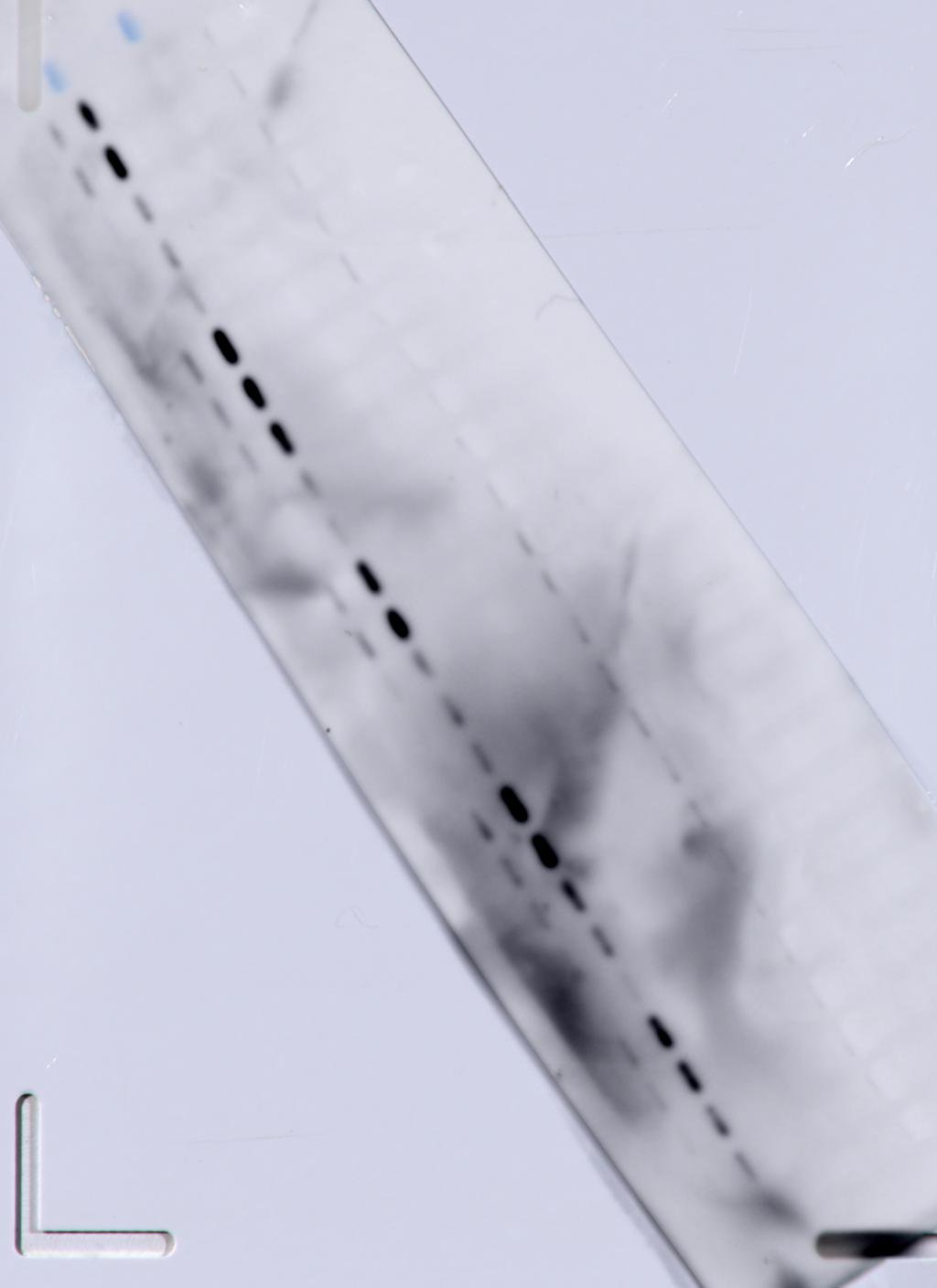

Supplement: Figure 1—source data 1. [file elife-88206-fig1-data1.zip › Figure 1 - source data/Figure 1 - source data 1/Western blot 1 - A1 to A5 - pEB2 - uncropped - marker.jpg]

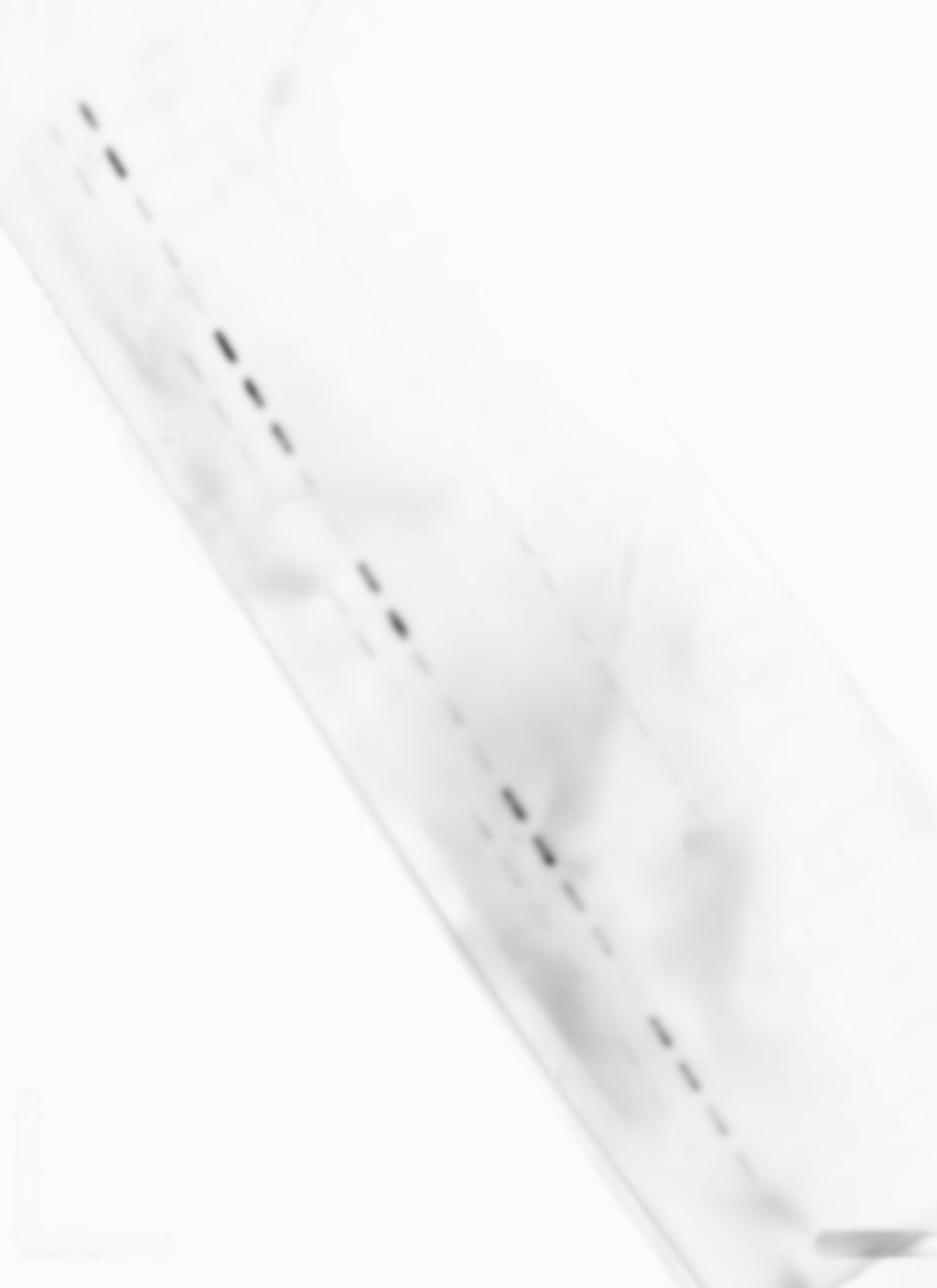

Supplement: Figure 1—source data 1. [file elife-88206-fig1-data1.zip › Figure 1 - source data/Figure 1 - source data 1/Western blot 1 - A1 to A5 - pEB2 - uncropped.tif]

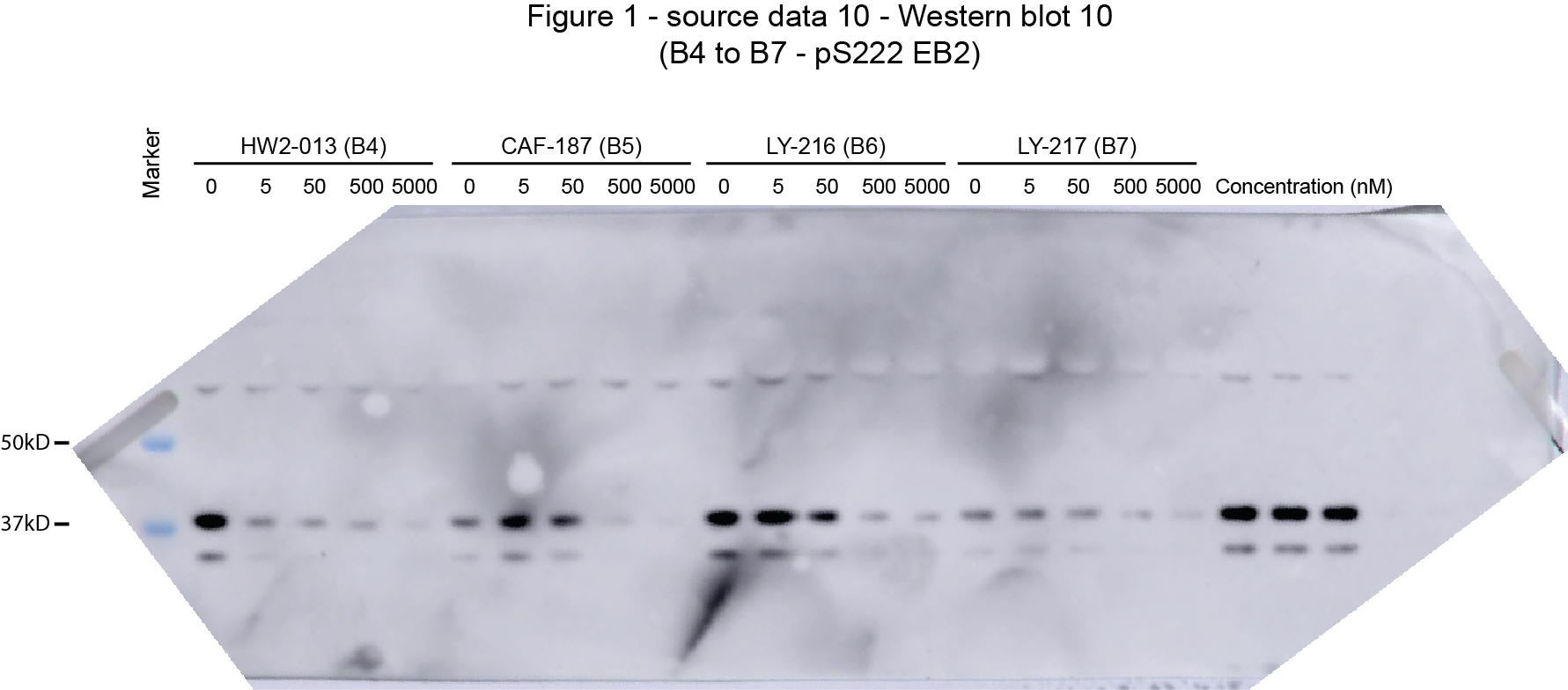

Supplement: Figure 1—source data 1. [file elife-88206-fig1-data1.zip › Figure 1 - source data/Figure 1 - source data 10/Western blot 10 - B4 to B7 - pEB2 - labeled.png]

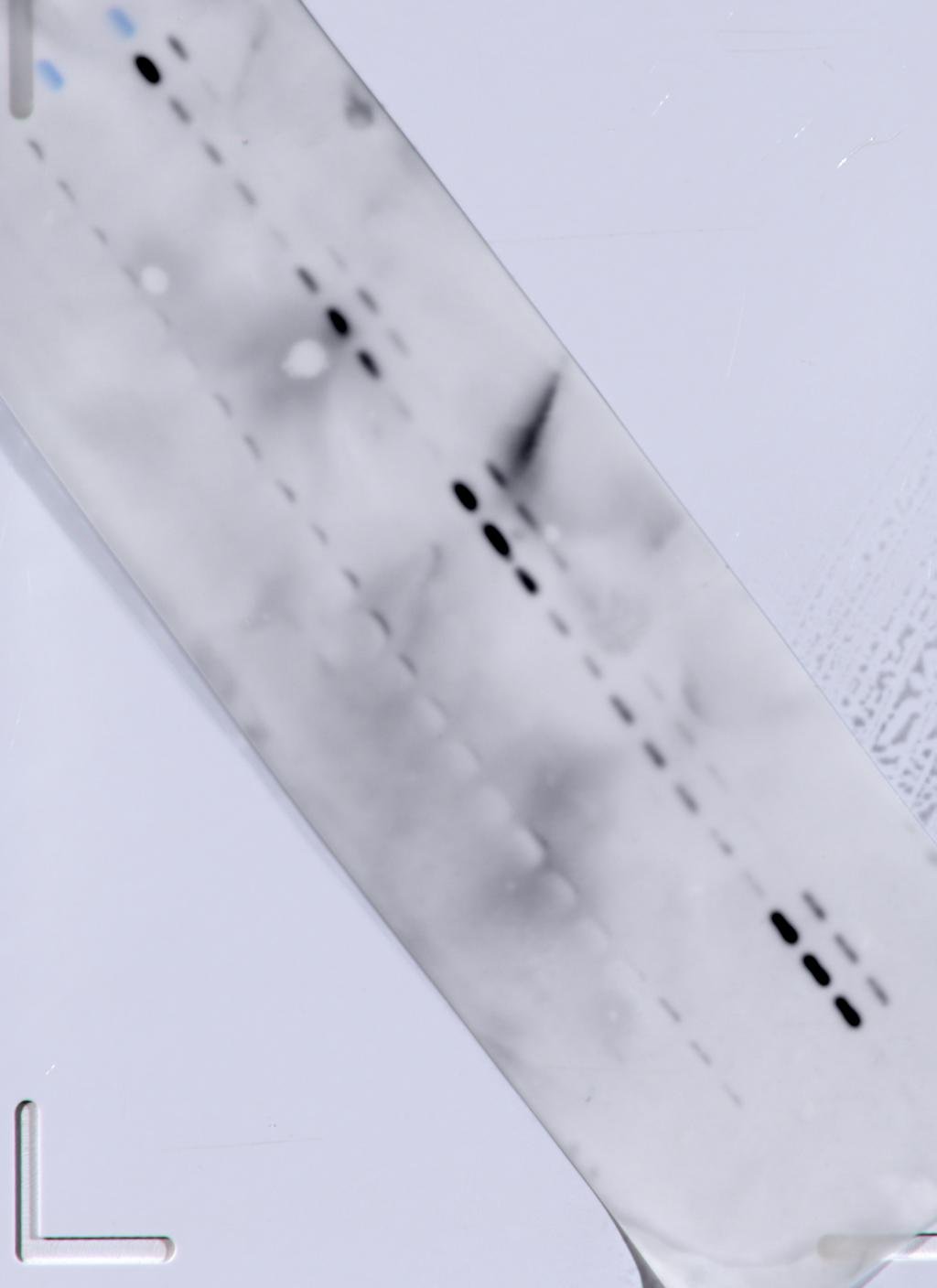

Supplement: Figure 1—source data 1. [file elife-88206-fig1-data1.zip › Figure 1 - source data/Figure 1 - source data 10/Western blot 10 - B4 to B7 - pEB2 - uncropped.jpg]

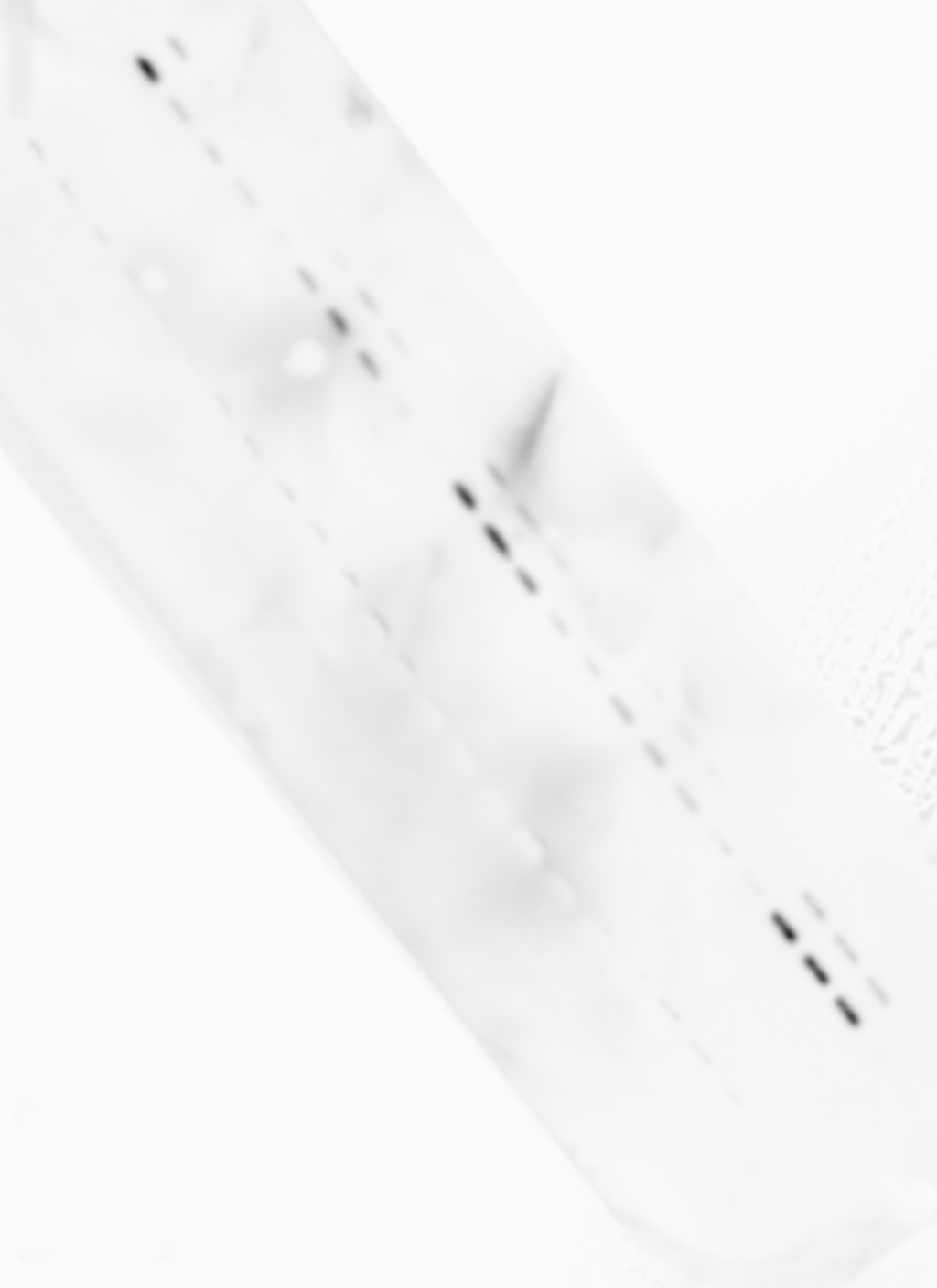

Supplement: Figure 1—source data 1. [file elife-88206-fig1-data1.zip › Figure 1 - source data/Figure 1 - source data 10/Western blot 10 - B4 to B7 - pEB2 - uncropped.tif]

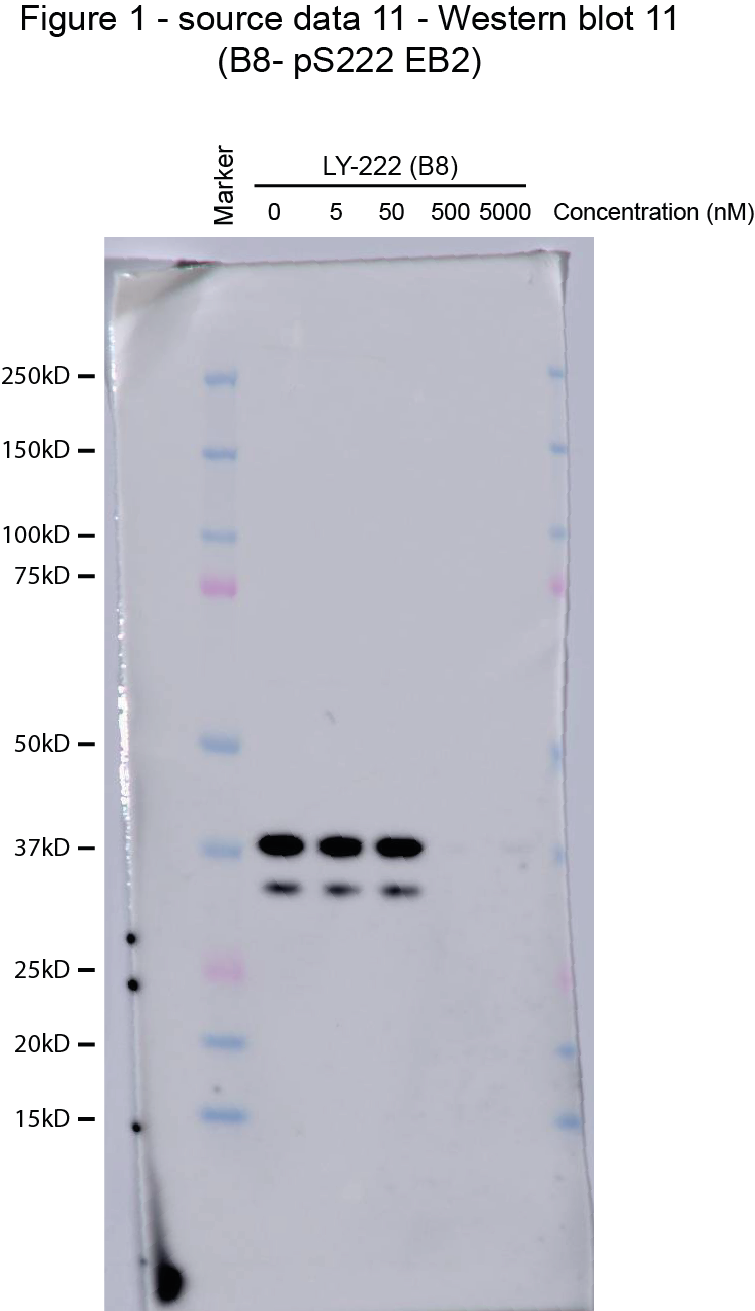

Supplement: Figure 1—source data 1. [file elife-88206-fig1-data1.zip › Figure 1 - source data/Figure 1 - source data 11/Western blot 11 - B8 - pEB2 - labeled.png]

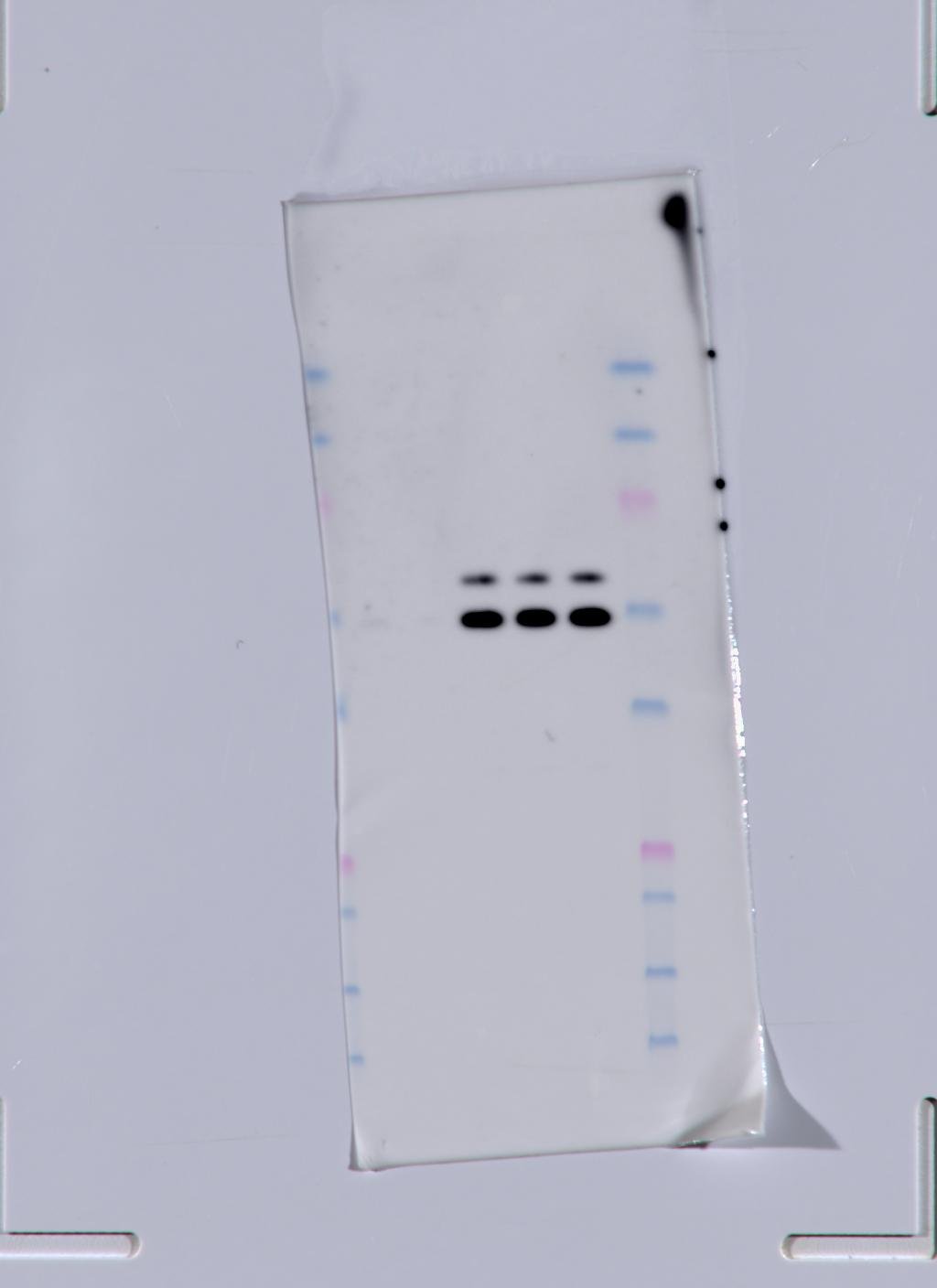

Supplement: Figure 1—source data 1. [file elife-88206-fig1-data1.zip › Figure 1 - source data/Figure 1 - source data 11/Western blot 11 - B8 - pEB2 - uncropped.jpg]

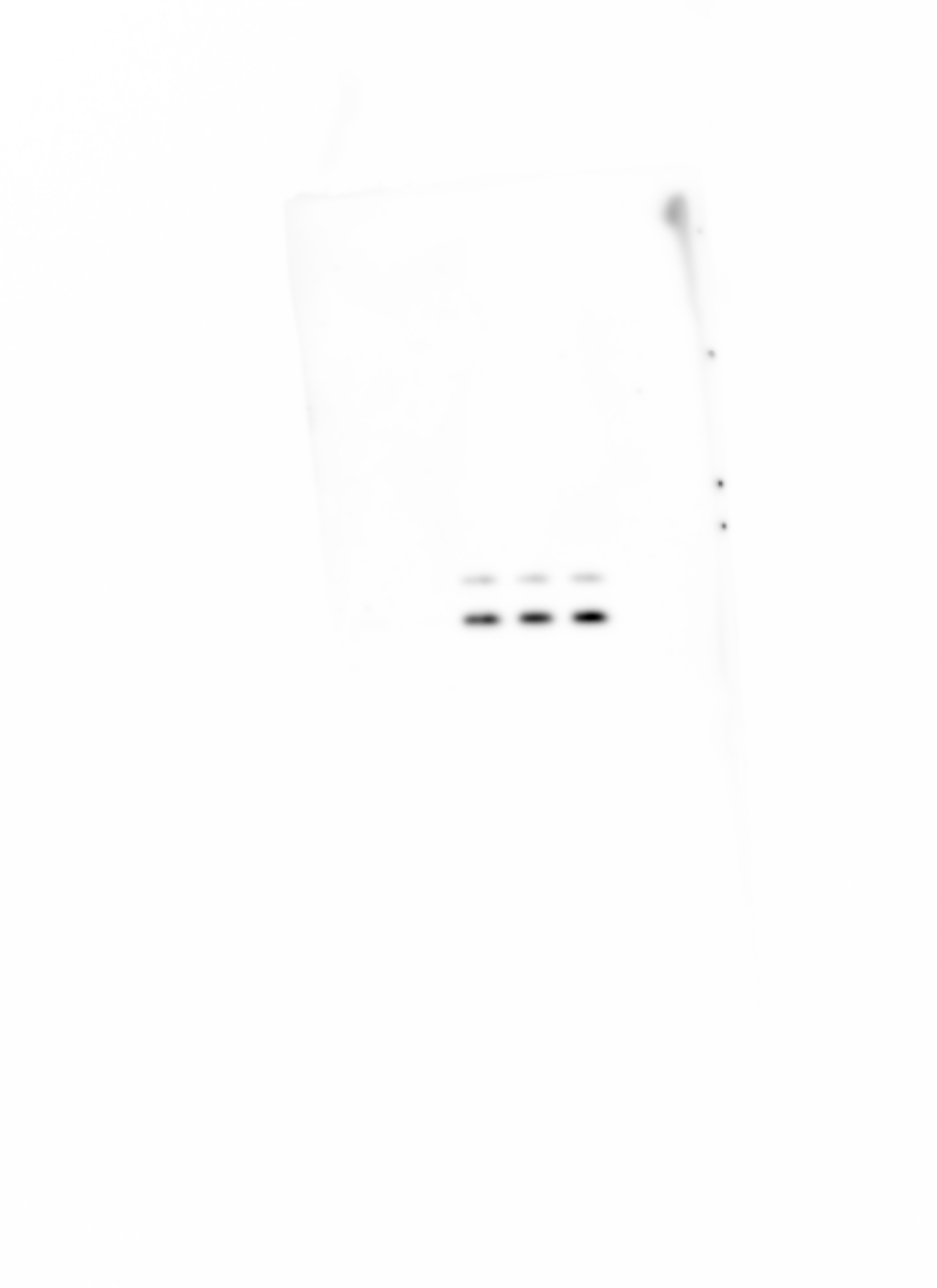

Supplement: Figure 1—source data 1. [file elife-88206-fig1-data1.zip › Figure 1 - source data/Figure 1 - source data 11/Western blot 11 - B8 - pEB2 - uncropped.tif]

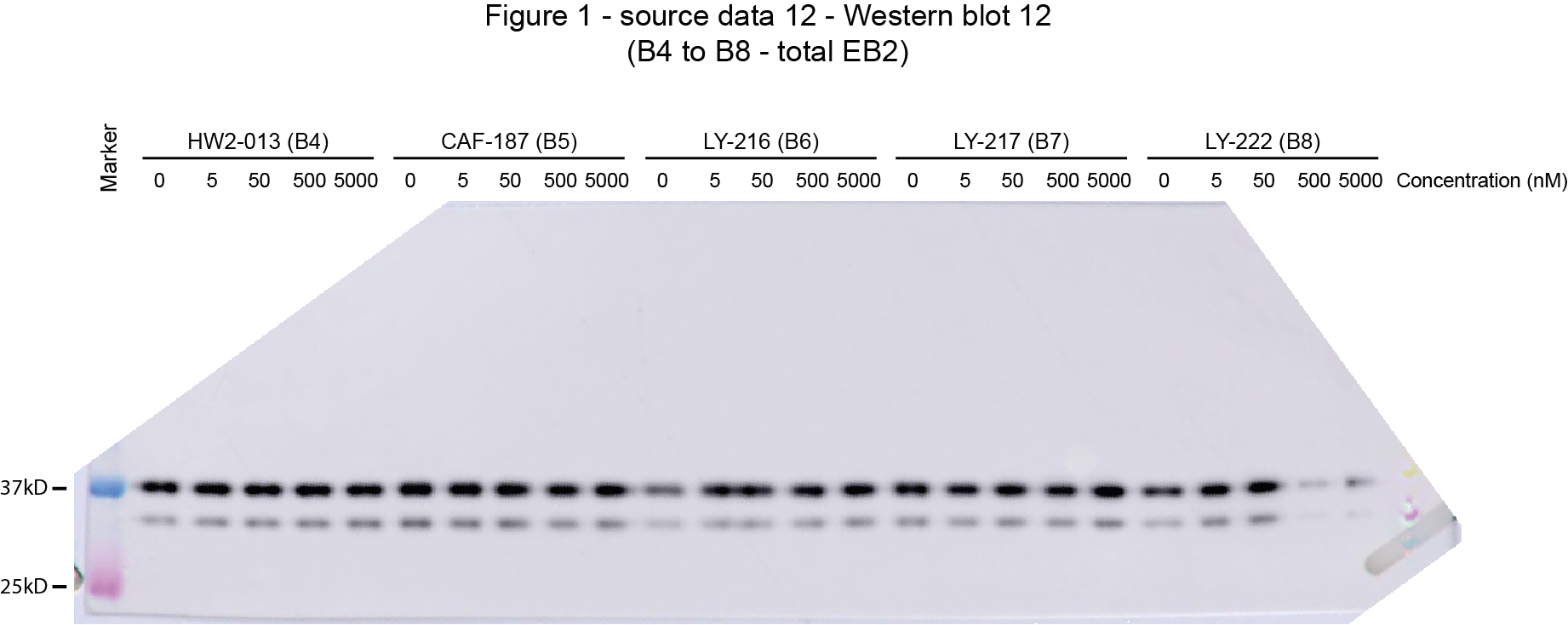

Supplement: Figure 1—source data 1. [file elife-88206-fig1-data1.zip › Figure 1 - source data/Figure 1 - source data 12/Western blot 12 - B4 to B8 - total EB2 - labeled.png]

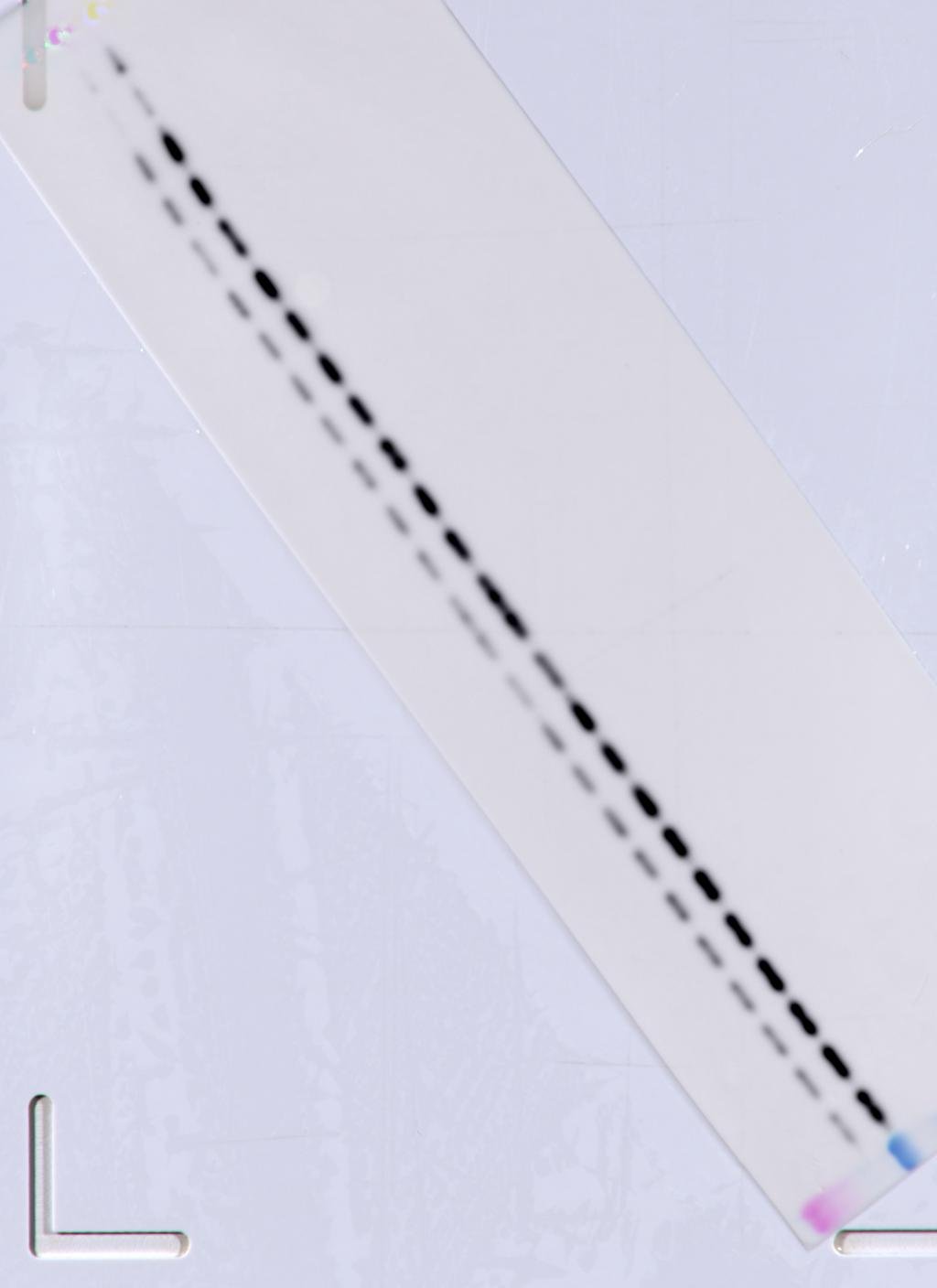

Supplement: Figure 1—source data 1. [file elife-88206-fig1-data1.zip › Figure 1 - source data/Figure 1 - source data 12/Western blot 12 - B4 to B8 - total EB2 - uncropped.jpg]

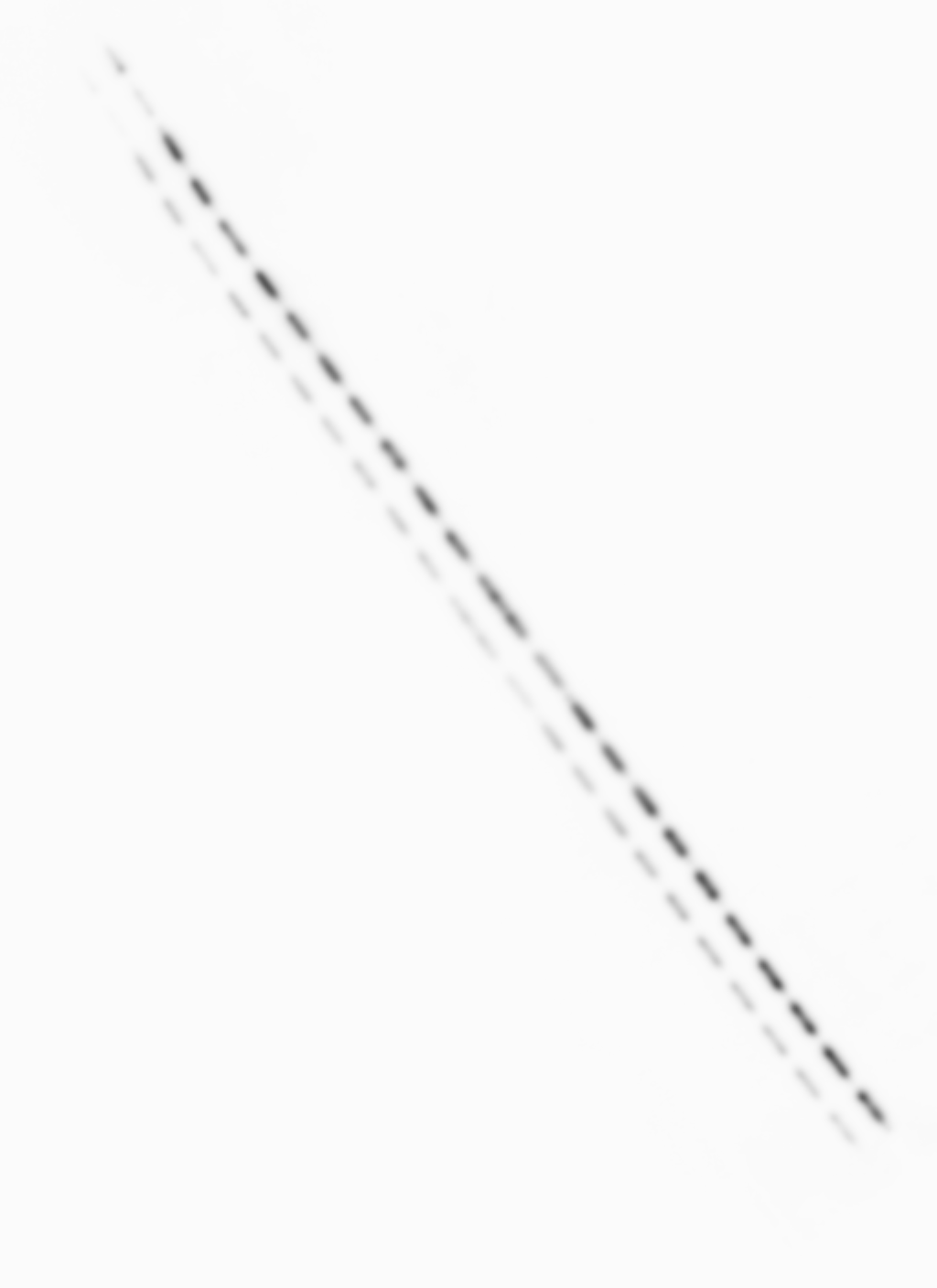

Supplement: Figure 1—source data 1. [file elife-88206-fig1-data1.zip › Figure 1 - source data/Figure 1 - source data 12/Western blot 12 - B4 to B8 - total EB2 - uncropped.tif]

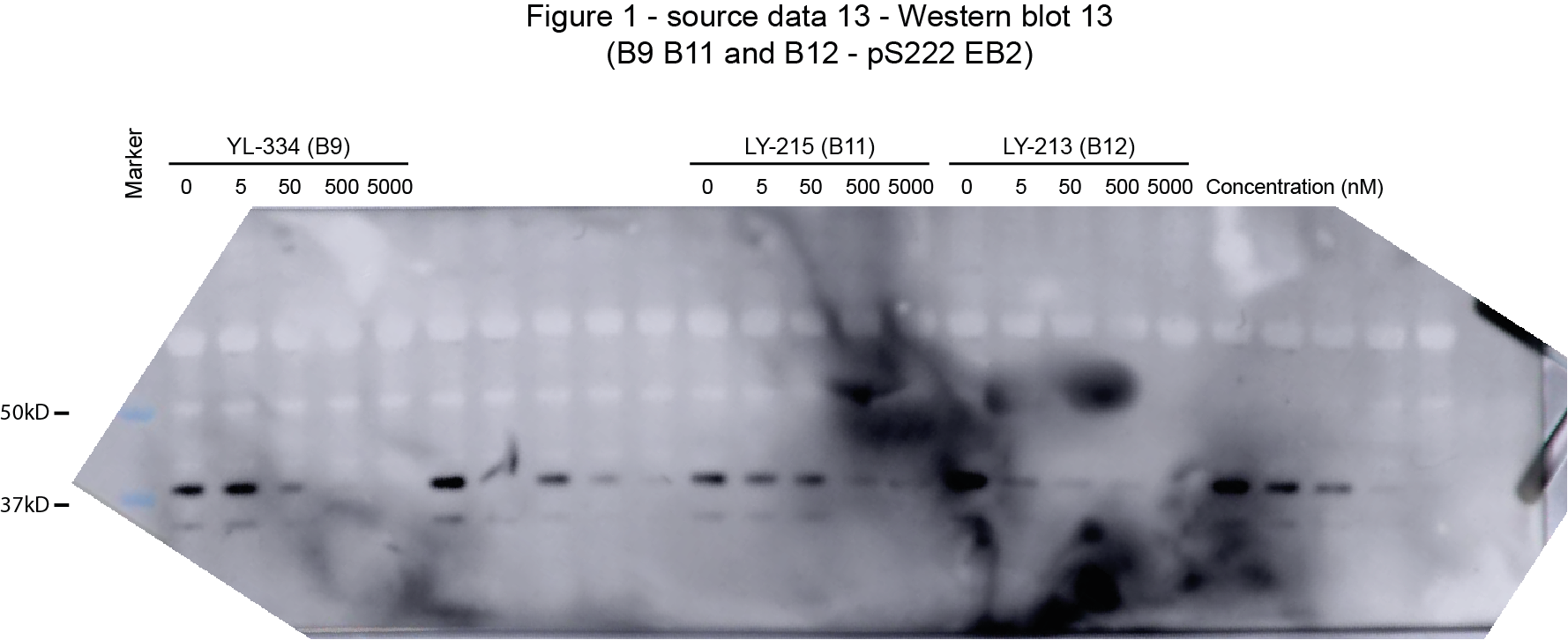

Supplement: Figure 1—source data 1. [file elife-88206-fig1-data1.zip › Figure 1 - source data/Figure 1 - source data 13/Western blot 13 - B9 B11 B12 - pEB2 - labeled.png]

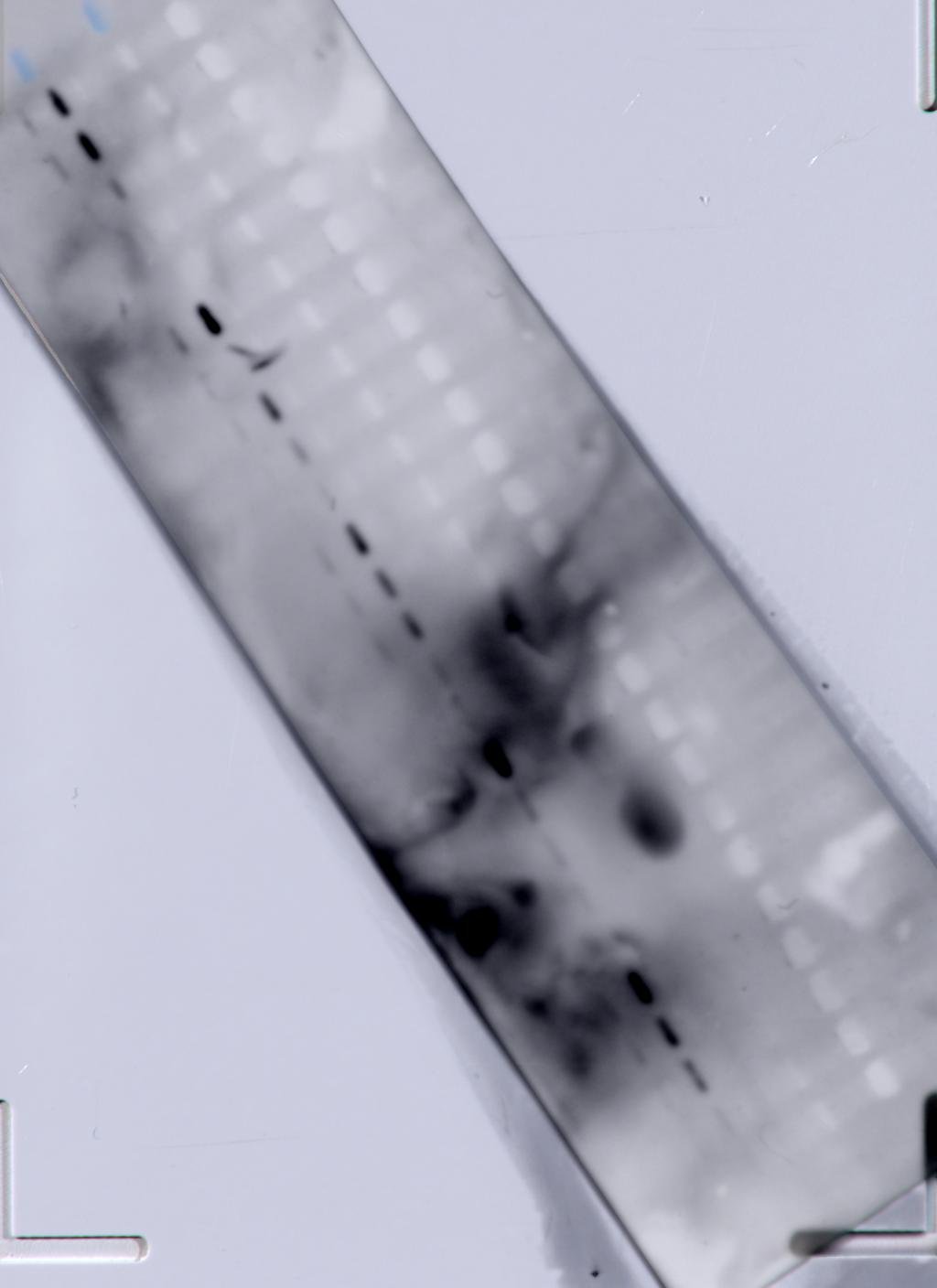

Supplement: Figure 1—source data 1. [file elife-88206-fig1-data1.zip › Figure 1 - source data/Figure 1 - source data 13/Western blot 13 - B9 B11 B12 - pEB2 - uncropped.jpg]

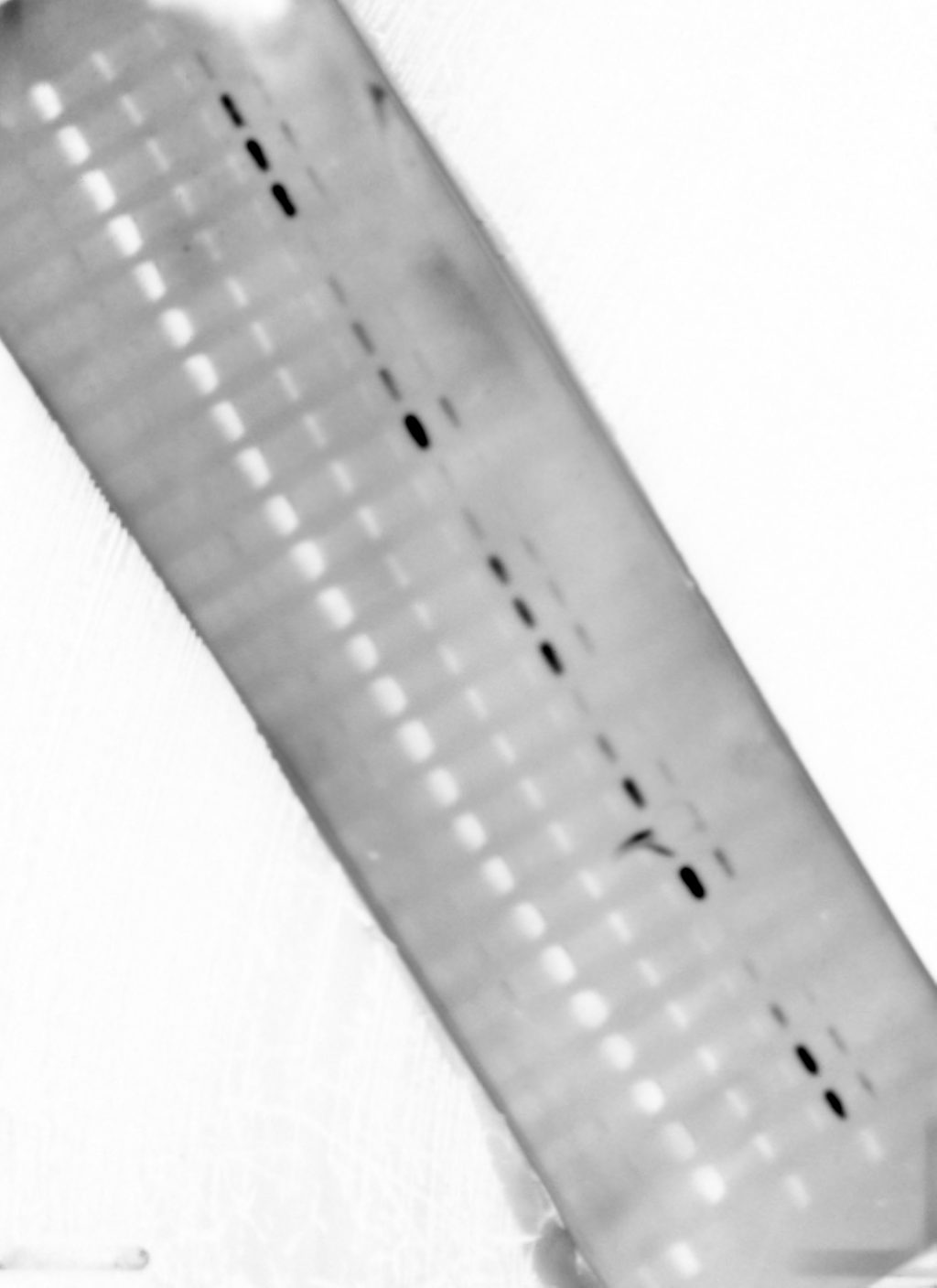

Supplement: Figure 1—source data 1. [file elife-88206-fig1-data1.zip › Figure 1 - source data/Figure 1 - source data 13/Western blot 13 - B9 B11 B12 - pEB2 - uncropped.tif]

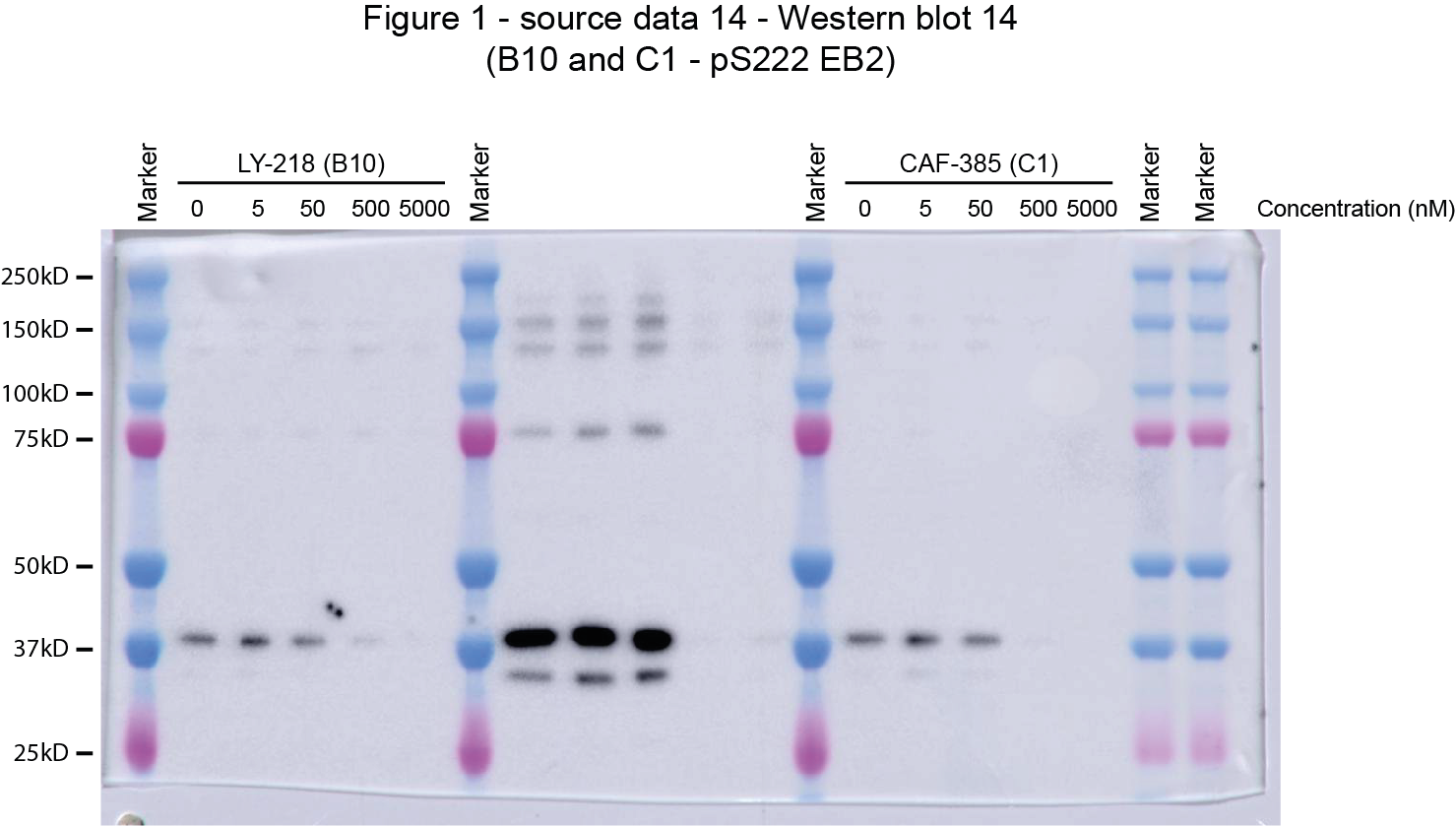

Supplement: Figure 1—source data 1. [file elife-88206-fig1-data1.zip › Figure 1 - source data/Figure 1 - source data 14/Western bloy 14 - B10 and C1 - pEB2 - labeled.png]

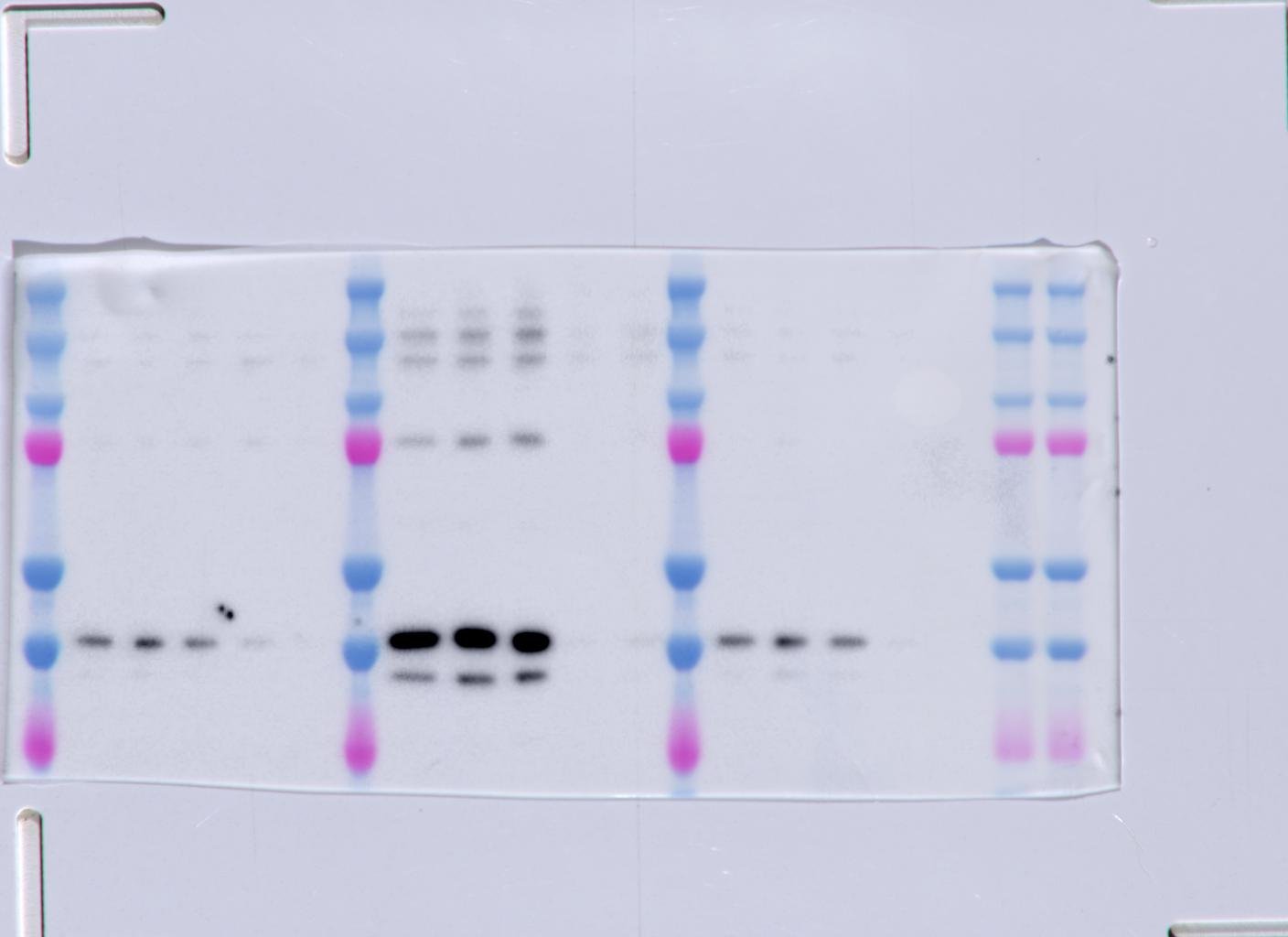

Supplement: Figure 1—source data 1. [file elife-88206-fig1-data1.zip › Figure 1 - source data/Figure 1 - source data 14/Western bloy 14 - B10 and C1 - pEB2 - uncropped.jpg]

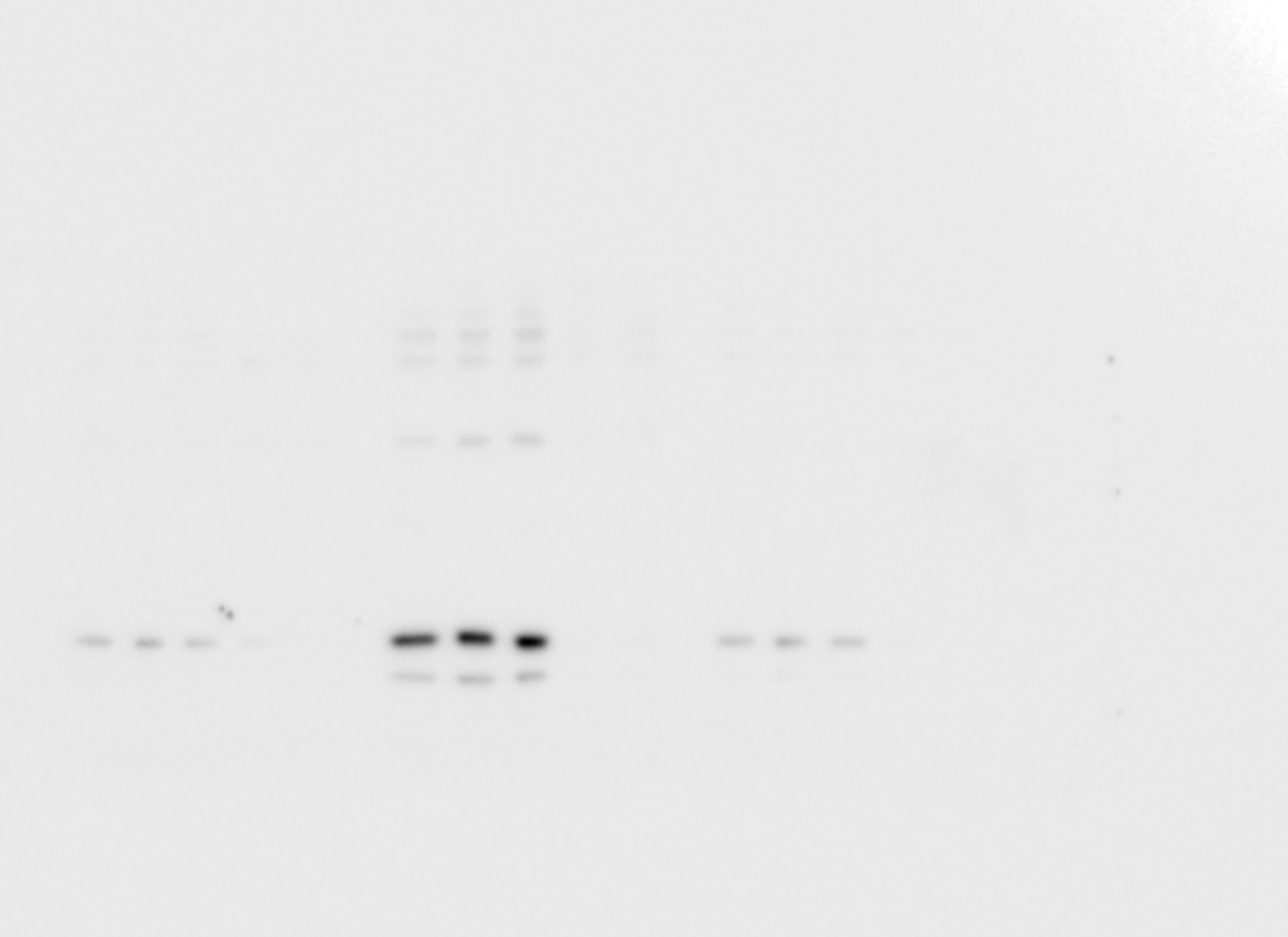

Supplement: Figure 1—source data 1. [file elife-88206-fig1-data1.zip › Figure 1 - source data/Figure 1 - source data 14/Western bloy 14 - B10 and C1 - pEB2 - uncropped.tif]

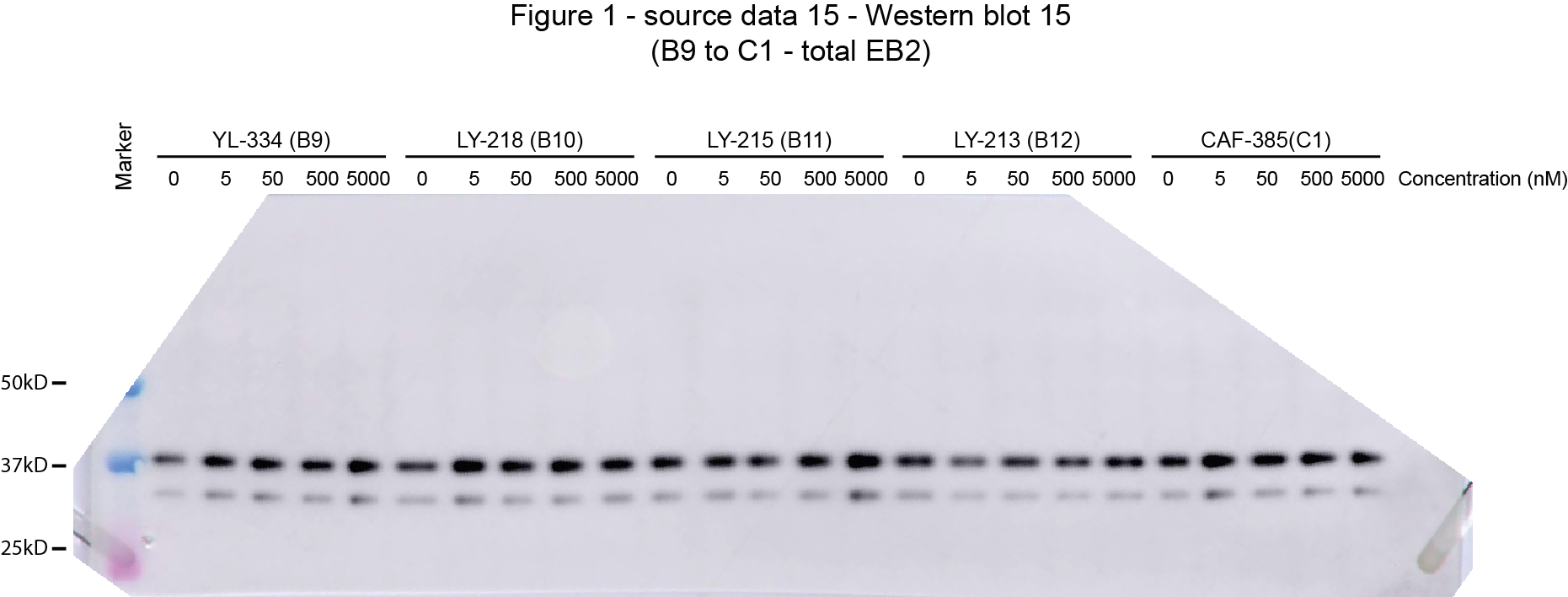

Supplement: Figure 1—source data 1. [file elife-88206-fig1-data1.zip › Figure 1 - source data/Figure 1 - source data 15/Western blot 15 - B9 to C1 - total EB2 - labeled.png]

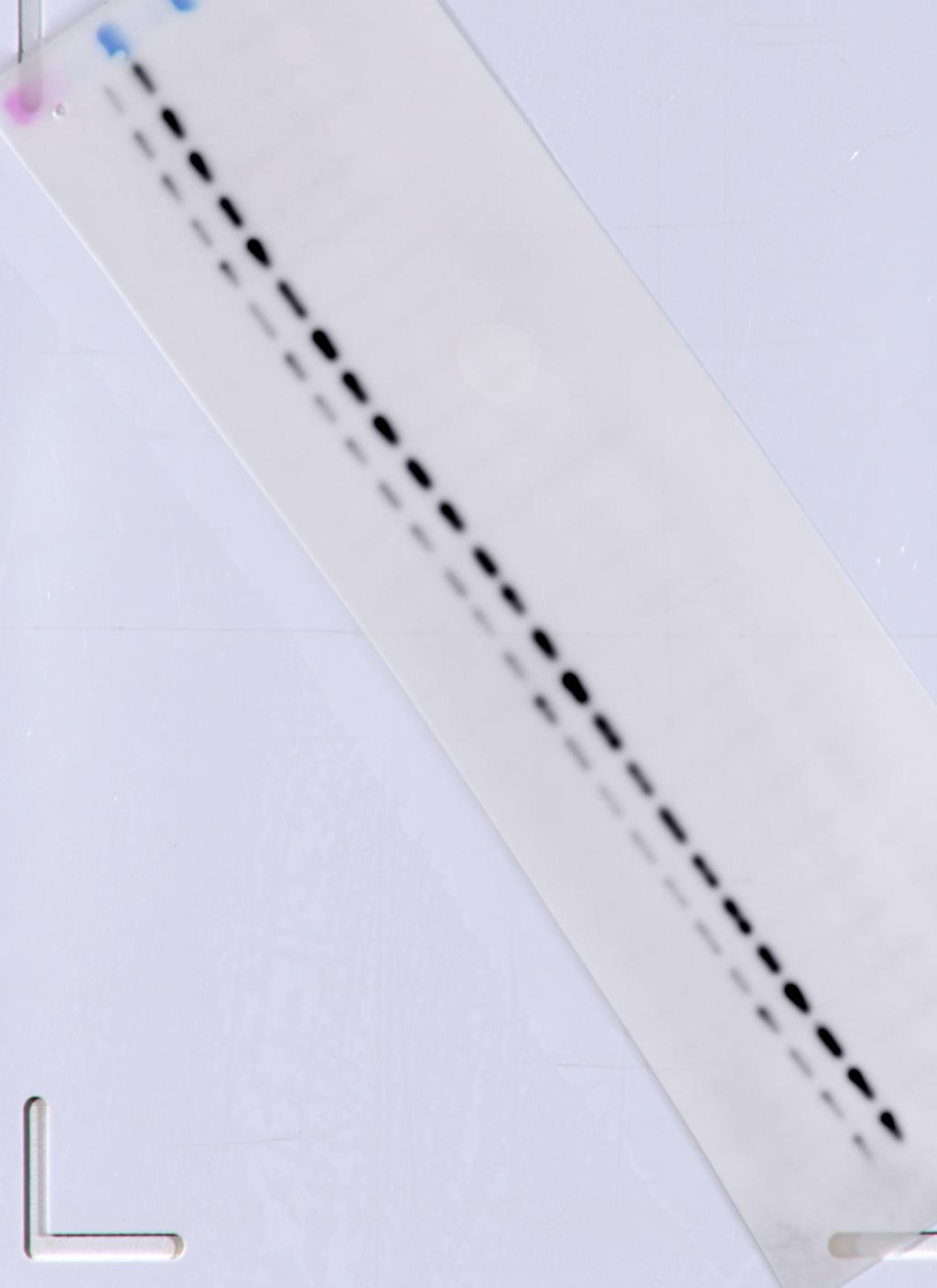

Supplement: Figure 1—source data 1. [file elife-88206-fig1-data1.zip › Figure 1 - source data/Figure 1 - source data 15/Western blot 15 - B9 to C1 - total EB2 - uncropped.jpg]

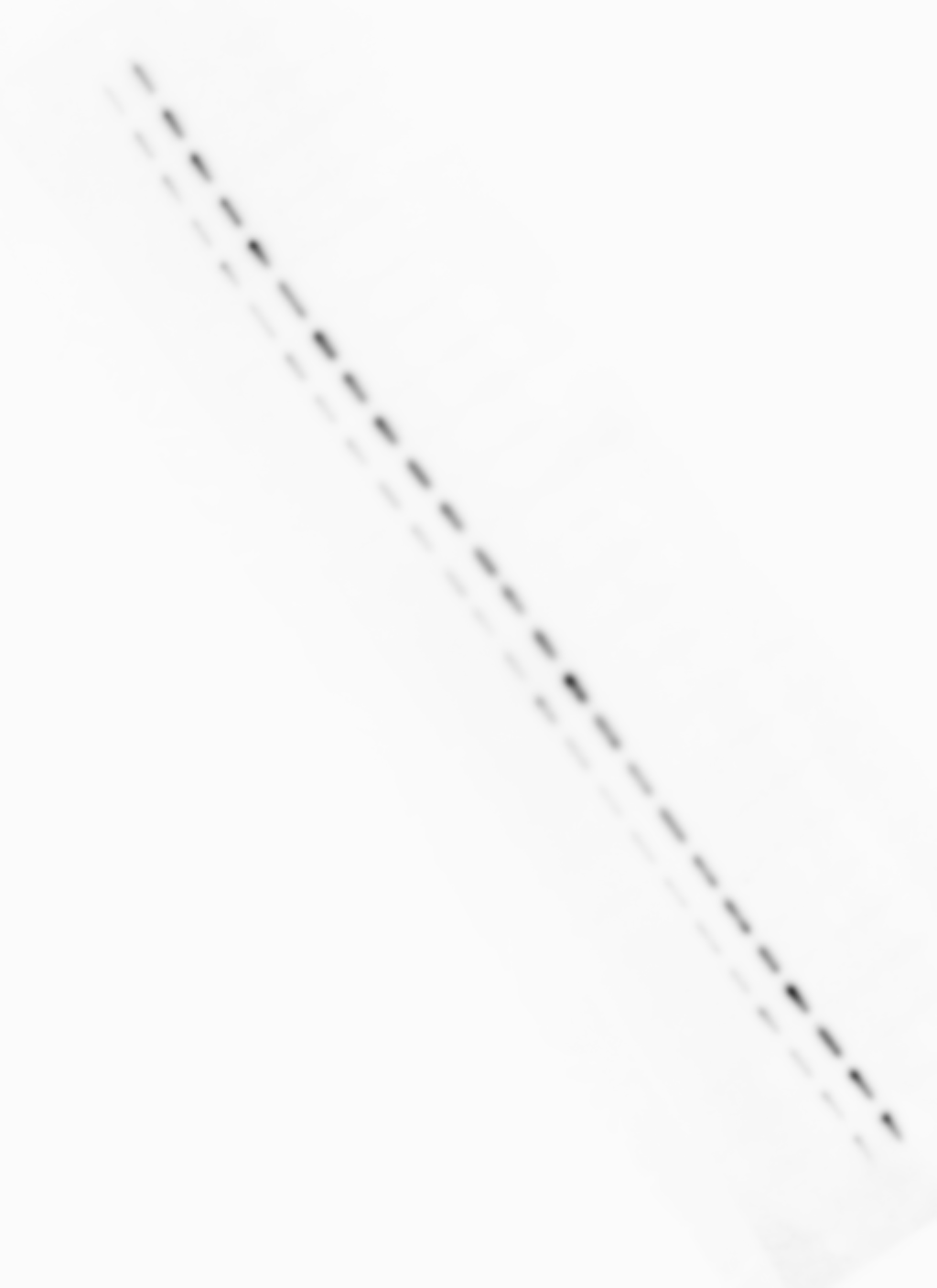

Supplement: Figure 1—source data 1. [file elife-88206-fig1-data1.zip › Figure 1 - source data/Figure 1 - source data 15/Western blot 15 - B9 to C1 - total EB2 - uncropped.tif]

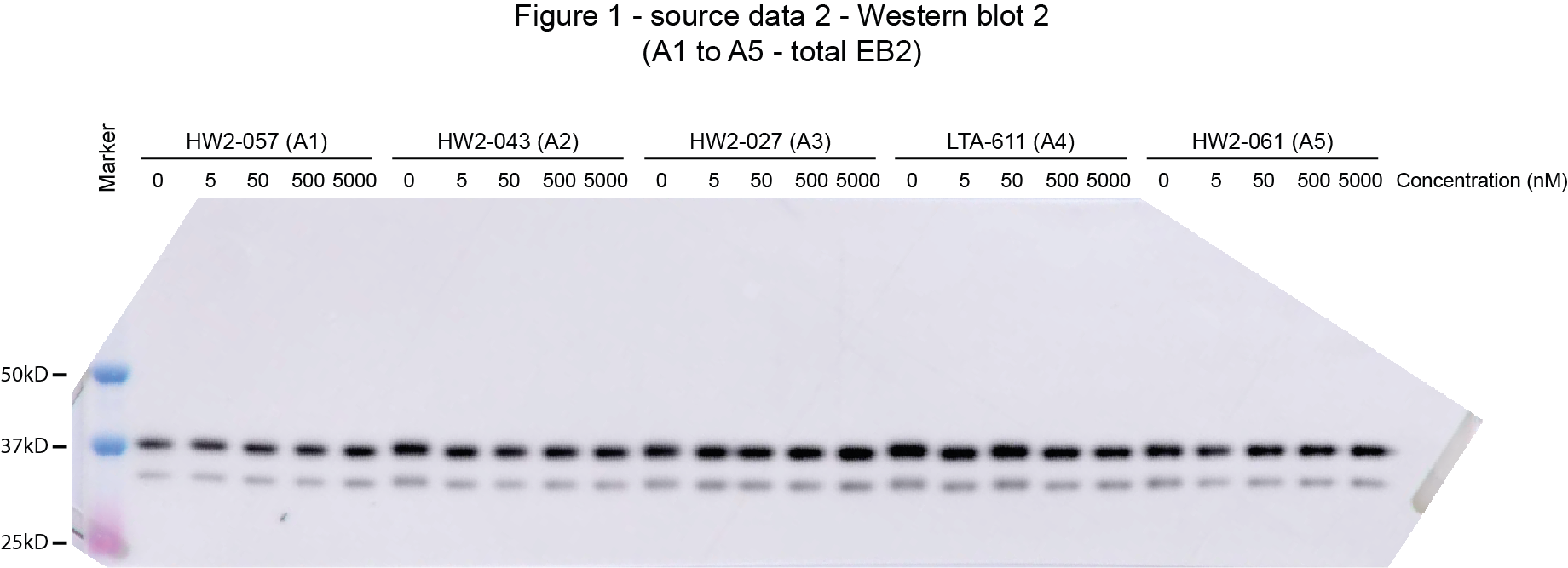

Supplement: Figure 1—source data 1. [file elife-88206-fig1-data1.zip › Figure 1 - source data/Figure 1 - source data 2/Western blot 2 - A1 to A5 - total EB2 - labeled.png]

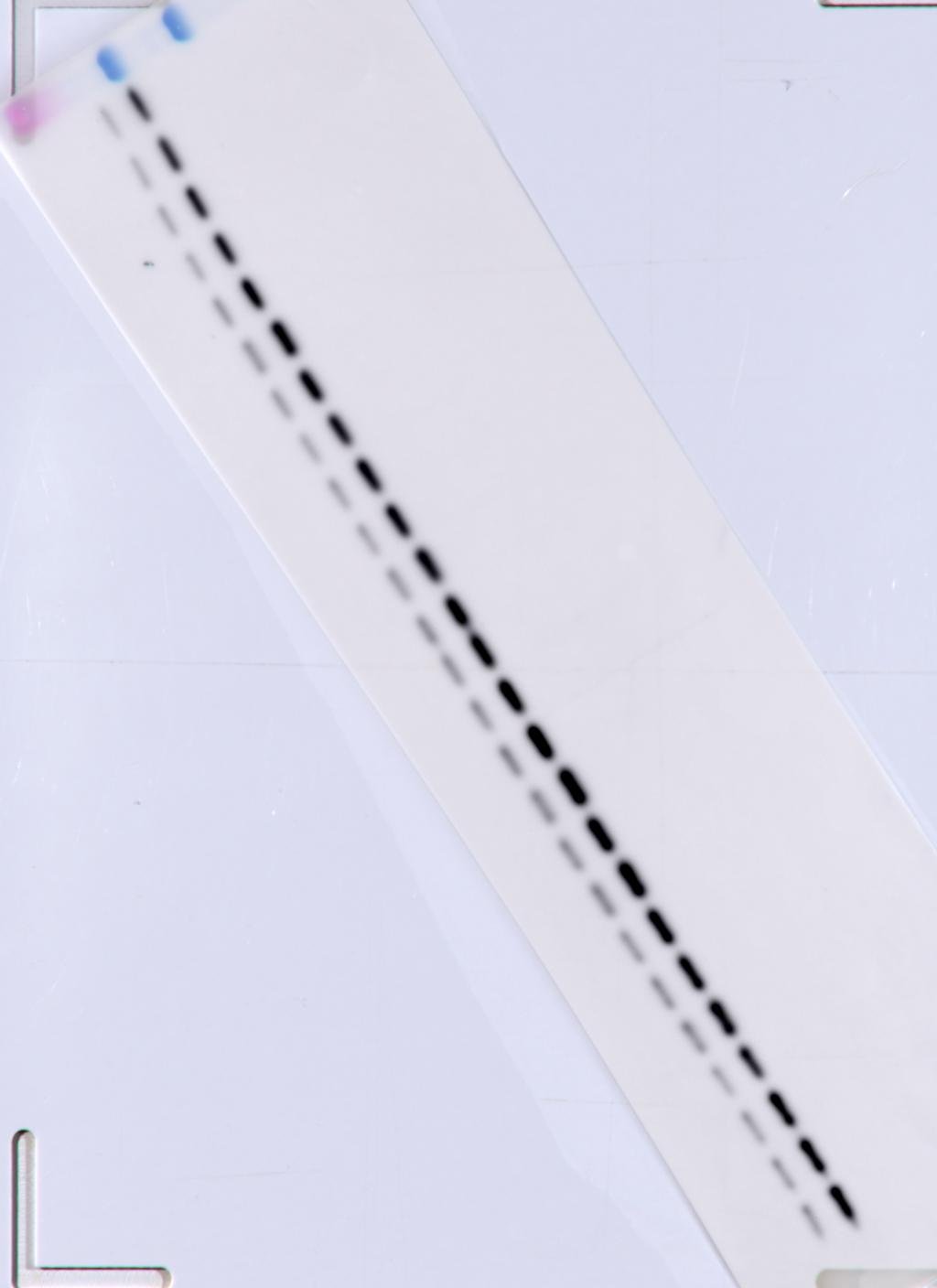

Supplement: Figure 1—source data 1. [file elife-88206-fig1-data1.zip › Figure 1 - source data/Figure 1 - source data 2/Western blot 2 - A1 to A5 - total EB2 - uncropped.jpg]

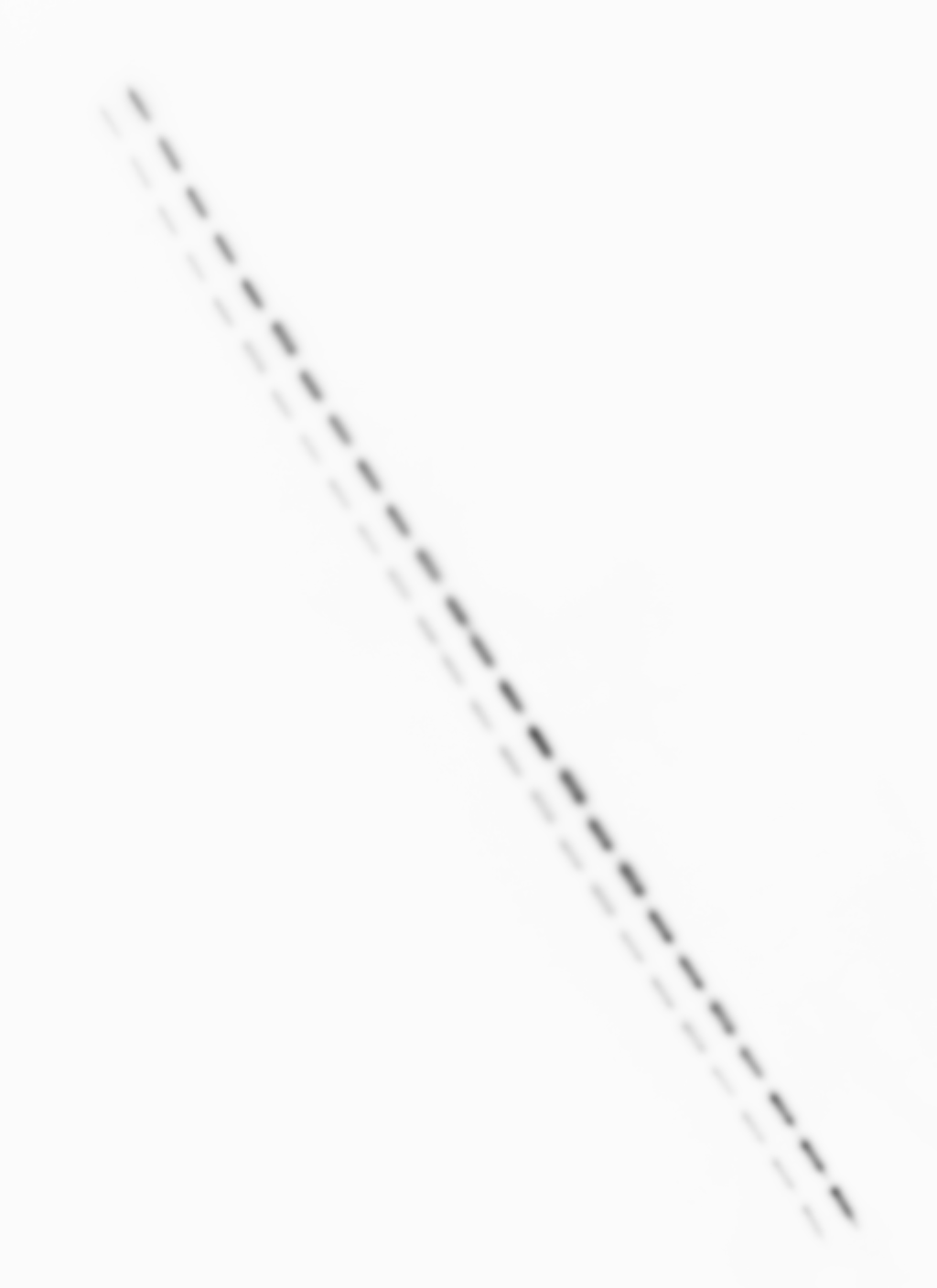

Supplement: Figure 1—source data 1. [file elife-88206-fig1-data1.zip › Figure 1 - source data/Figure 1 - source data 2/Western blot 2 - A1 to A5 - total EB2 - uncropped.tif]

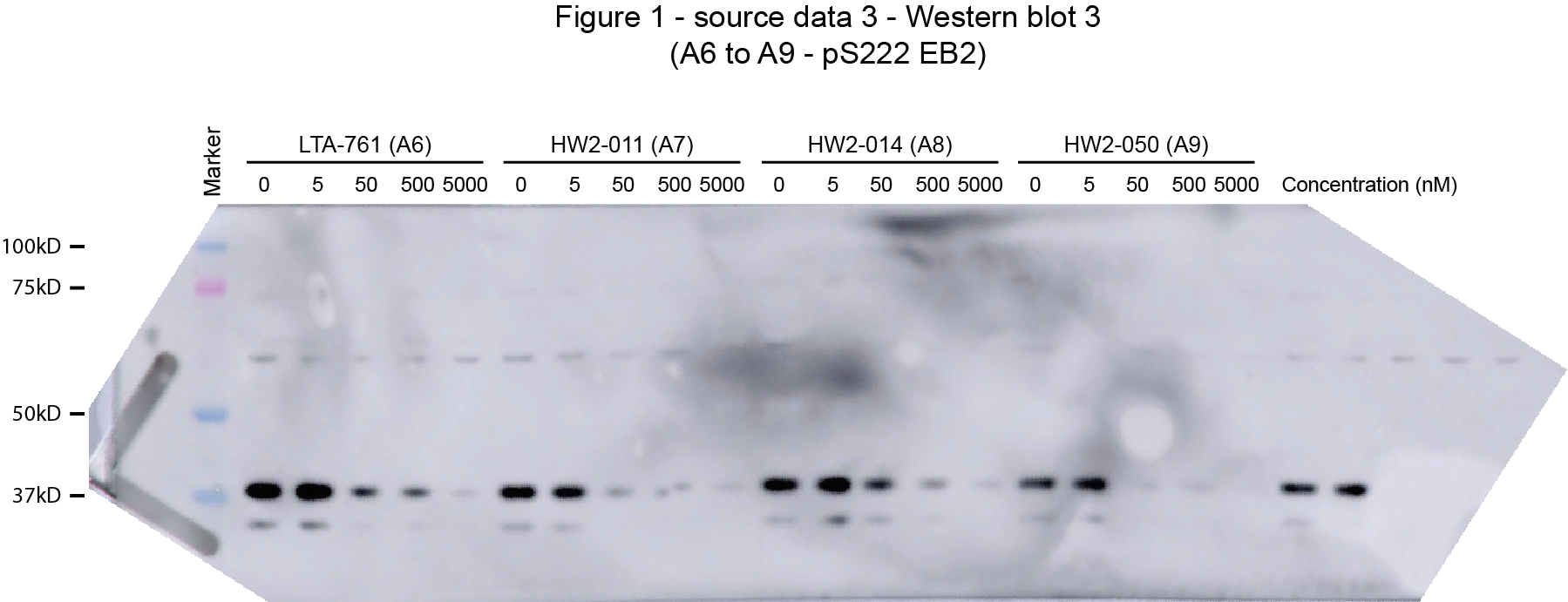

Supplement: Figure 1—source data 1. [file elife-88206-fig1-data1.zip › Figure 1 - source data/Figure 1 - source data 3/Western blot 3 - A6 to A9 - pEB2 - labeled.png]

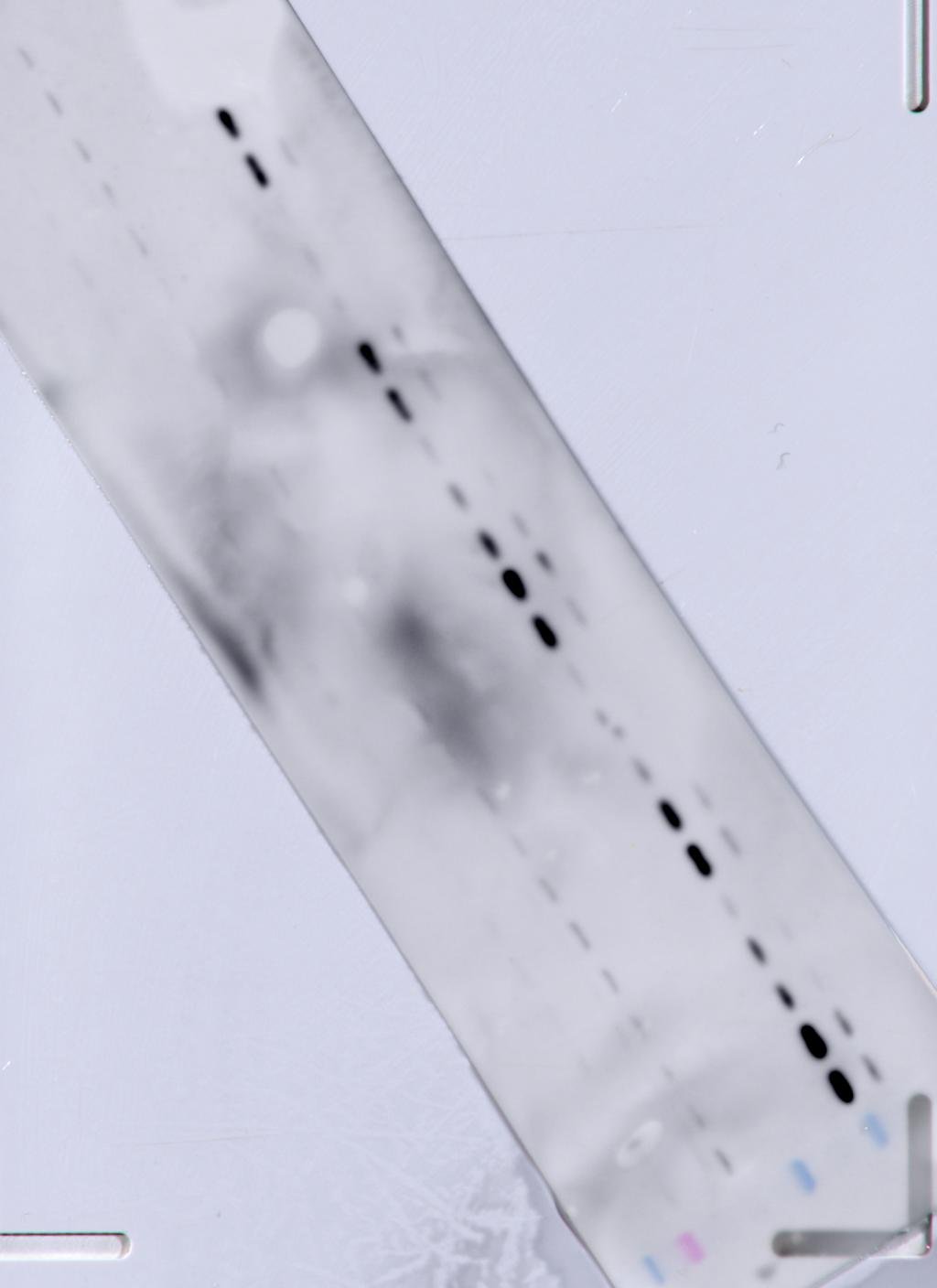

Supplement: Figure 1—source data 1. [file elife-88206-fig1-data1.zip › Figure 1 - source data/Figure 1 - source data 3/Western blot 3 - A6 to A9 - pEB2 - uncropped.jpg]

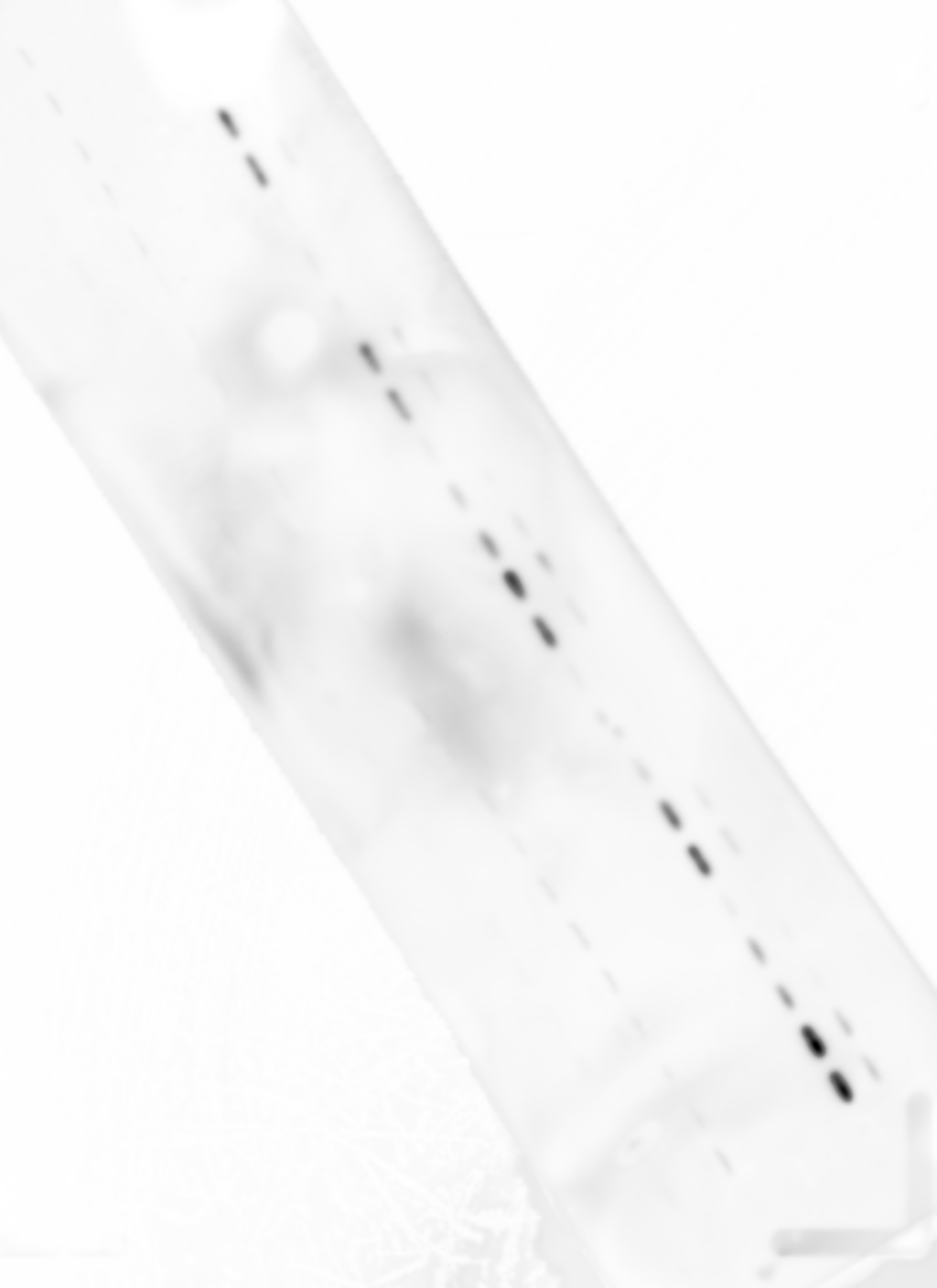

Supplement: Figure 1—source data 1. [file elife-88206-fig1-data1.zip › Figure 1 - source data/Figure 1 - source data 3/Western blot 3 - A6 to A9 - pEB2 - uncropped.tif]

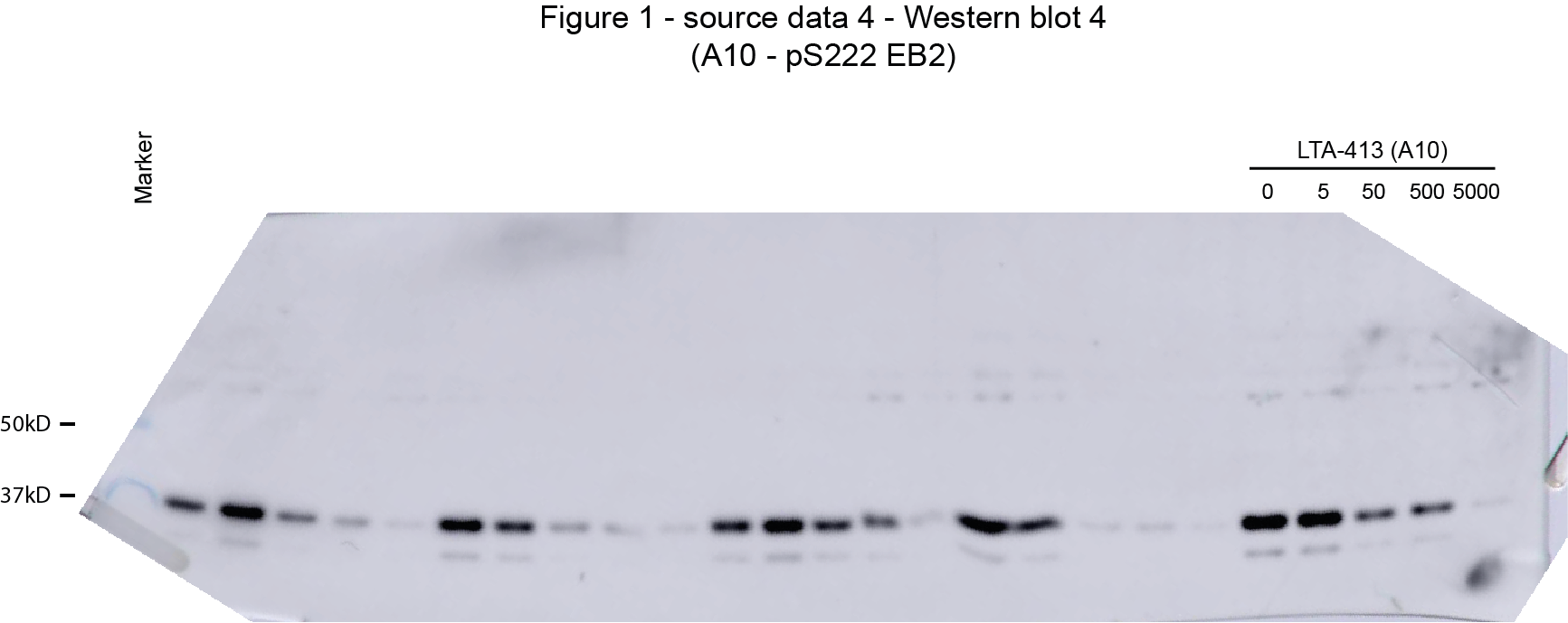

Supplement: Figure 1—source data 1. [file elife-88206-fig1-data1.zip › Figure 1 - source data/Figure 1 - source data 4/Western blot 4 - A10 - pEB2 - labeled.png]

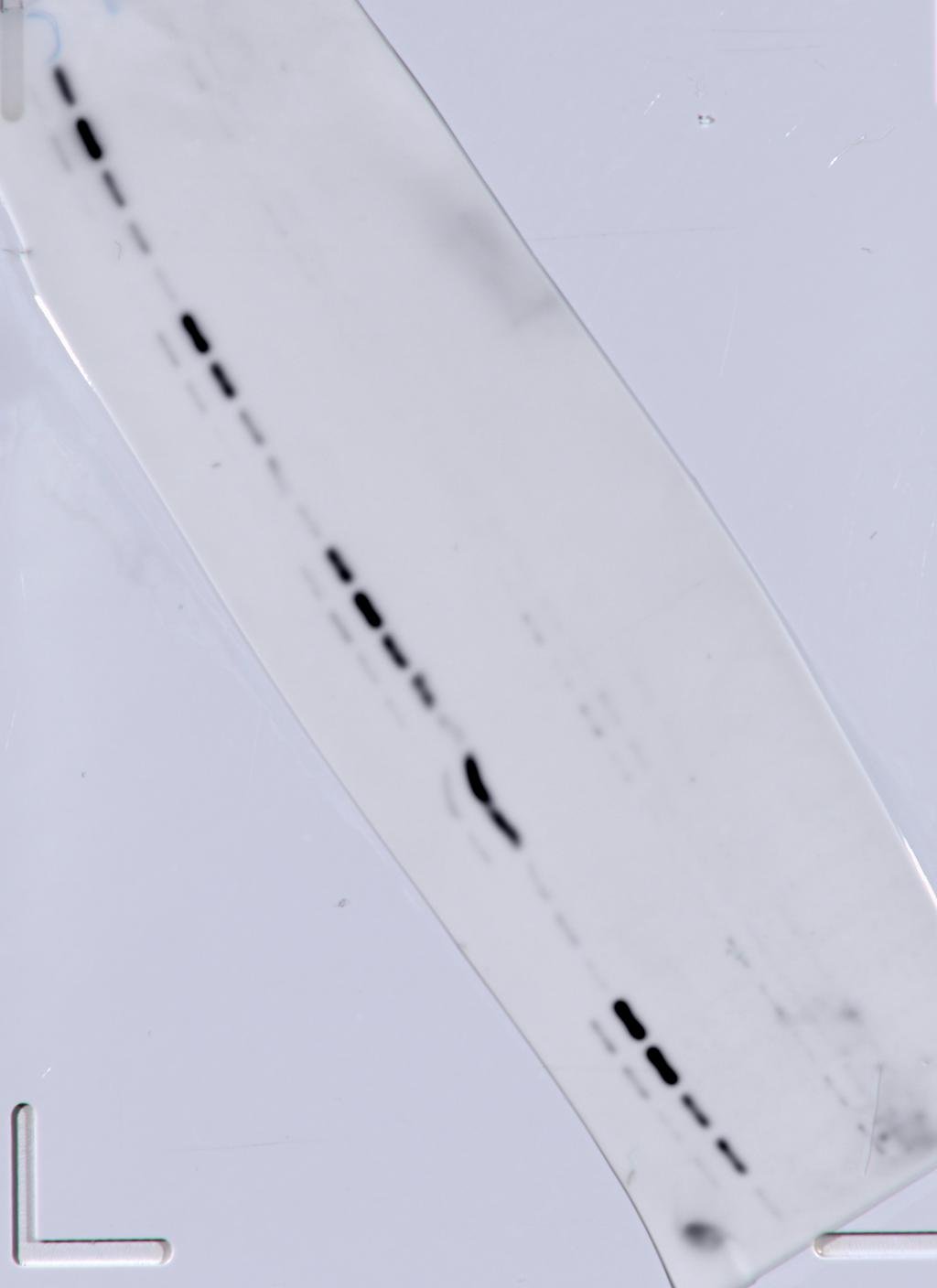

Supplement: Figure 1—source data 1. [file elife-88206-fig1-data1.zip › Figure 1 - source data/Figure 1 - source data 4/Western blot 4 - A10 - pEB2 - uncropped.jpg]

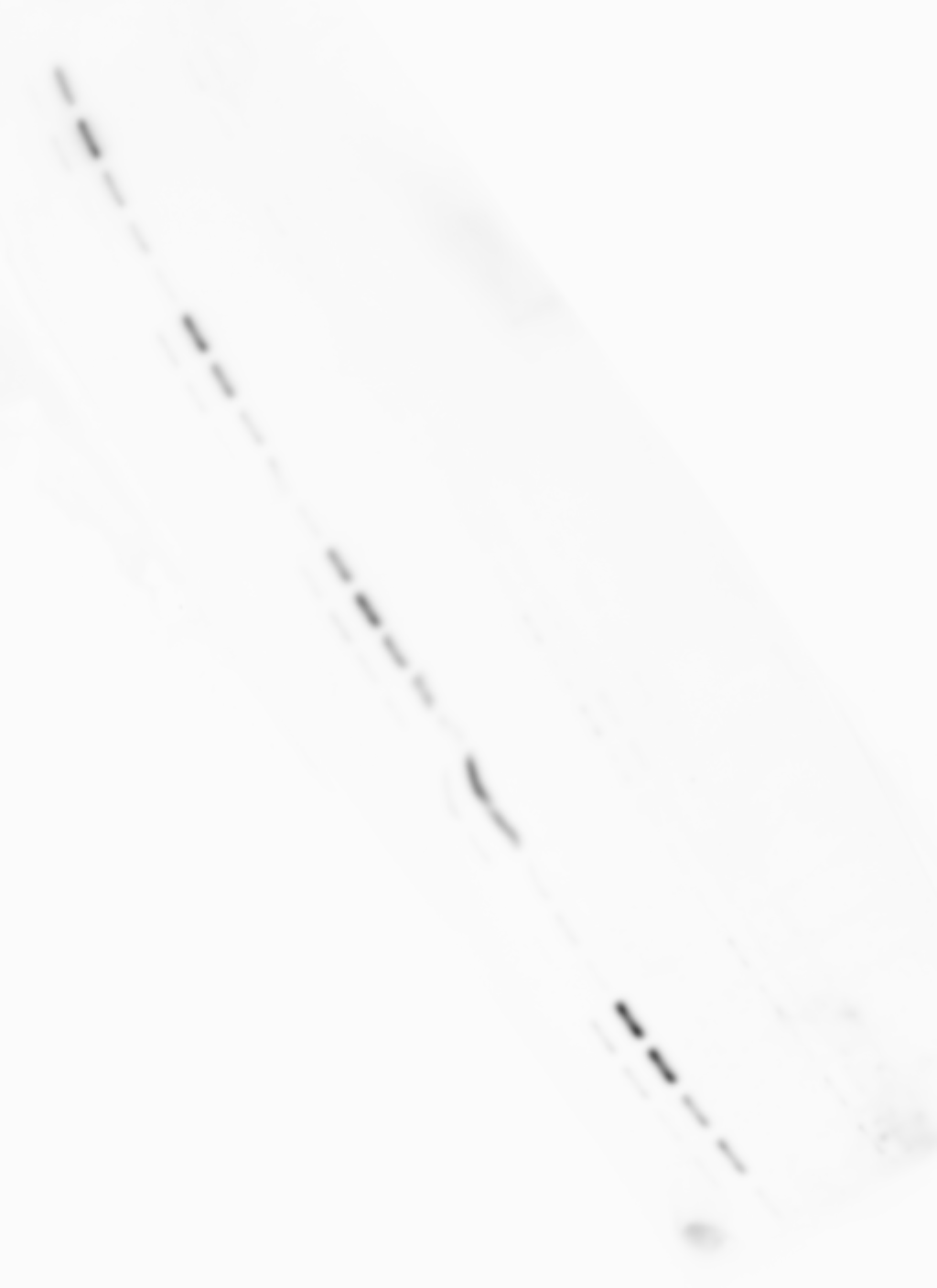

Supplement: Figure 1—source data 1. [file elife-88206-fig1-data1.zip › Figure 1 - source data/Figure 1 - source data 4/Western blot 4 - A10 - pEB2 - uncropped.tif]

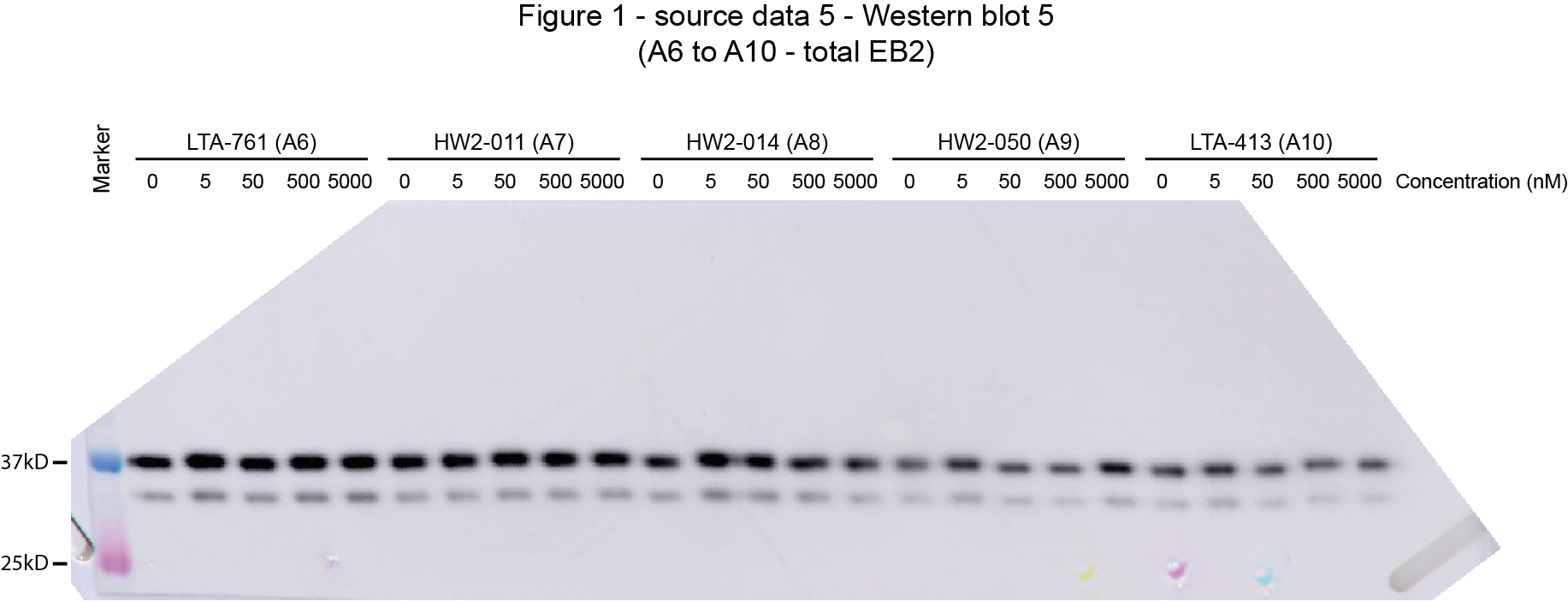

Supplement: Figure 1—source data 1. [file elife-88206-fig1-data1.zip › Figure 1 - source data/Figure 1 - source data 5/Western blot 5 - A6 to A10 - total EB2 - labeled.png]

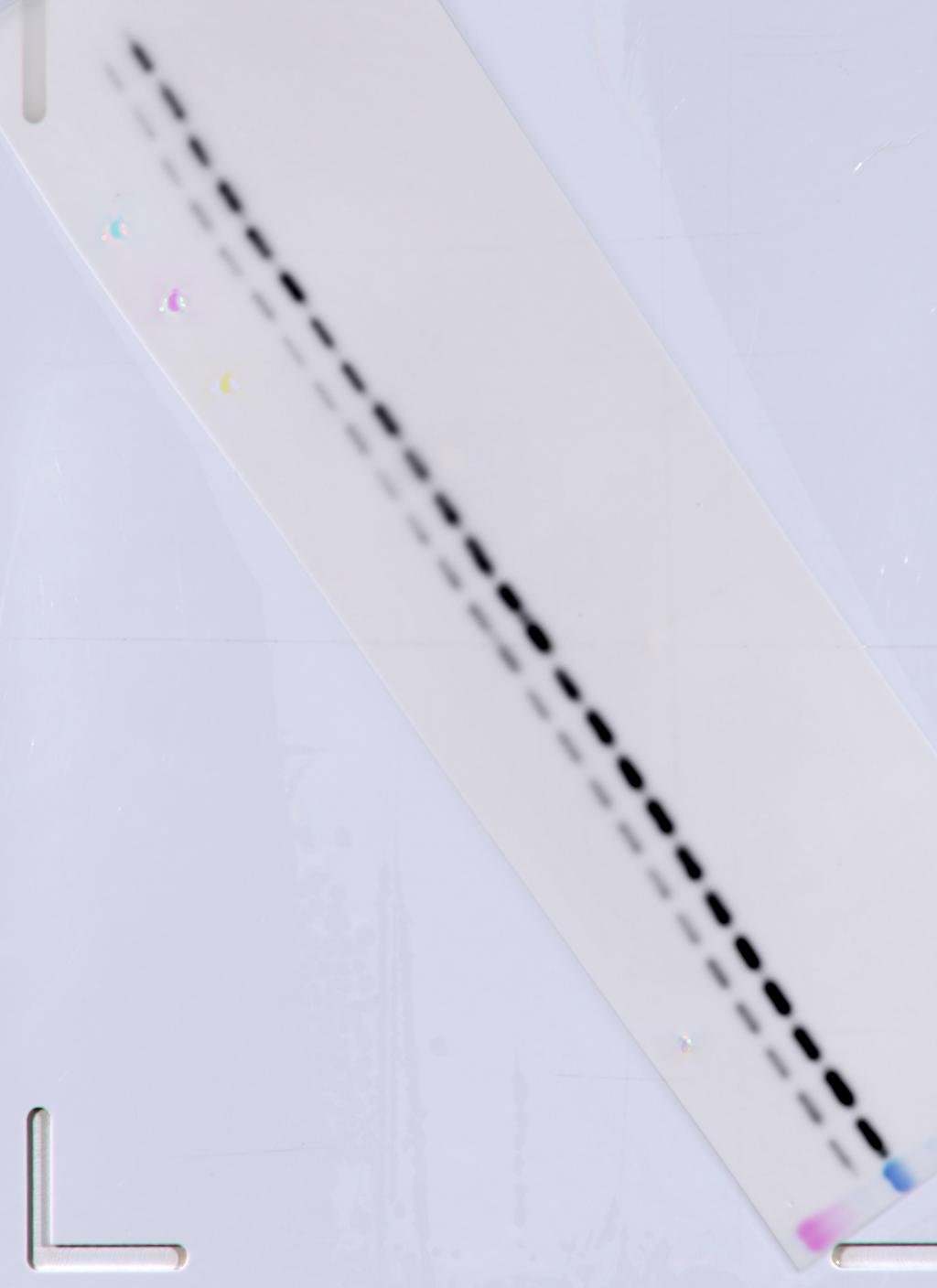

Supplement: Figure 1—source data 1. [file elife-88206-fig1-data1.zip › Figure 1 - source data/Figure 1 - source data 5/Western blot 5 - A6 to A10 - total EB2 - uncropped.jpg]

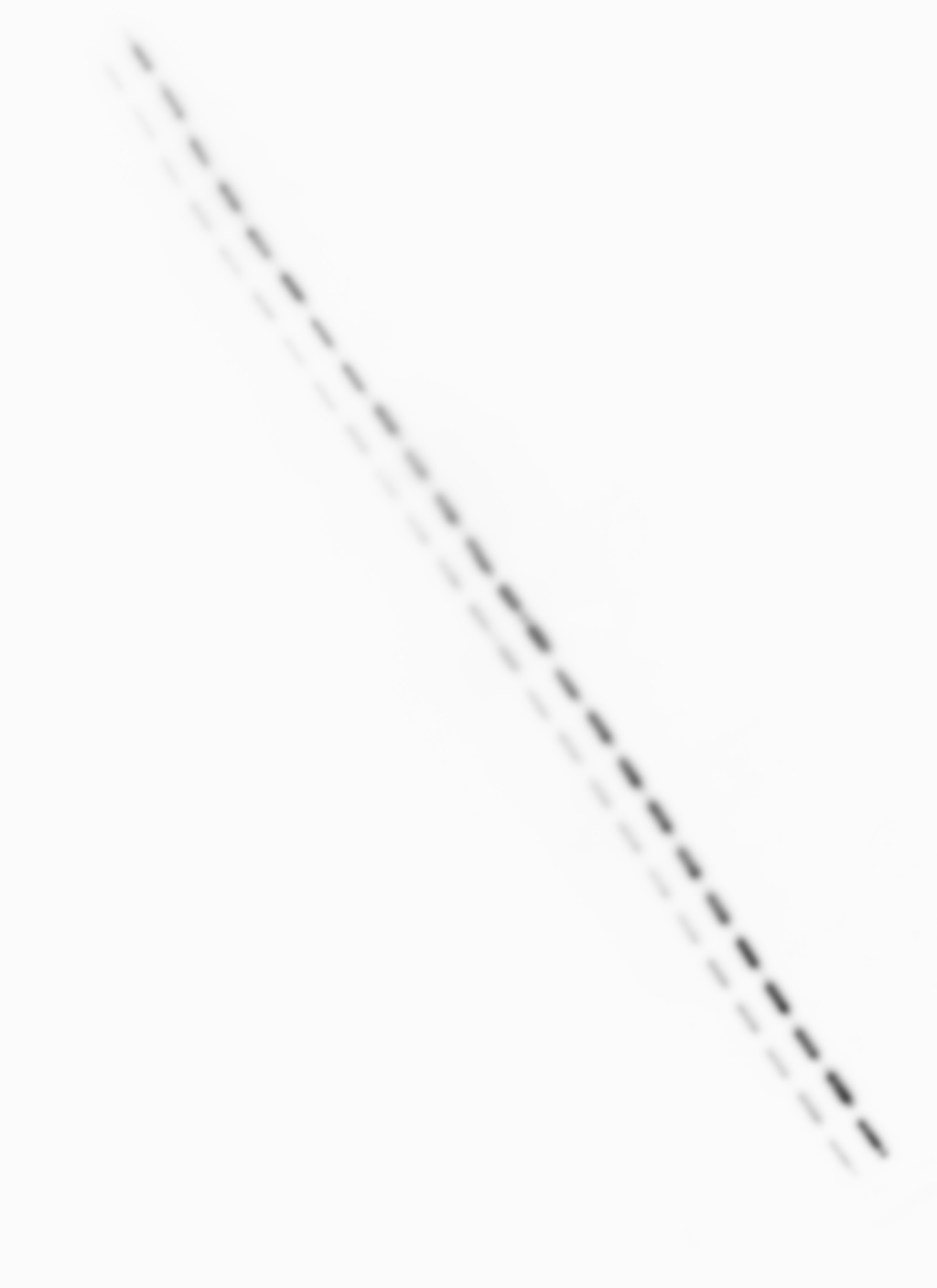

Supplement: Figure 1—source data 1. [file elife-88206-fig1-data1.zip › Figure 1 - source data/Figure 1 - source data 5/Western blot 5 - A6 to A10 - total EB2 - uncropped.tif]

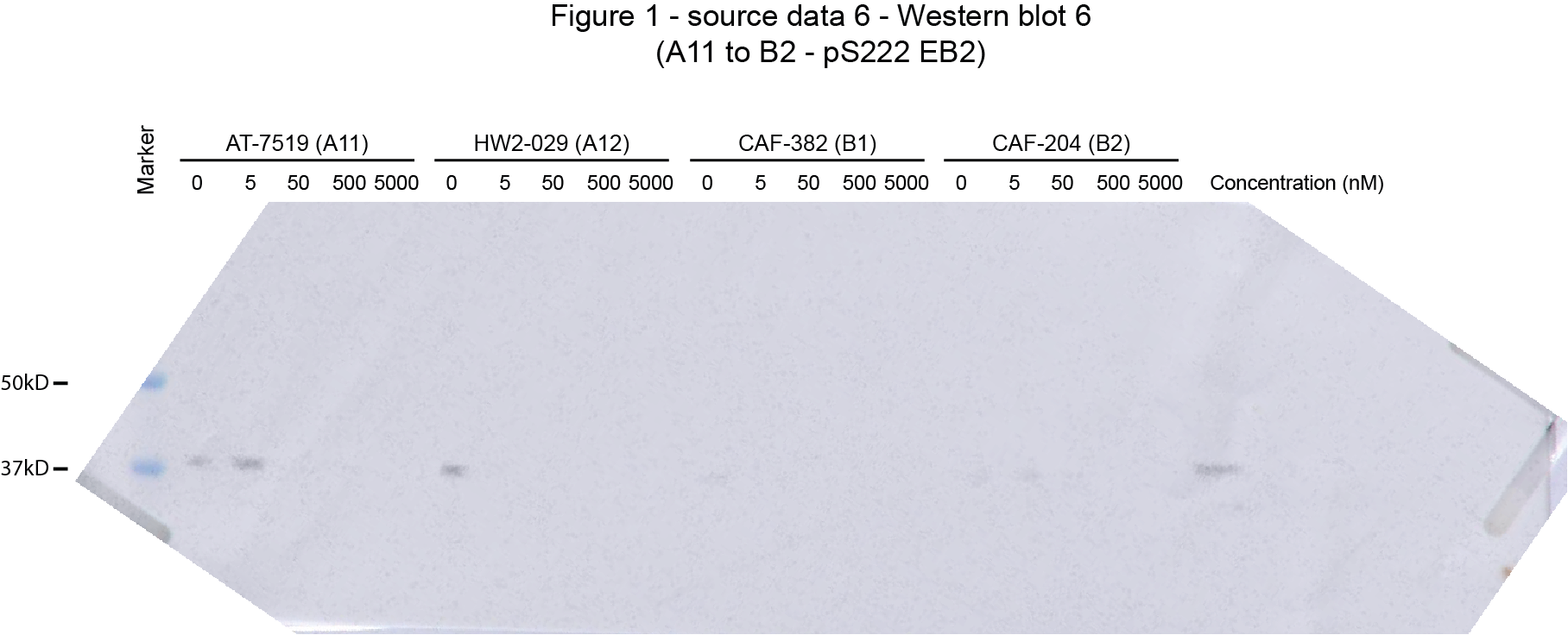

Supplement: Figure 1—source data 1. [file elife-88206-fig1-data1.zip › Figure 1 - source data/Figure 1 - source data 6/Western blot 6 - A11 to B2 - pEB2 - labeled.png]

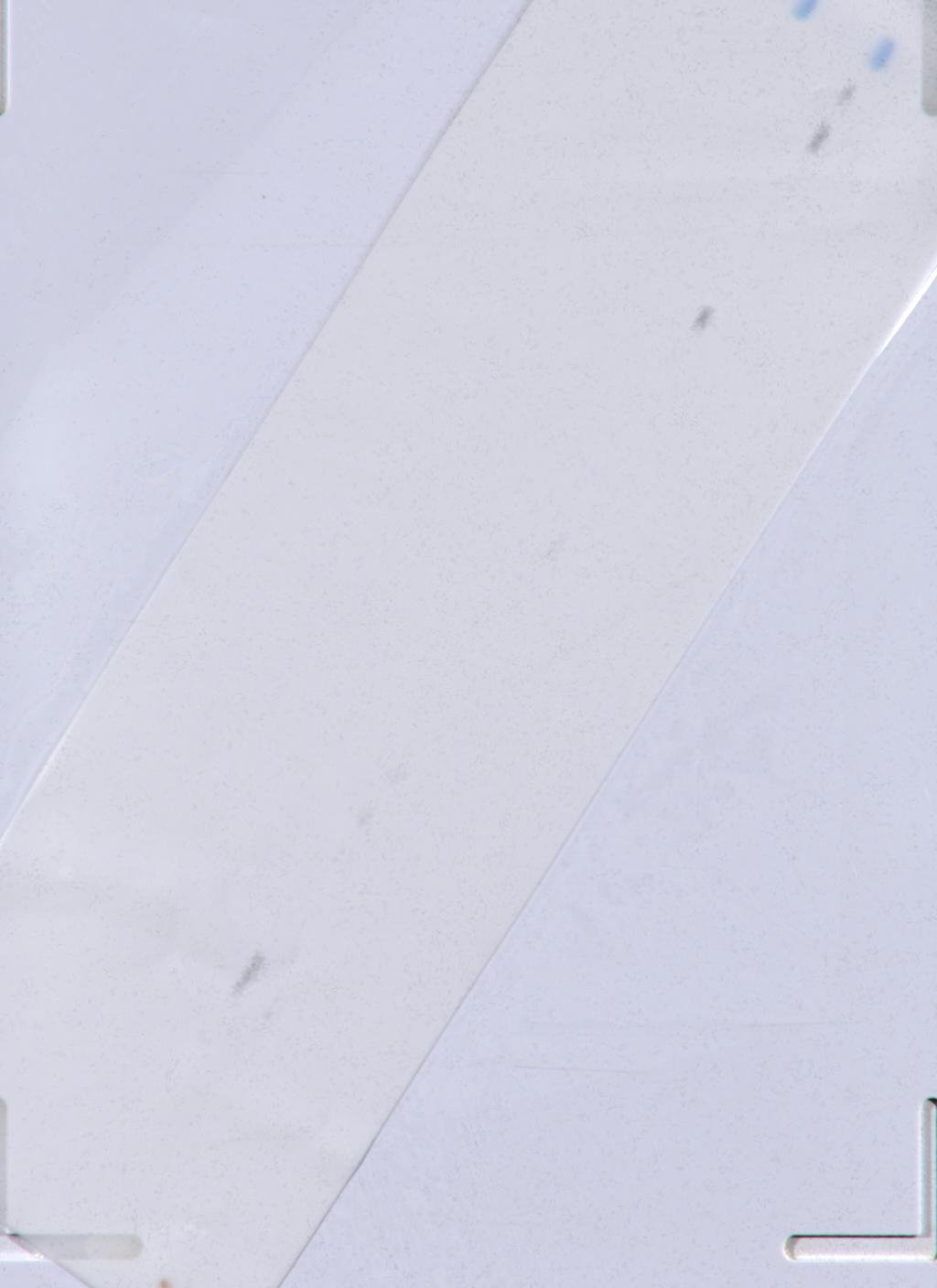

Supplement: Figure 1—source data 1. [file elife-88206-fig1-data1.zip › Figure 1 - source data/Figure 1 - source data 6/Western blot 6 - A11 to B2 - pEB2 - uncropped.jpg]

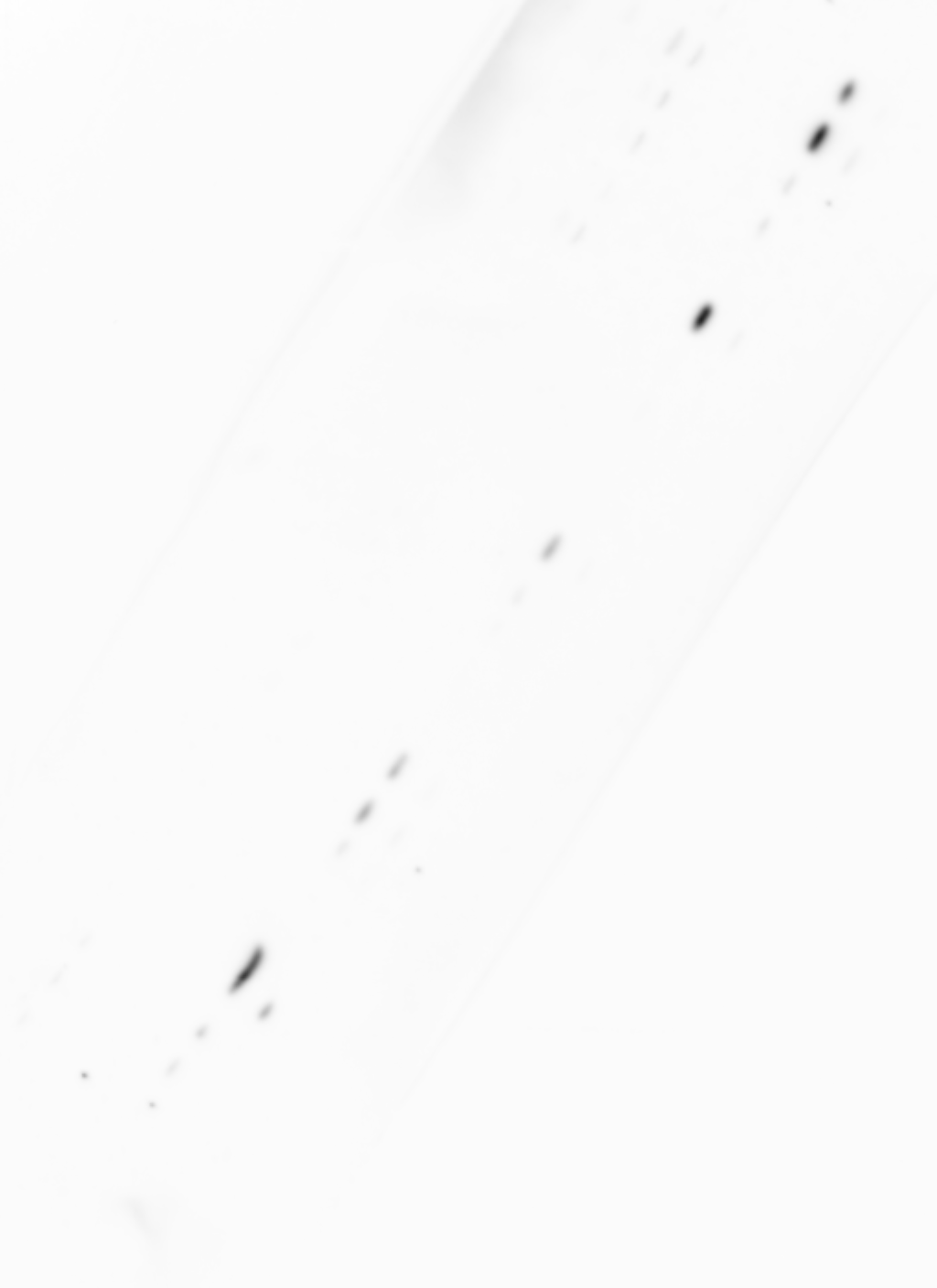

Supplement: Figure 1—source data 1. [file elife-88206-fig1-data1.zip › Figure 1 - source data/Figure 1 - source data 6/Western blot 6 - A11 to B2 - pEB2 - uncropped.tif]

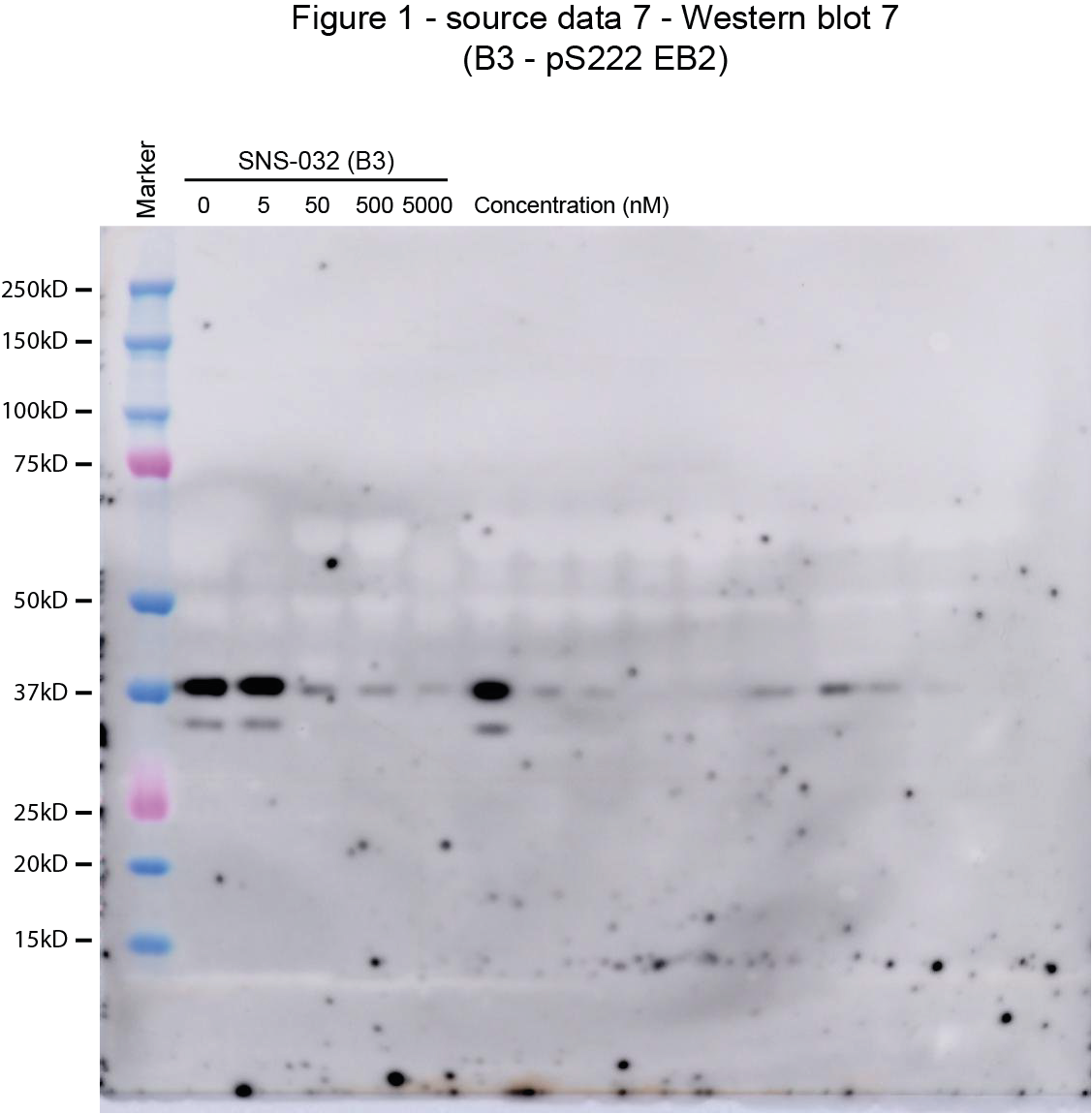

Supplement: Figure 1—source data 1. [file elife-88206-fig1-data1.zip › Figure 1 - source data/Figure 1 - source data 7/Western blot 7 - B3 - pEB2 - labeled.png]

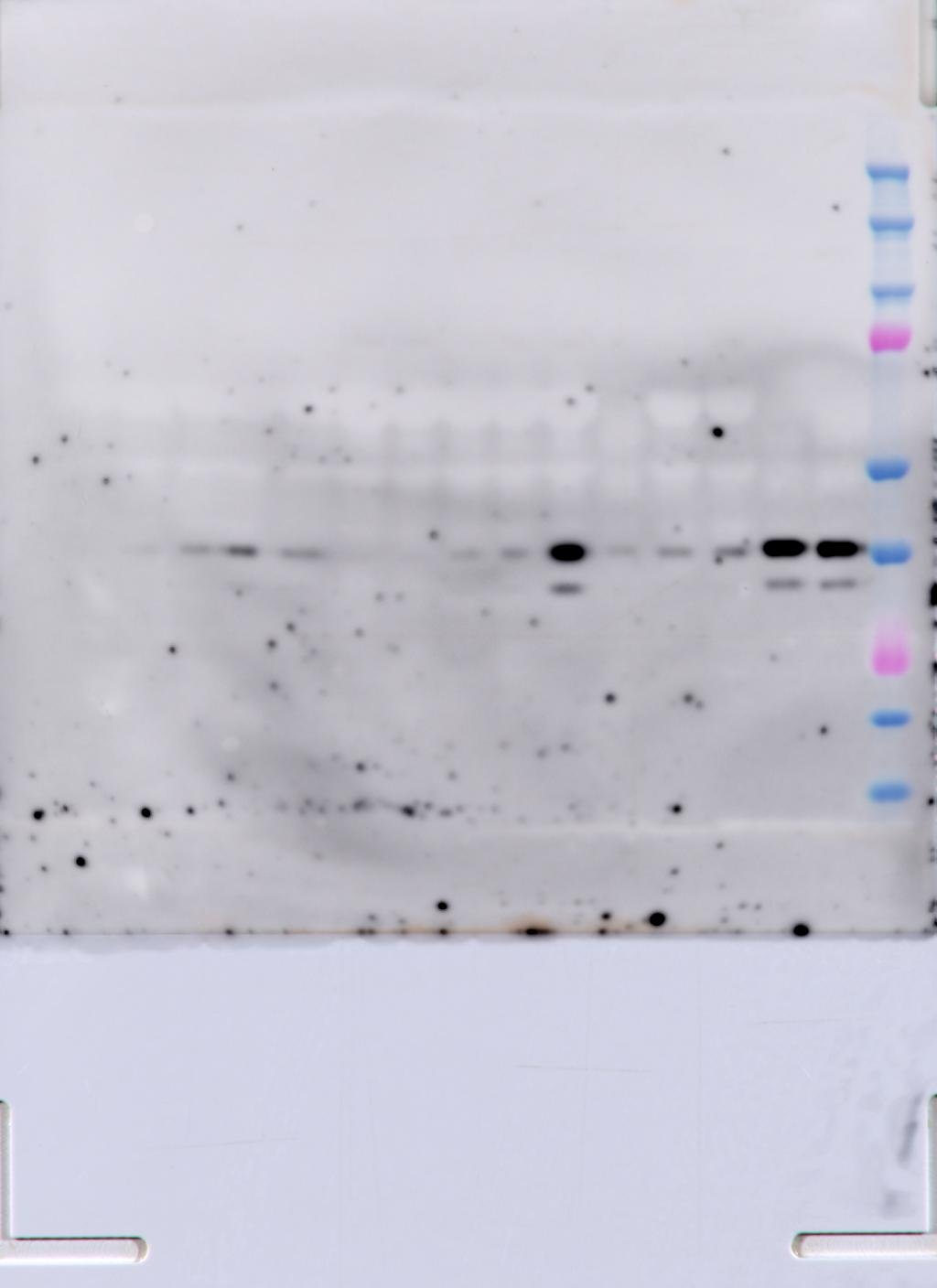

Supplement: Figure 1—source data 1. [file elife-88206-fig1-data1.zip › Figure 1 - source data/Figure 1 - source data 7/Western blot 7 - B3 - pEB2 - uncropped.jpg]

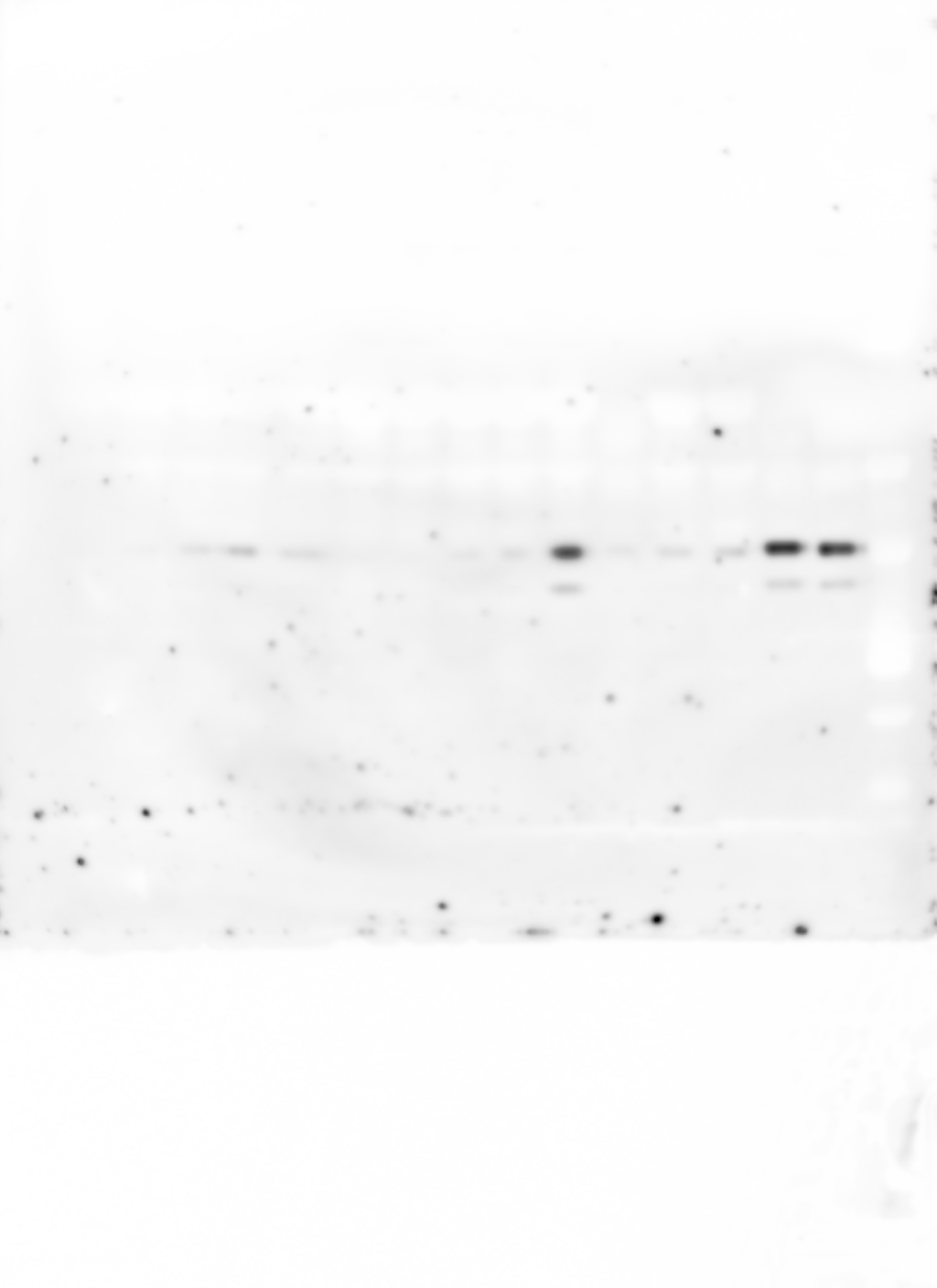

Supplement: Figure 1—source data 1. [file elife-88206-fig1-data1.zip › Figure 1 - source data/Figure 1 - source data 7/Western blot 7 - B3 - pEB2 - uncropped.tif]

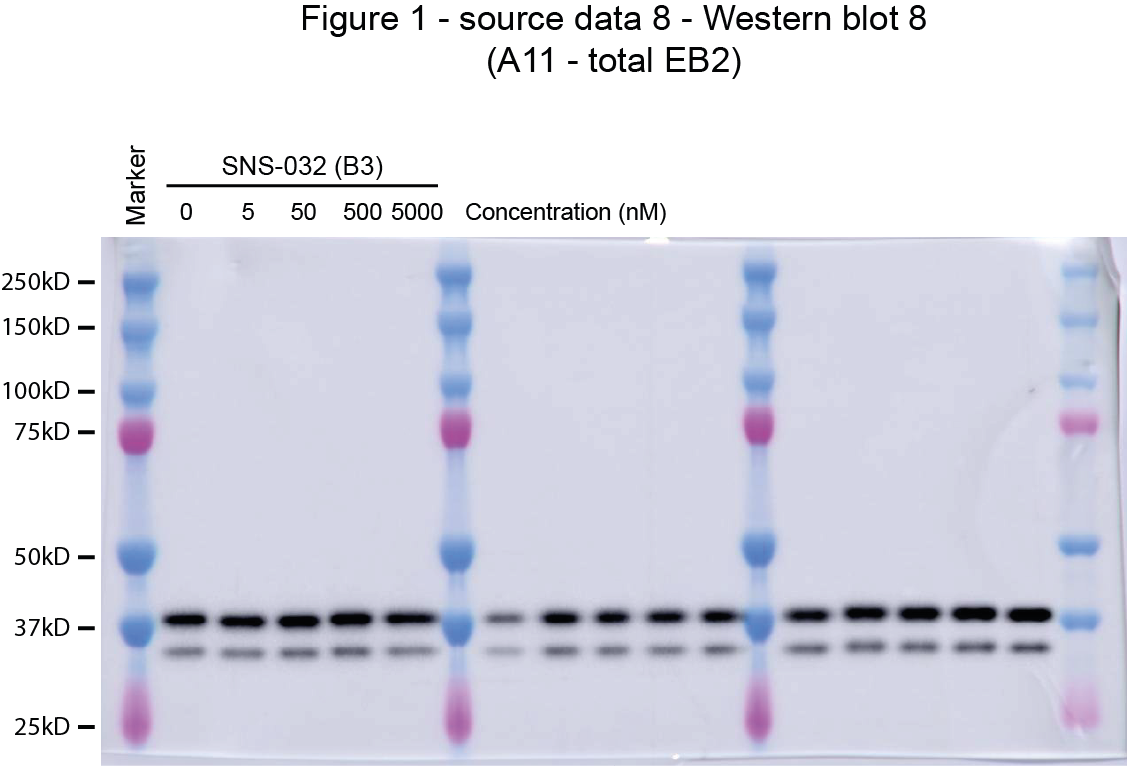

Supplement: Figure 1—source data 1. [file elife-88206-fig1-data1.zip › Figure 1 - source data/Figure 1 - source data 8/Western blot 8 - A11 - total EB2 - labeled.png]

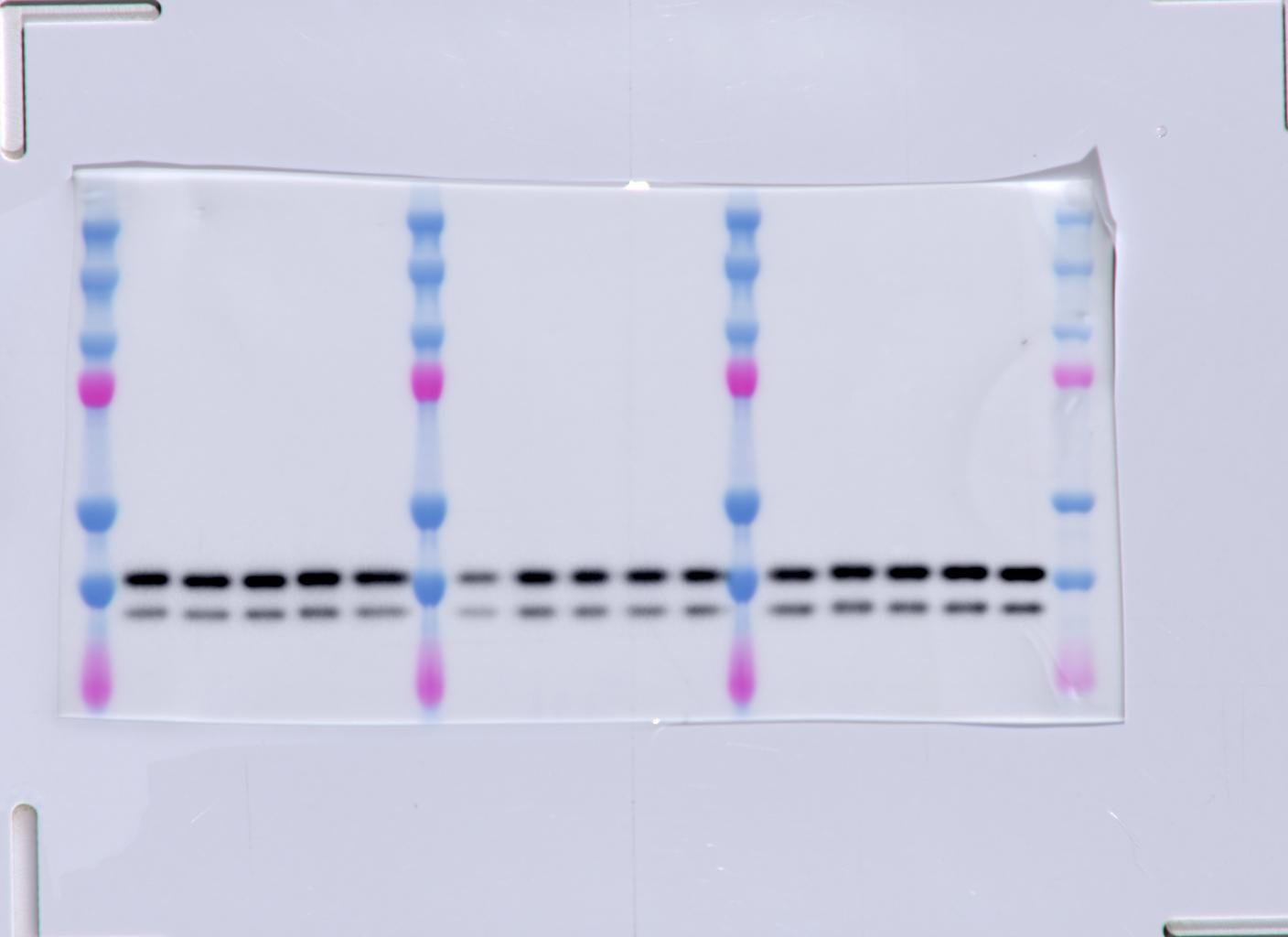

Supplement: Figure 1—source data 1. [file elife-88206-fig1-data1.zip › Figure 1 - source data/Figure 1 - source data 8/Western blot 8 - A11 - total EB2 - uncropped.jpg]

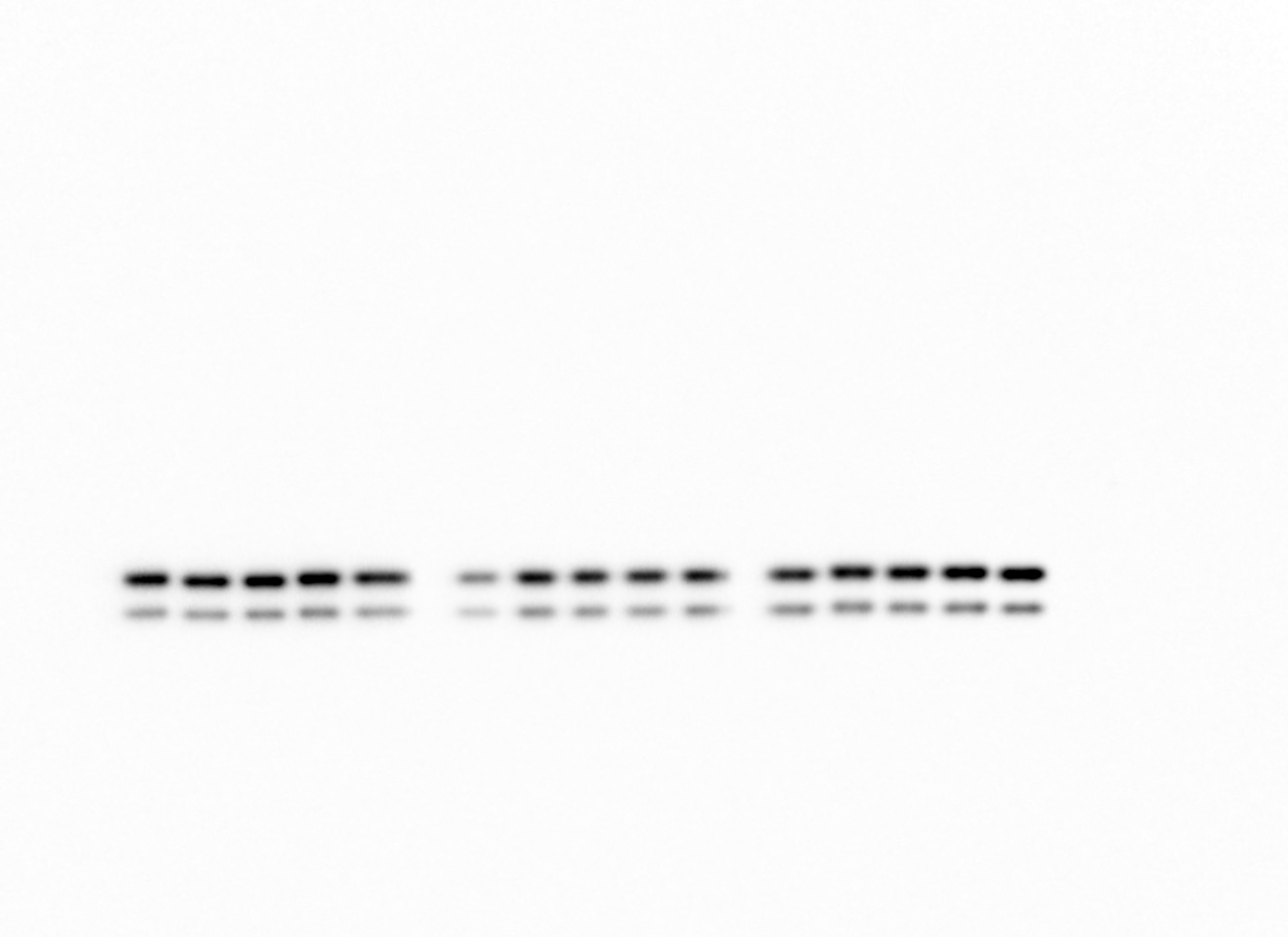

Supplement: Figure 1—source data 1. [file elife-88206-fig1-data1.zip › Figure 1 - source data/Figure 1 - source data 8/Western blot 8 - A11 - total EB2 - uncropped.tif]

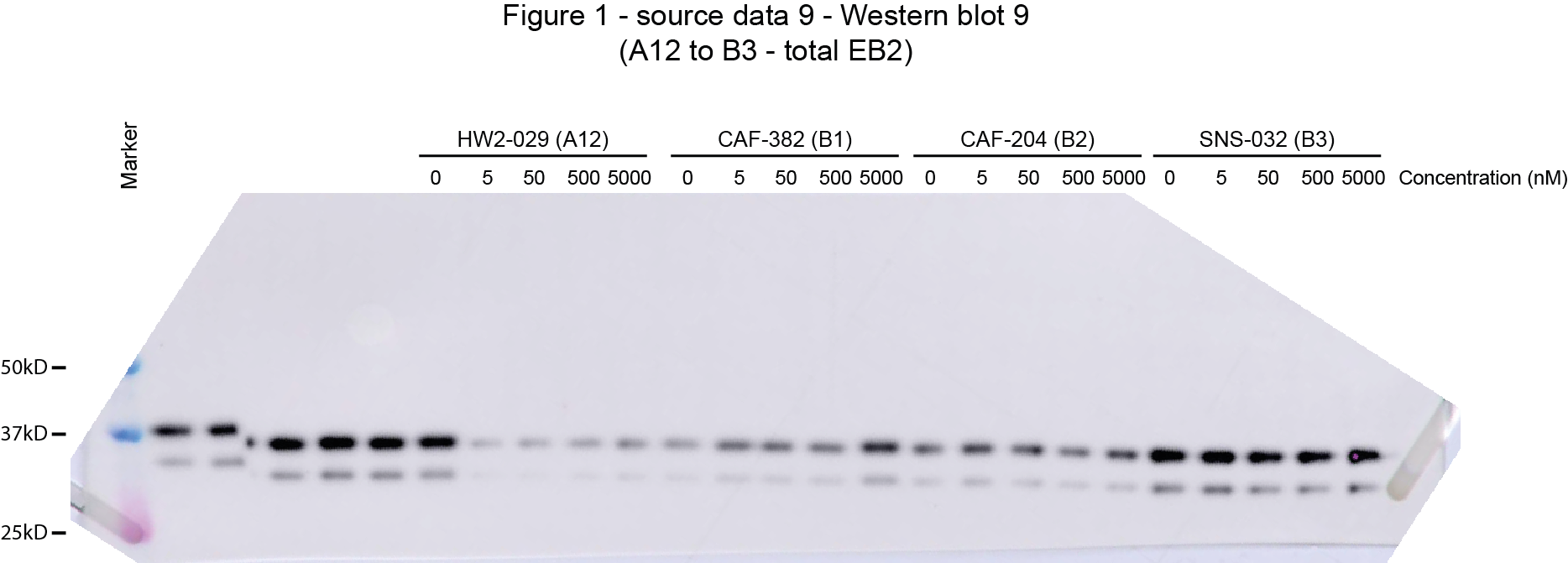

Supplement: Figure 1—source data 1. [file elife-88206-fig1-data1.zip › Figure 1 - source data/Figure 1 - source data 9/Western blot 9 - A12 to B3 - total EB2 - labeled.png]

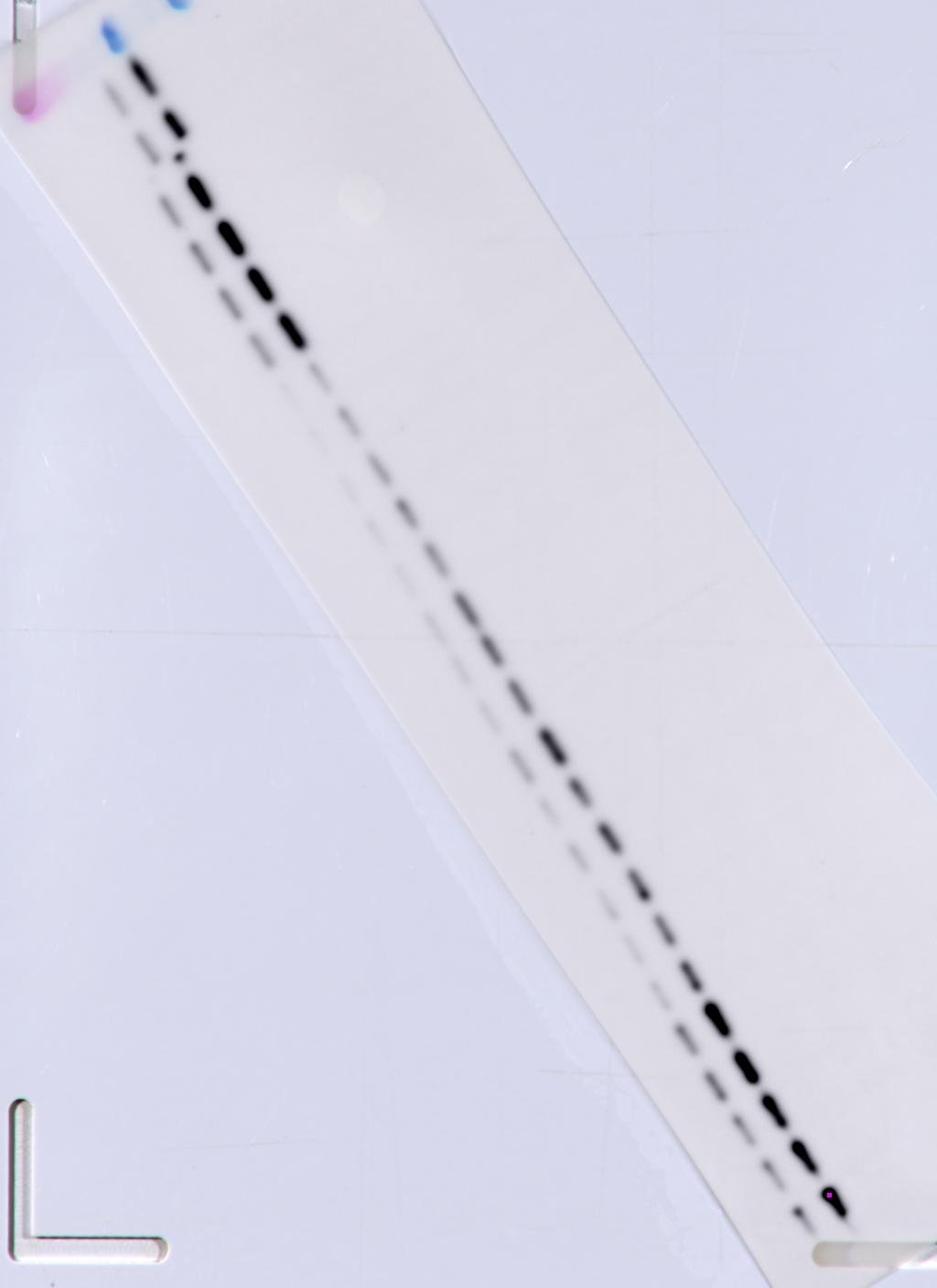

Supplement: Figure 1—source data 1. [file elife-88206-fig1-data1.zip › Figure 1 - source data/Figure 1 - source data 9/Western blot 9 - A12 to B3 - total EB2 - uncropped.jpg]

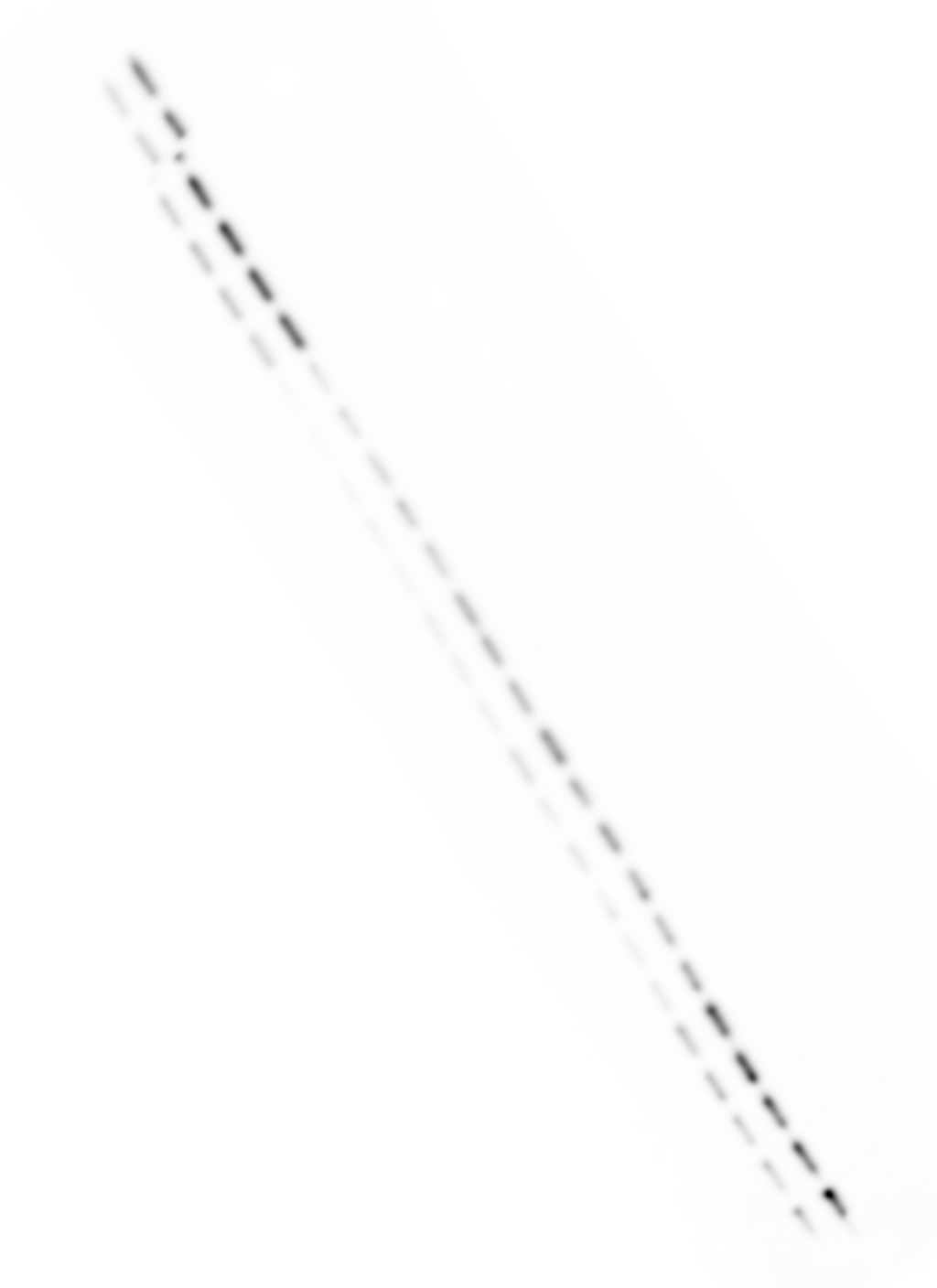

Supplement: Figure 1—source data 1. [file elife-88206-fig1-data1.zip › Figure 1 - source data/Figure 1 - source data 9/Western blot 9 - A12 to B3 - total EB2 - uncropped.tif]

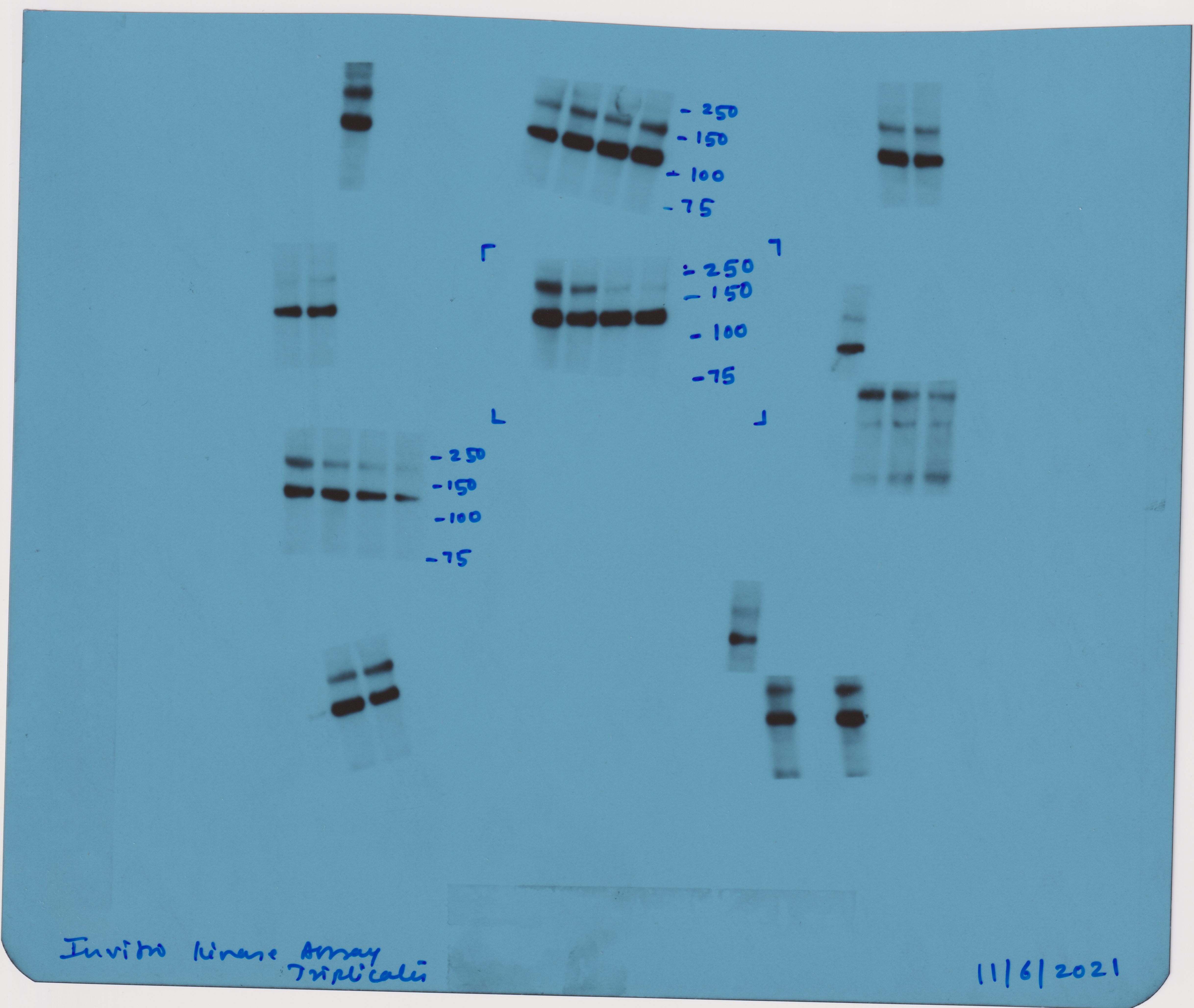

Supplement: Figure 4—source data 1. [file elife-88206-fig4-data1.zip › fig 4 source 1.tiff]

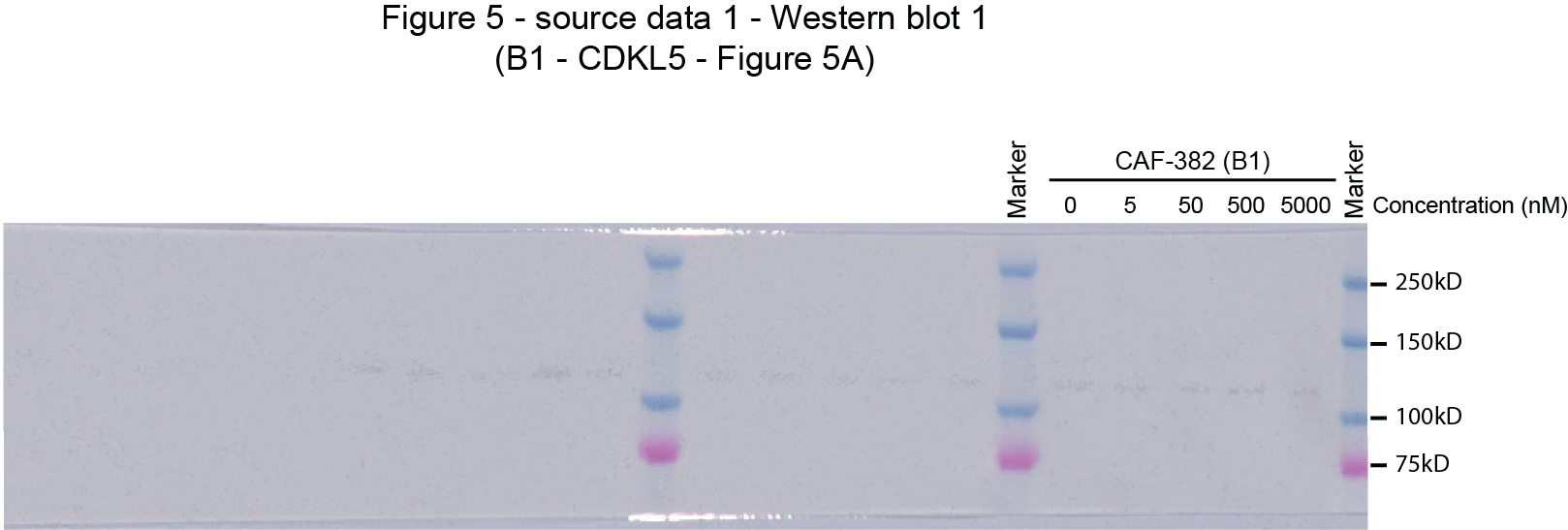

Supplement: Figure 5—source data 1. [file elife-88206-fig5-data1.zip › Figure 5 - source data/Figure 5 - source data 1/Western blot 1 - B1 - CDKL5 - labeled.png]

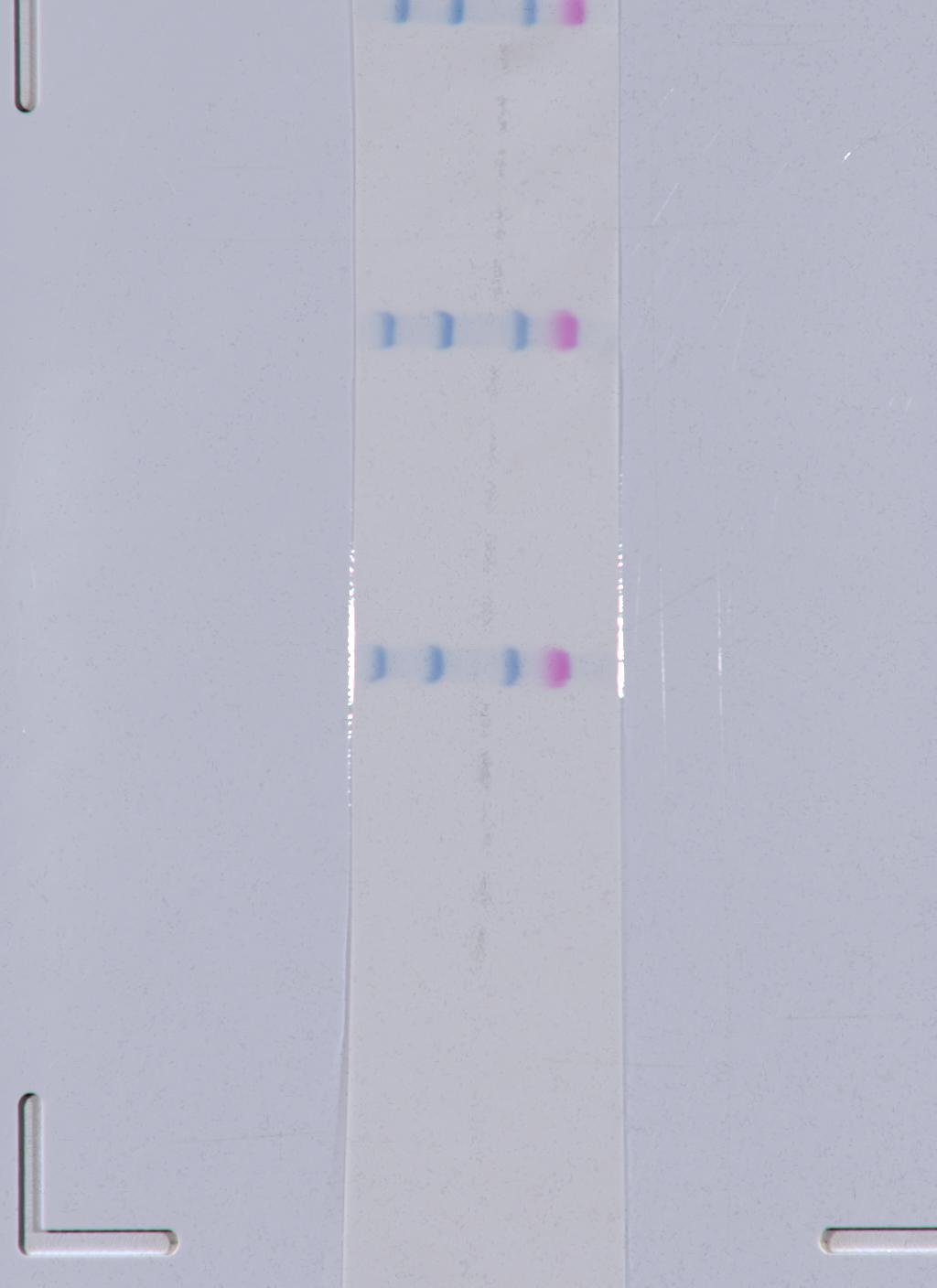

Supplement: Figure 5—source data 1. [file elife-88206-fig5-data1.zip › Figure 5 - source data/Figure 5 - source data 1/Western blot 1 - B1 - CDKL5 - uncropped.jpg]

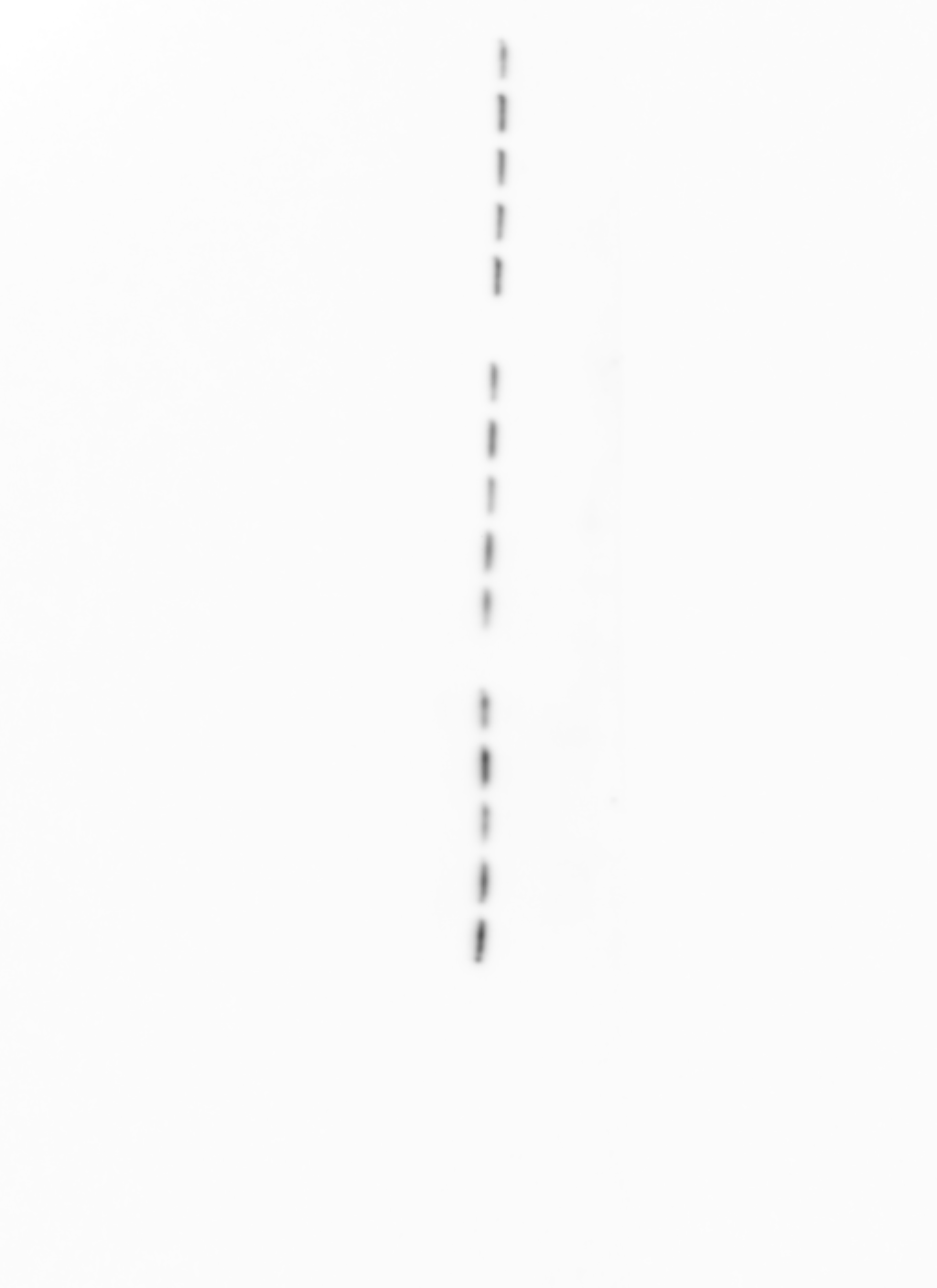

Supplement: Figure 5—source data 1. [file elife-88206-fig5-data1.zip › Figure 5 - source data/Figure 5 - source data 1/Western blot 1 - B1 - CDKL5 - uncropped.tif]

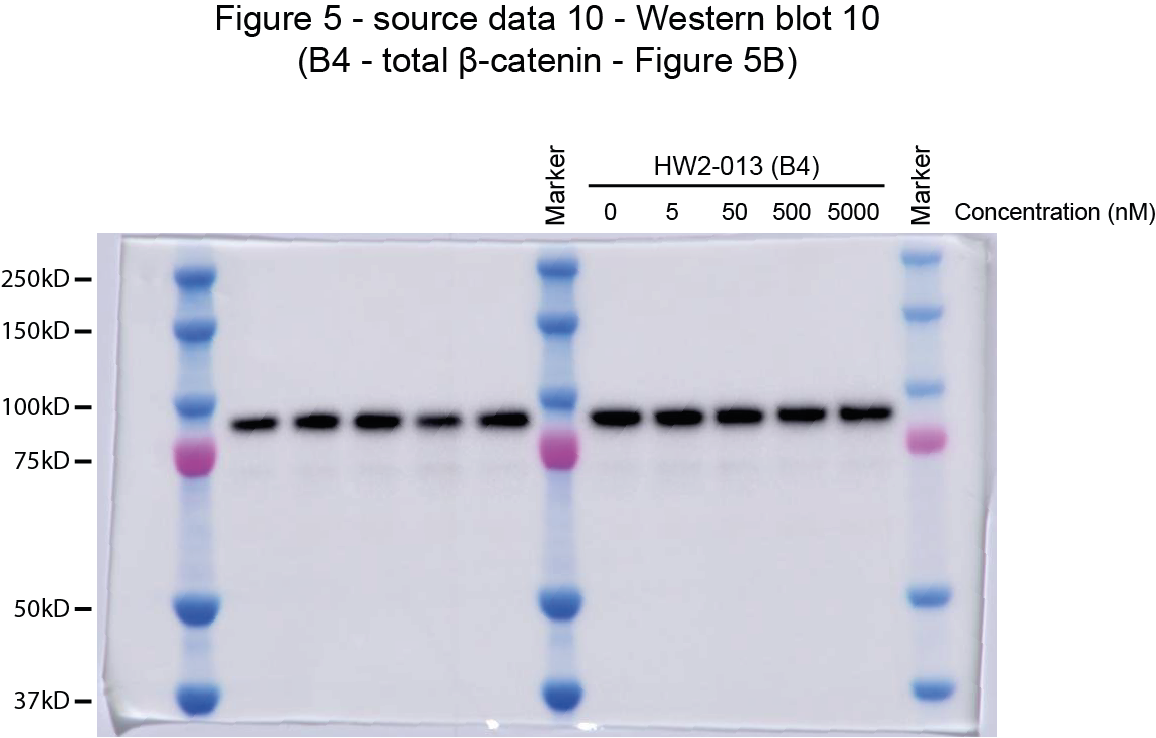

Supplement: Figure 5—source data 1. [file elife-88206-fig5-data1.zip › Figure 5 - source data/Figure 5 - source data 10/Western blot 10 - B4 - total bcatenin - labeled.png]

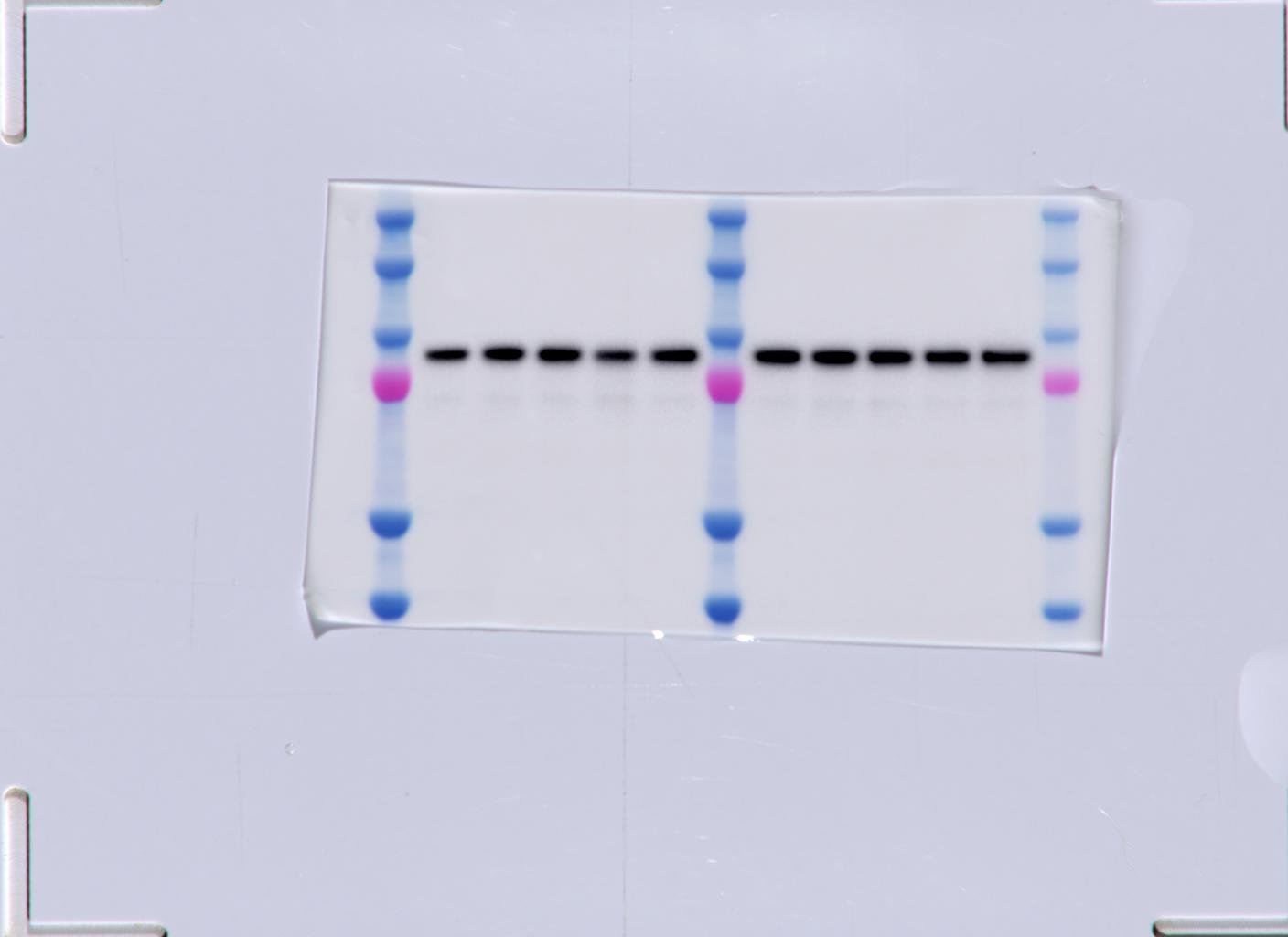

Supplement: Figure 5—source data 1. [file elife-88206-fig5-data1.zip › Figure 5 - source data/Figure 5 - source data 10/Western blot 10 - B4 - total bcatenin - uncropped.jpg]

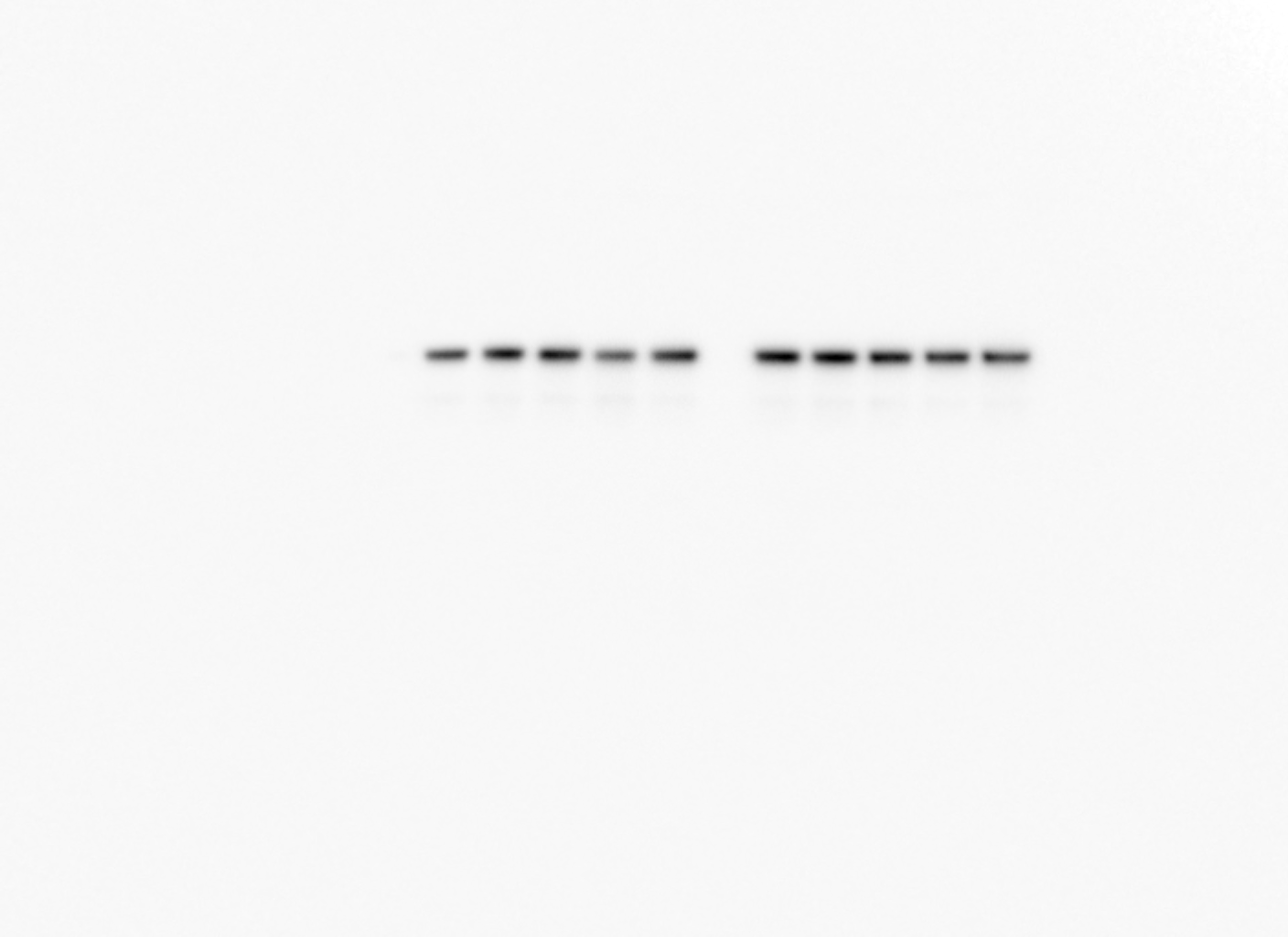

Supplement: Figure 5—source data 1. [file elife-88206-fig5-data1.zip › Figure 5 - source data/Figure 5 - source data 10/Western blot 10 - B4 - total bcatenin - uncropped.tif]

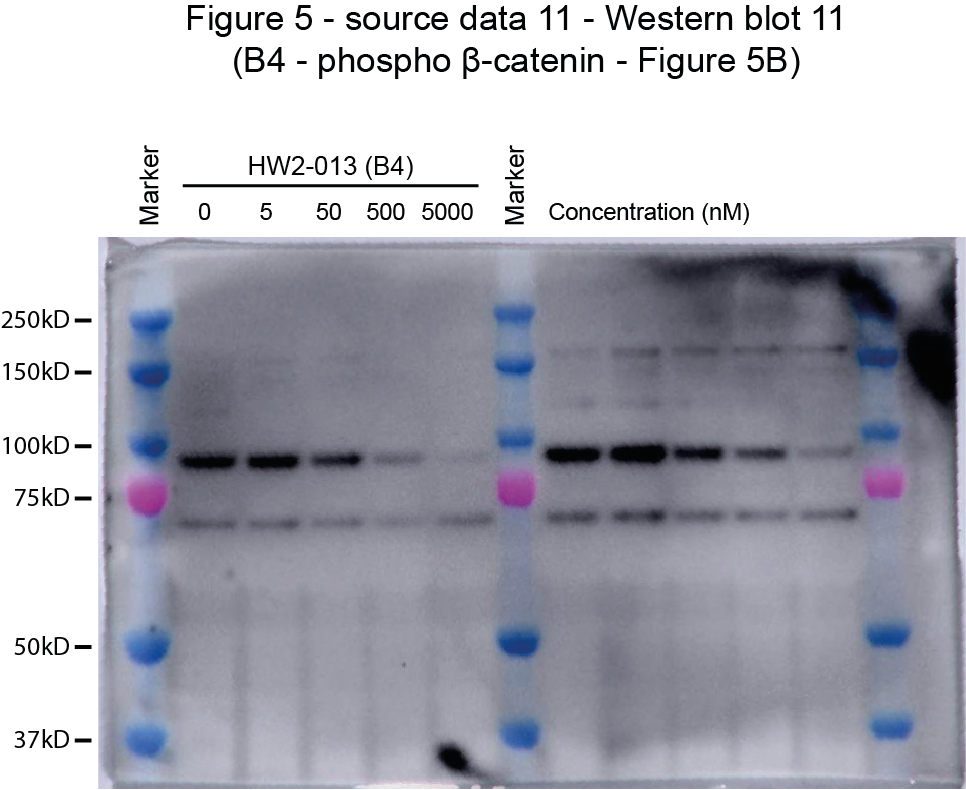

Supplement: Figure 5—source data 1. [file elife-88206-fig5-data1.zip › Figure 5 - source data/Figure 5 - source data 11/Western blot 11 - B4 - phospho bcatenin - labaled.png]

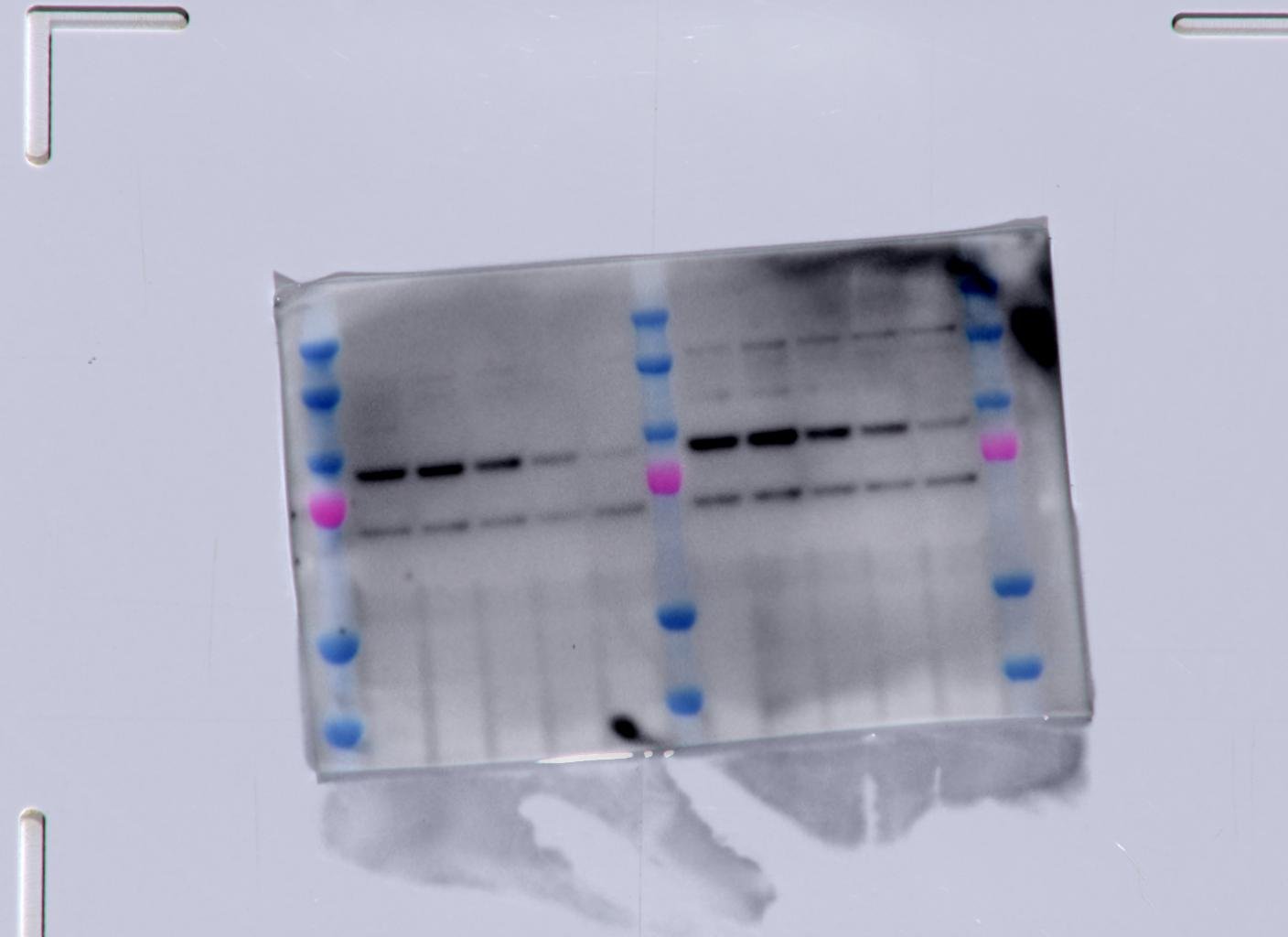

Supplement: Figure 5—source data 1. [file elife-88206-fig5-data1.zip › Figure 5 - source data/Figure 5 - source data 11/Western blot 11 - B4 - phospho bcatenin - uncropped.jpg]

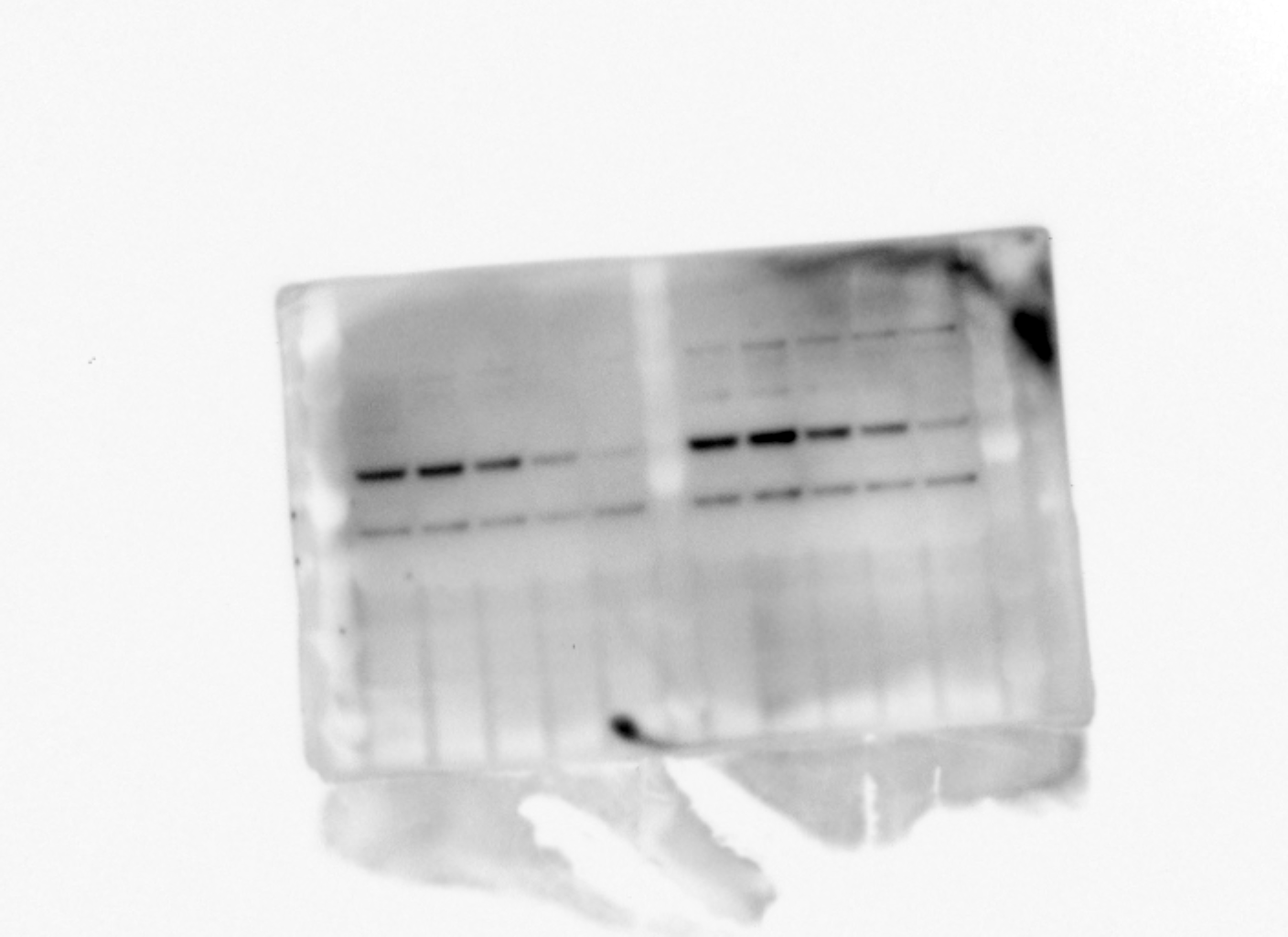

Supplement: Figure 5—source data 1. [file elife-88206-fig5-data1.zip › Figure 5 - source data/Figure 5 - source data 11/Western blot 11 - B4 - phospho bcatenin - uncropped.tif]

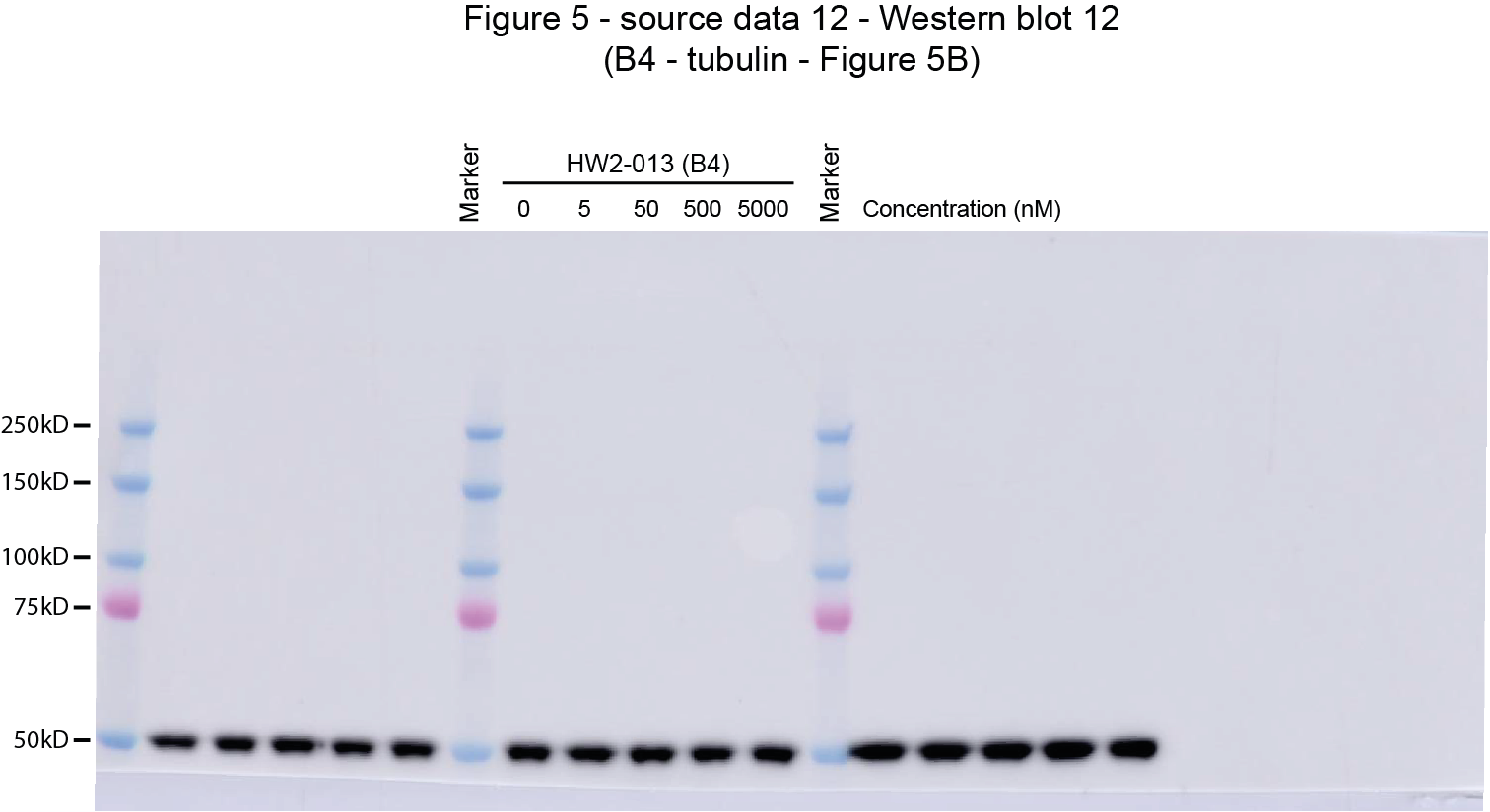

Supplement: Figure 5—source data 1. [file elife-88206-fig5-data1.zip › Figure 5 - source data/Figure 5 - source data 12/Western blot 12 - B4 - tubulin - labeled.png]

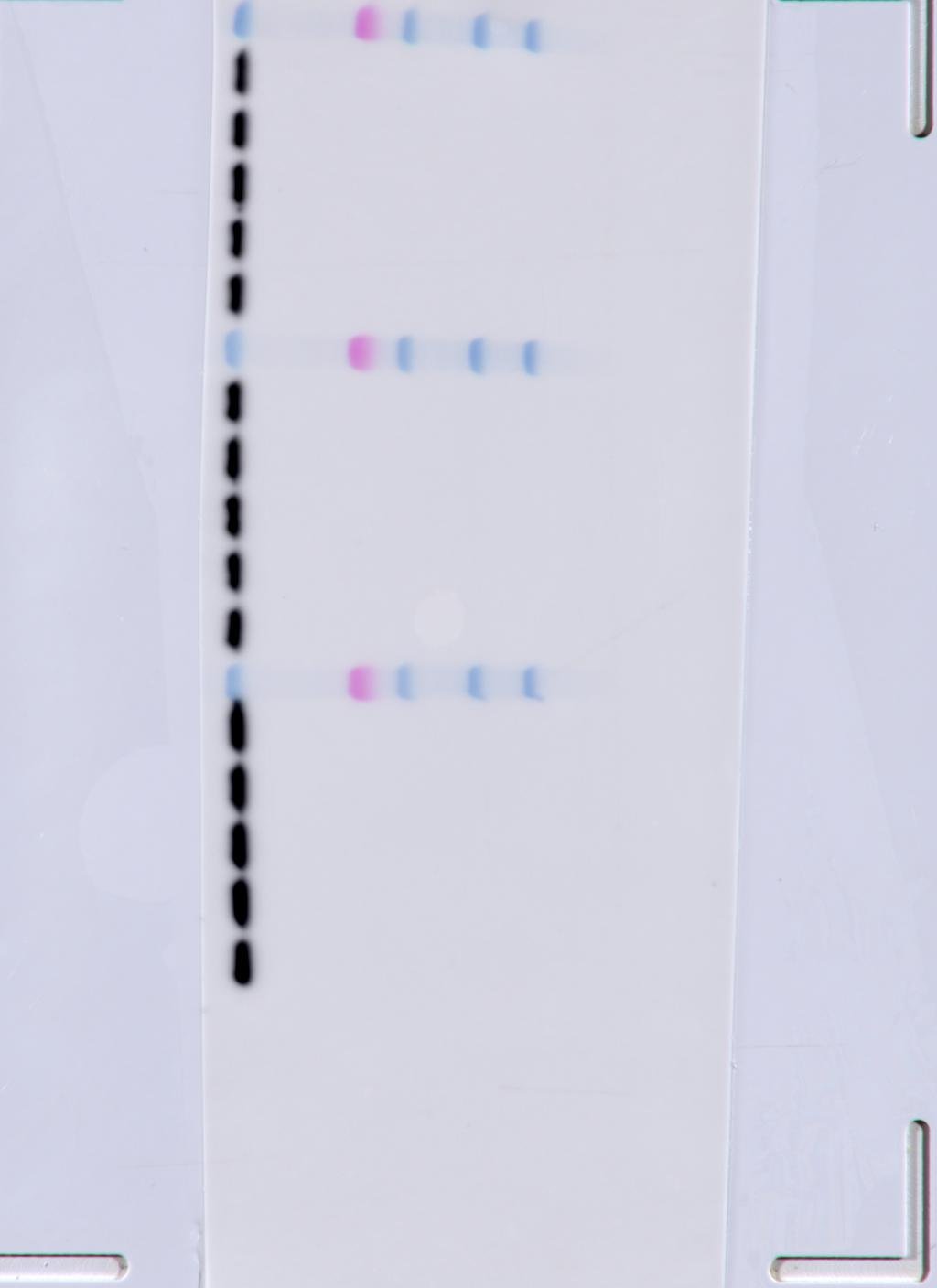

Supplement: Figure 5—source data 1. [file elife-88206-fig5-data1.zip › Figure 5 - source data/Figure 5 - source data 12/Western blot 12 - B4 - tubulin - uncropped.jpg]

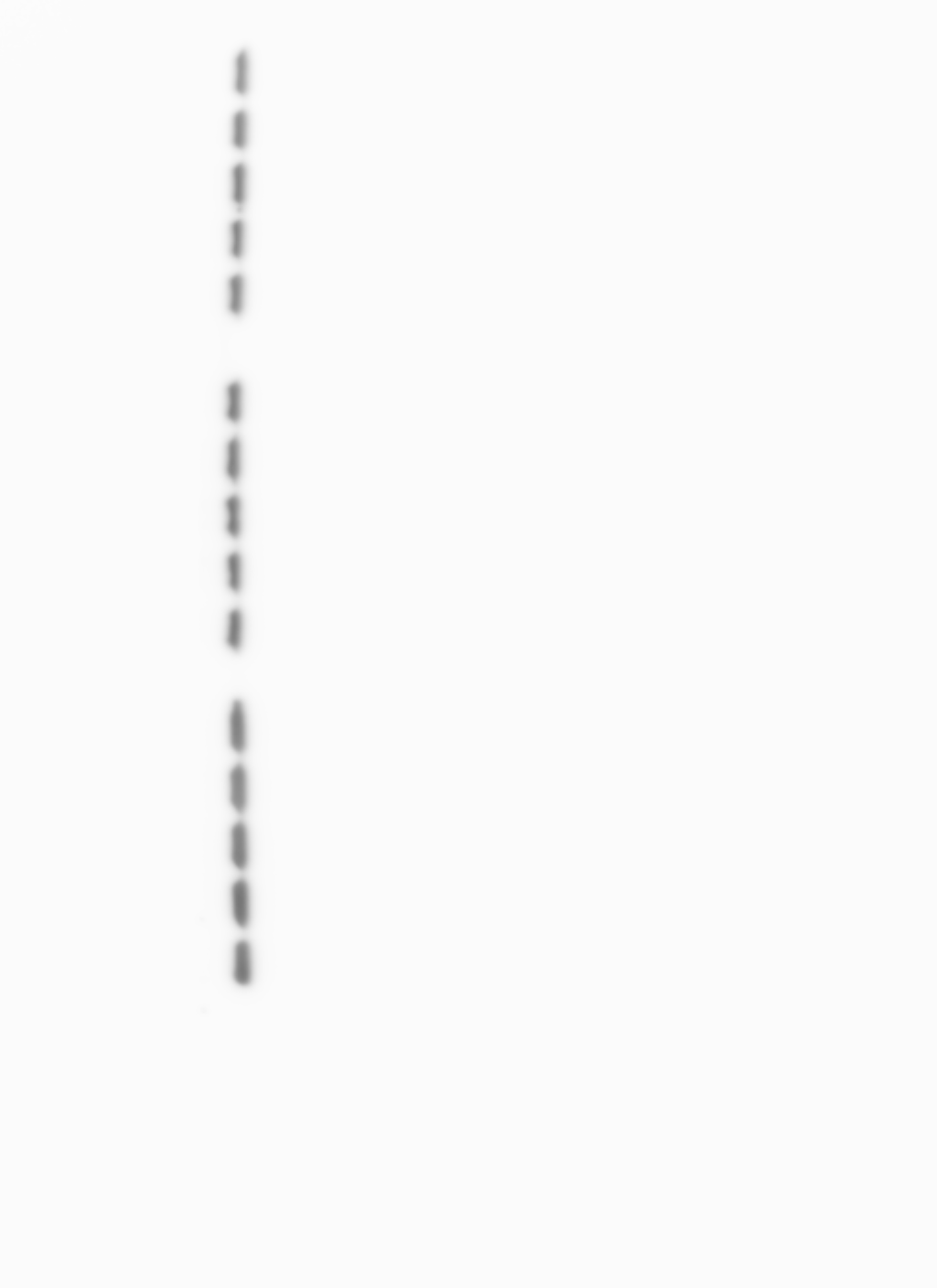

Supplement: Figure 5—source data 1. [file elife-88206-fig5-data1.zip › Figure 5 - source data/Figure 5 - source data 12/Western blot 12 - B4 - tubulin - uncropped.tif]

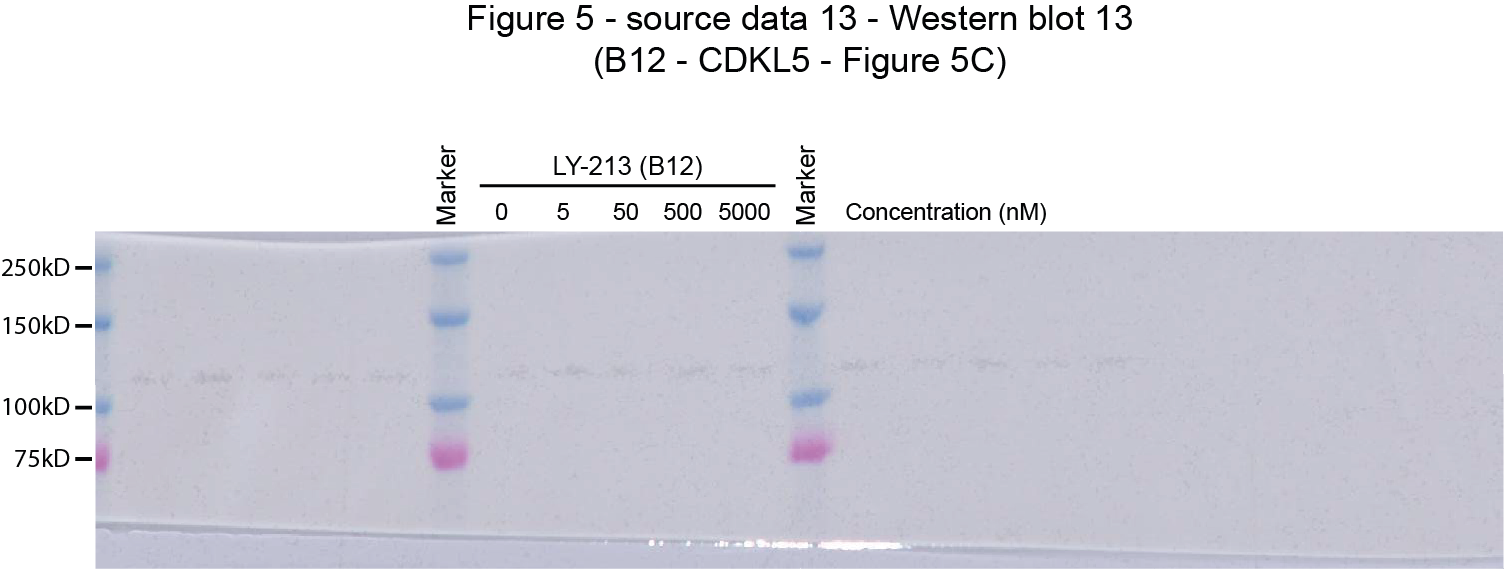

Supplement: Figure 5—source data 1. [file elife-88206-fig5-data1.zip › Figure 5 - source data/Figure 5 - source data 13/Western blot 13 - B12 - CDKL5 - labeled.png]

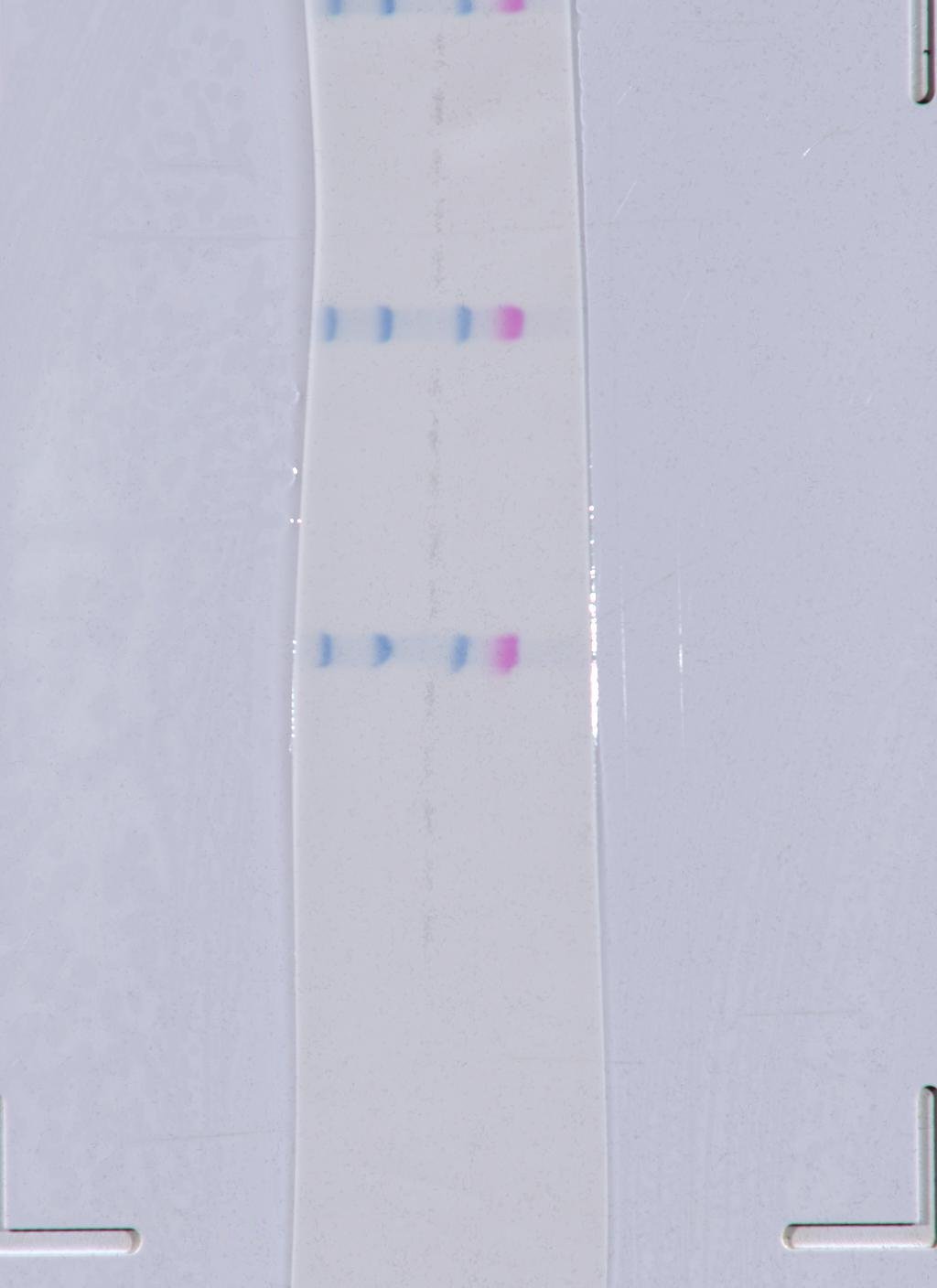

Supplement: Figure 5—source data 1. [file elife-88206-fig5-data1.zip › Figure 5 - source data/Figure 5 - source data 13/Western blot 13 - B12 - CDKL5 - uncropped.jpg]

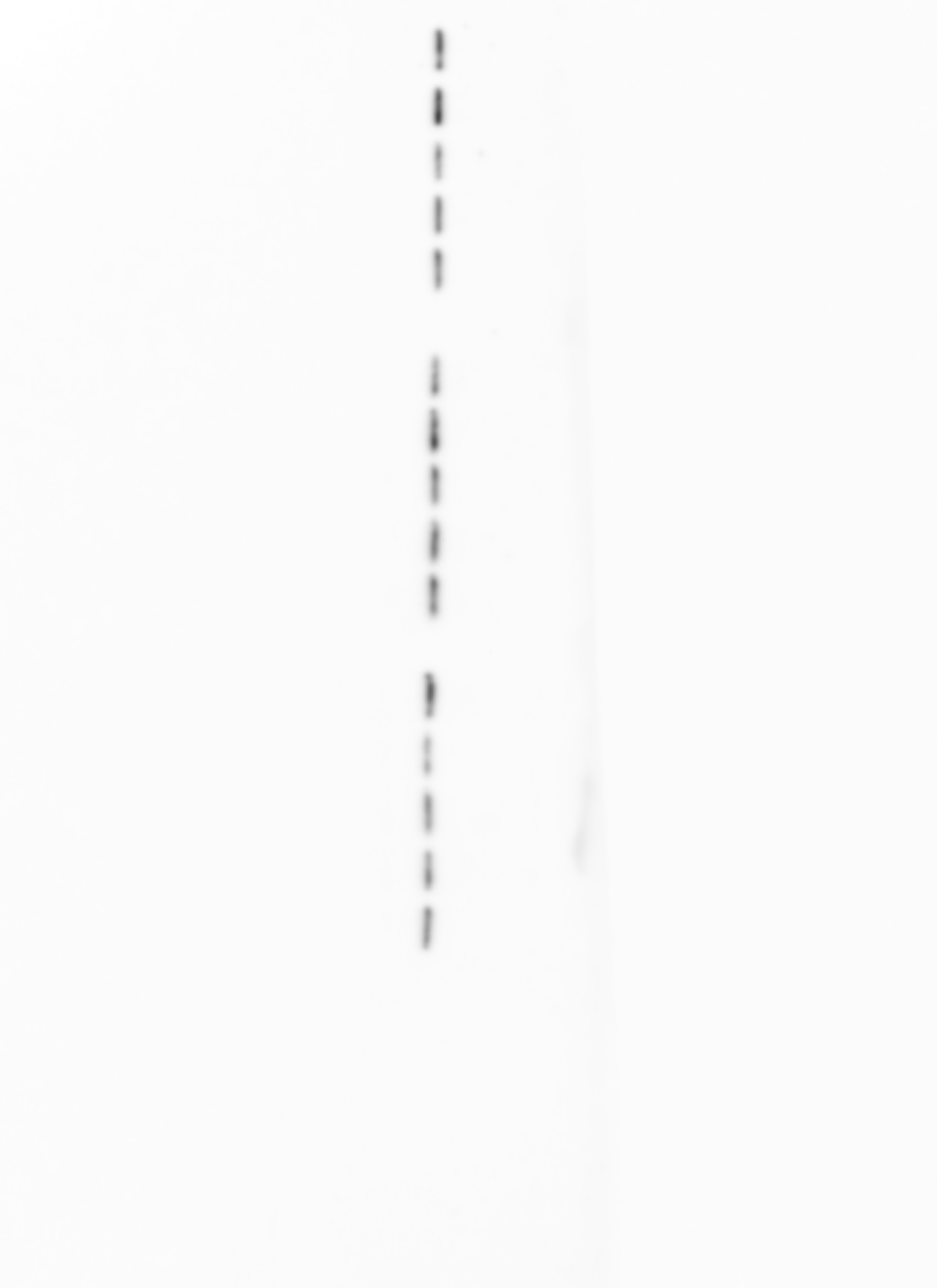

Supplement: Figure 5—source data 1. [file elife-88206-fig5-data1.zip › Figure 5 - source data/Figure 5 - source data 13/Western blot 13 - B12 - CDKL5 - uncropped.tif]

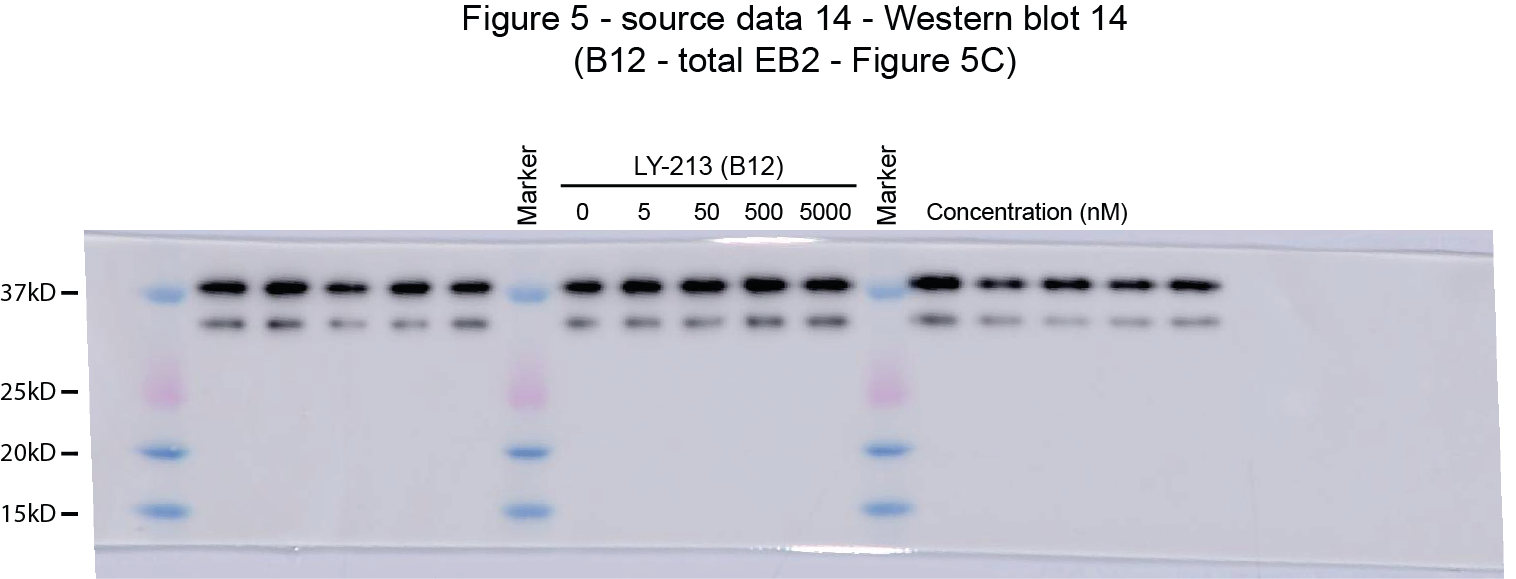

Supplement: Figure 5—source data 1. [file elife-88206-fig5-data1.zip › Figure 5 - source data/Figure 5 - source data 14/Western blot 14 - B12 - EB2 - labeled.png]

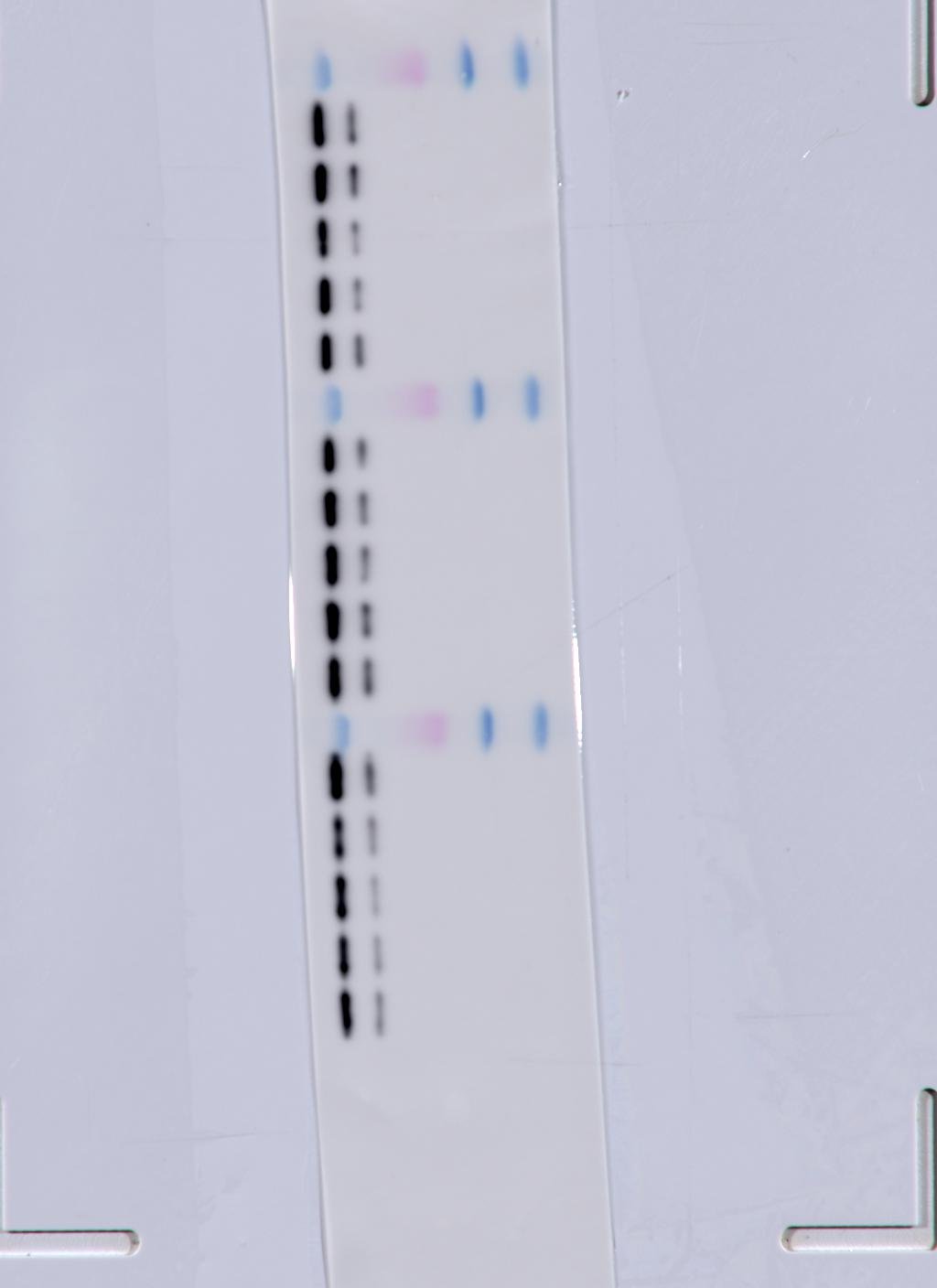

Supplement: Figure 5—source data 1. [file elife-88206-fig5-data1.zip › Figure 5 - source data/Figure 5 - source data 14/Western blot 14 - B12 - EB2 - uncropped.jpg]

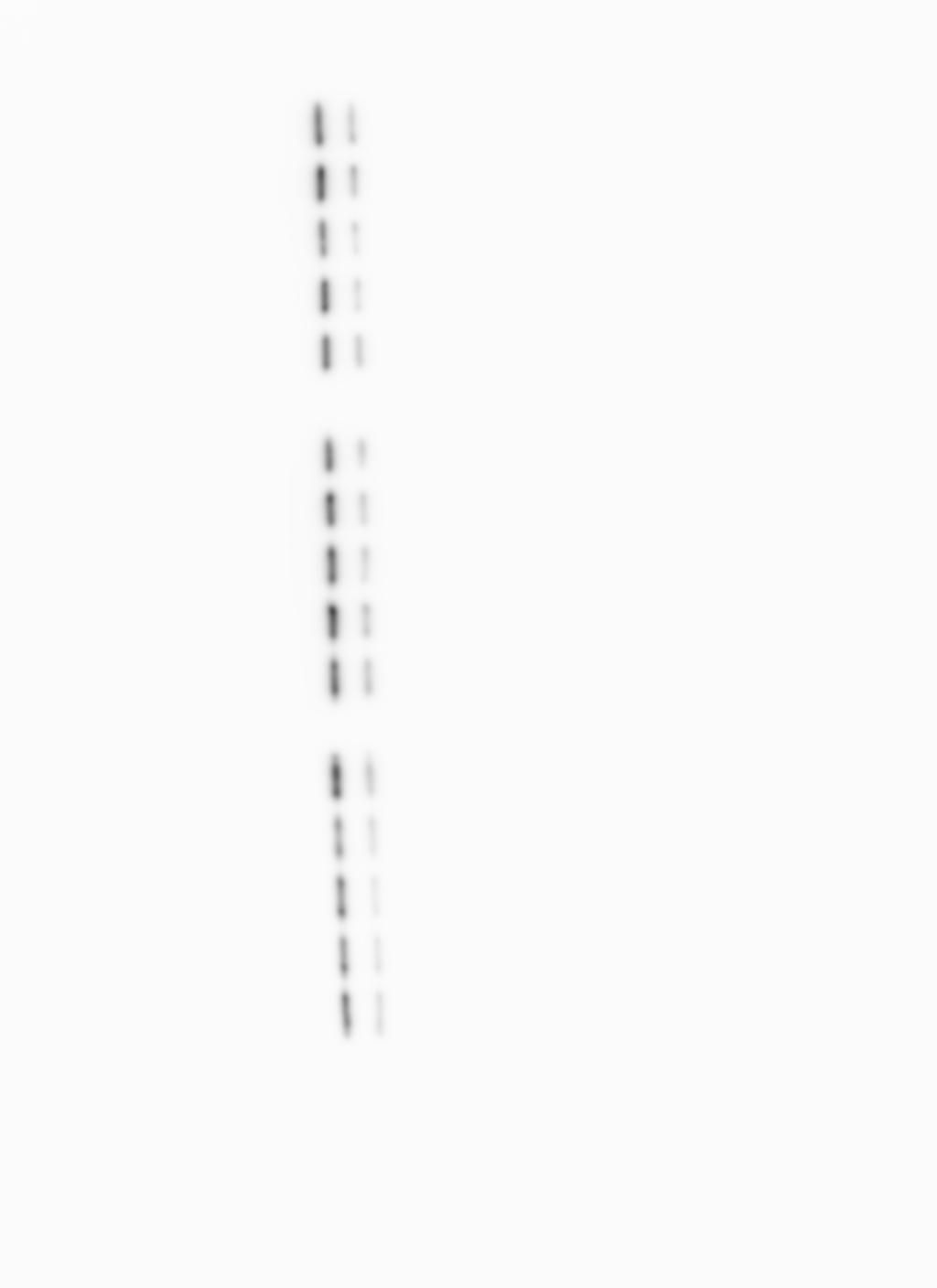

Supplement: Figure 5—source data 1. [file elife-88206-fig5-data1.zip › Figure 5 - source data/Figure 5 - source data 14/Western blot 14 - B12 - EB2 - uncropped.tif]

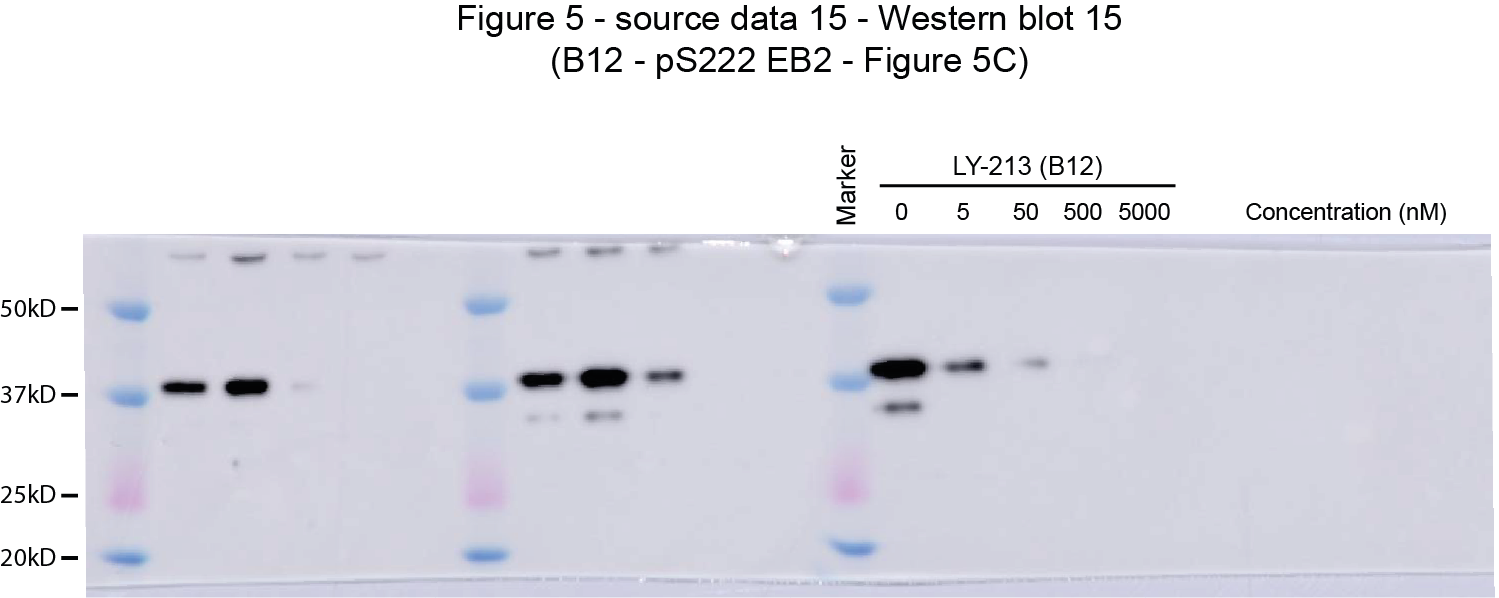

Supplement: Figure 5—source data 1. [file elife-88206-fig5-data1.zip › Figure 5 - source data/Figure 5 - source data 15/Western blot 15 - B12 - pEB2 - labeled.png]

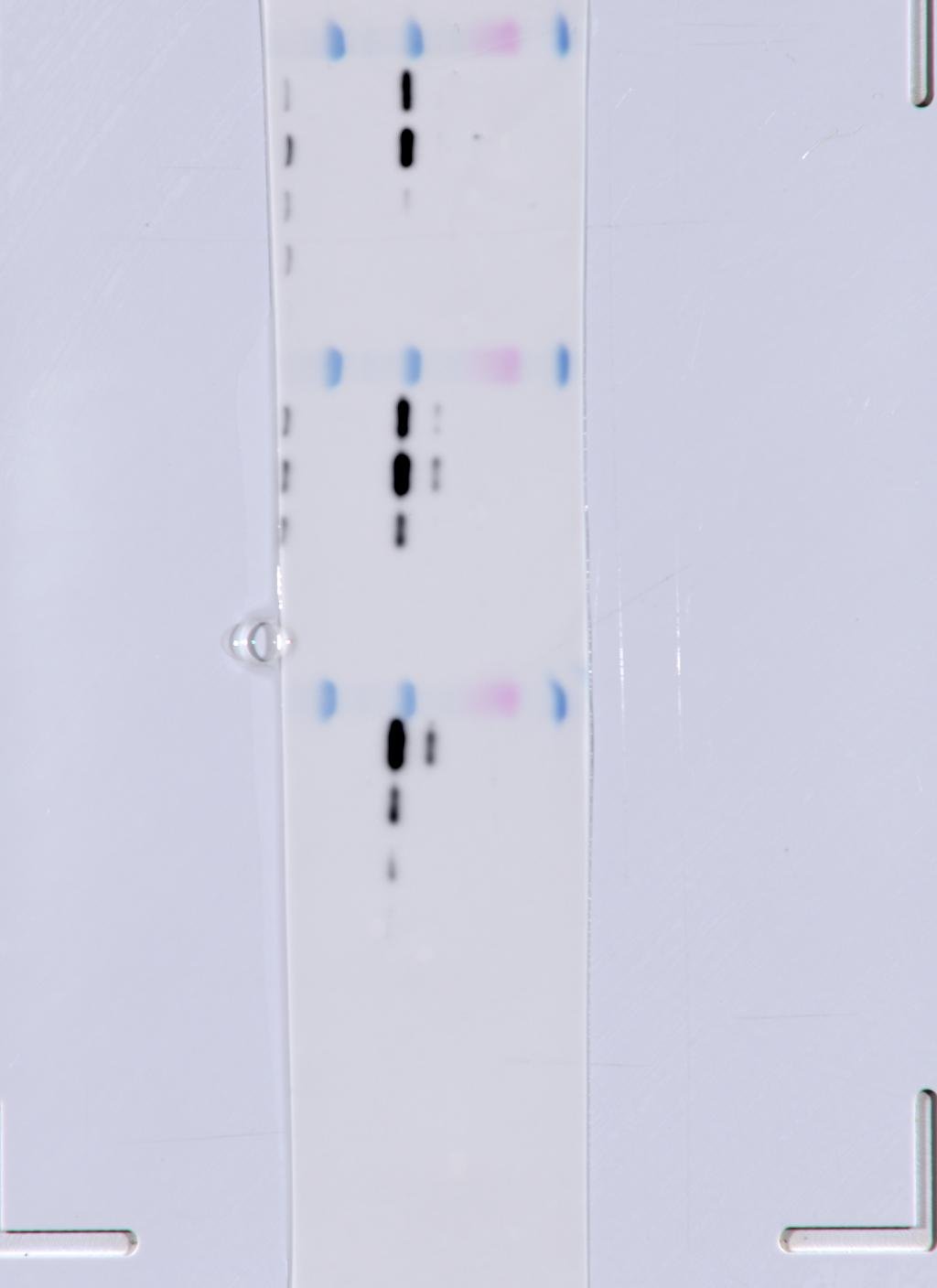

Supplement: Figure 5—source data 1. [file elife-88206-fig5-data1.zip › Figure 5 - source data/Figure 5 - source data 15/Western blot 15 - B12 - pEB2 - uncropped.jpg]

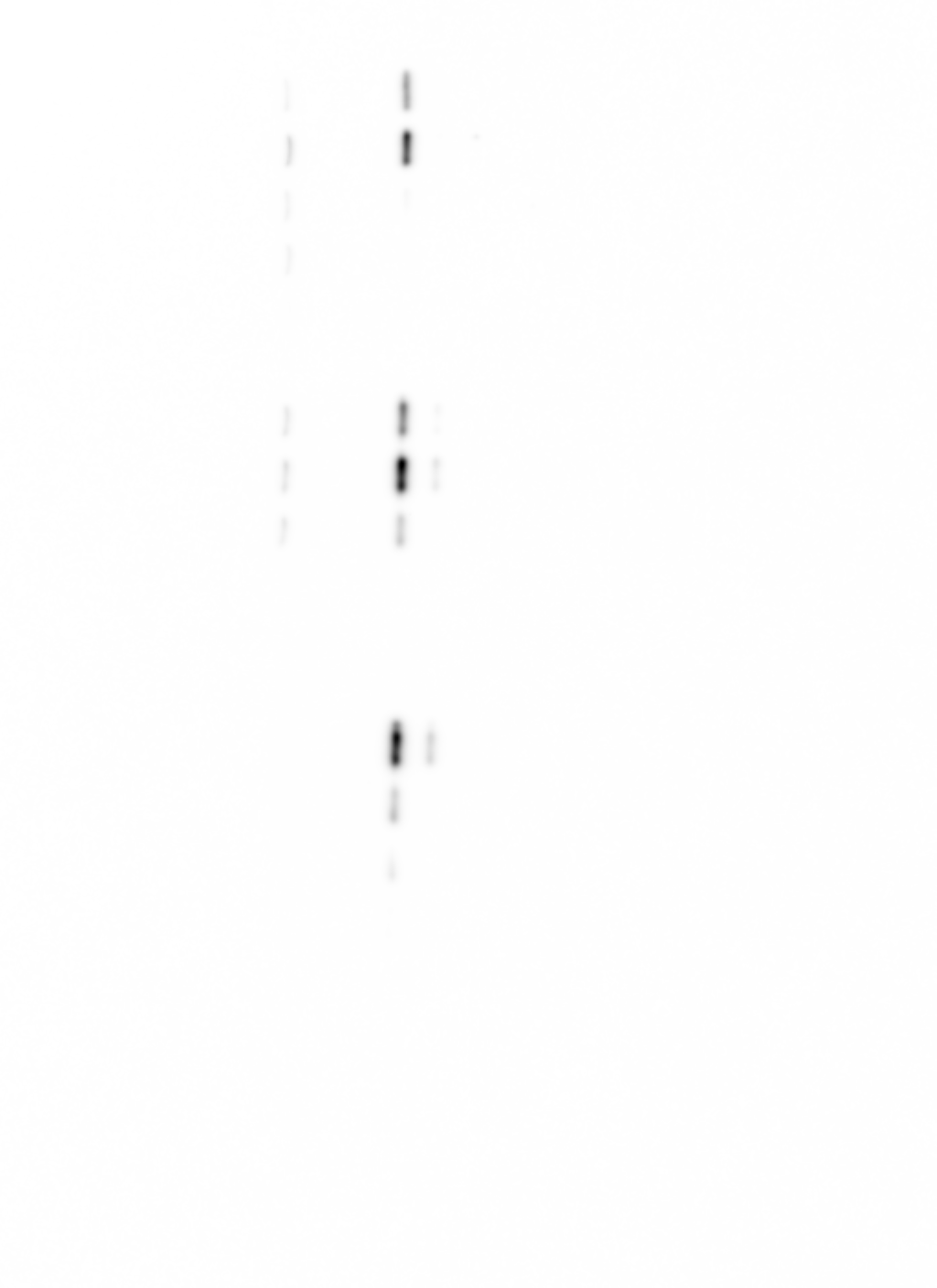

Supplement: Figure 5—source data 1. [file elife-88206-fig5-data1.zip › Figure 5 - source data/Figure 5 - source data 15/Western blot 15 - B12 - pEB2 - uncropped.tif]

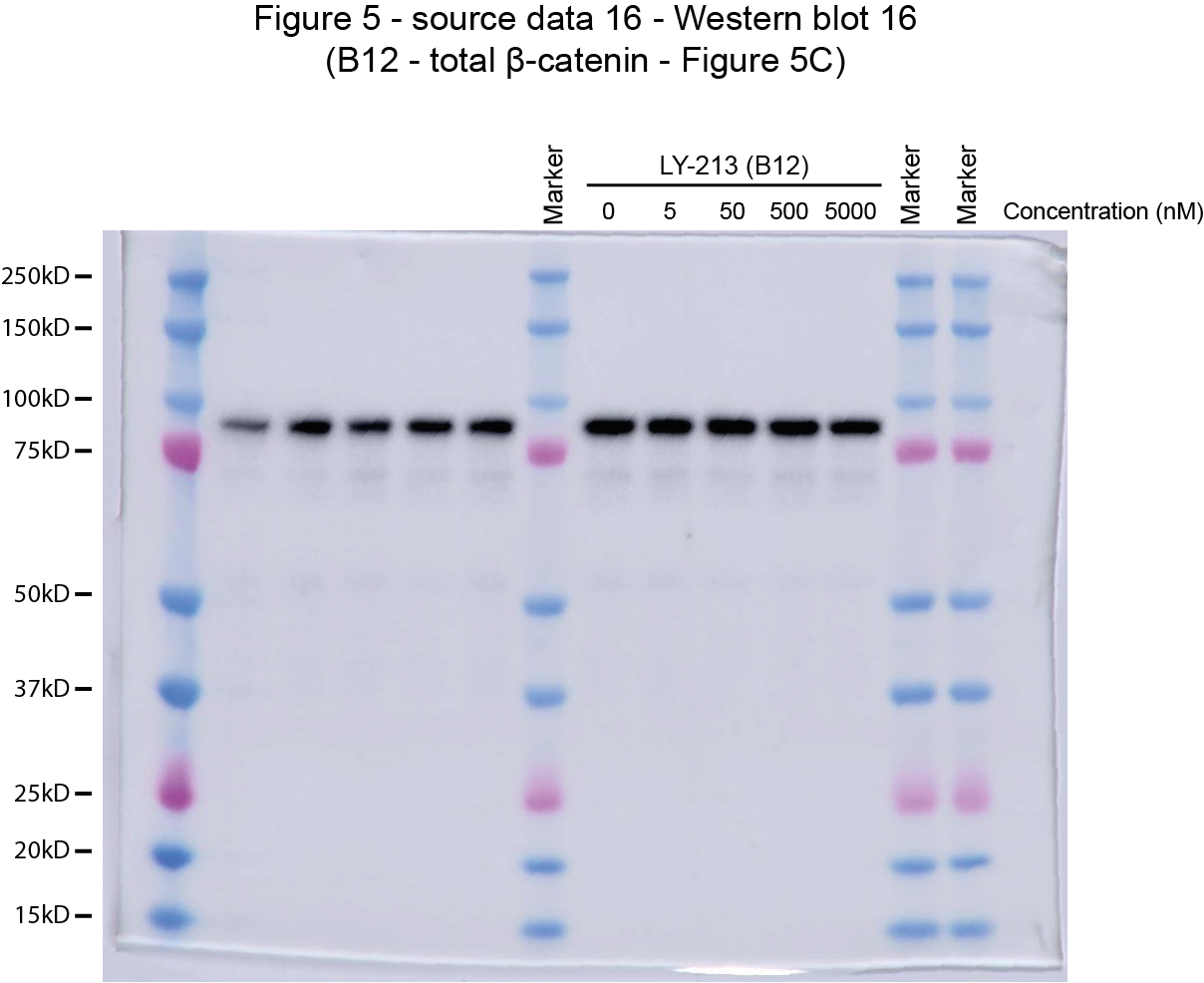

Supplement: Figure 5—source data 1. [file elife-88206-fig5-data1.zip › Figure 5 - source data/Figure 5 - source data 16/Western 16 - B12 - total bcatenin - labeled.png]

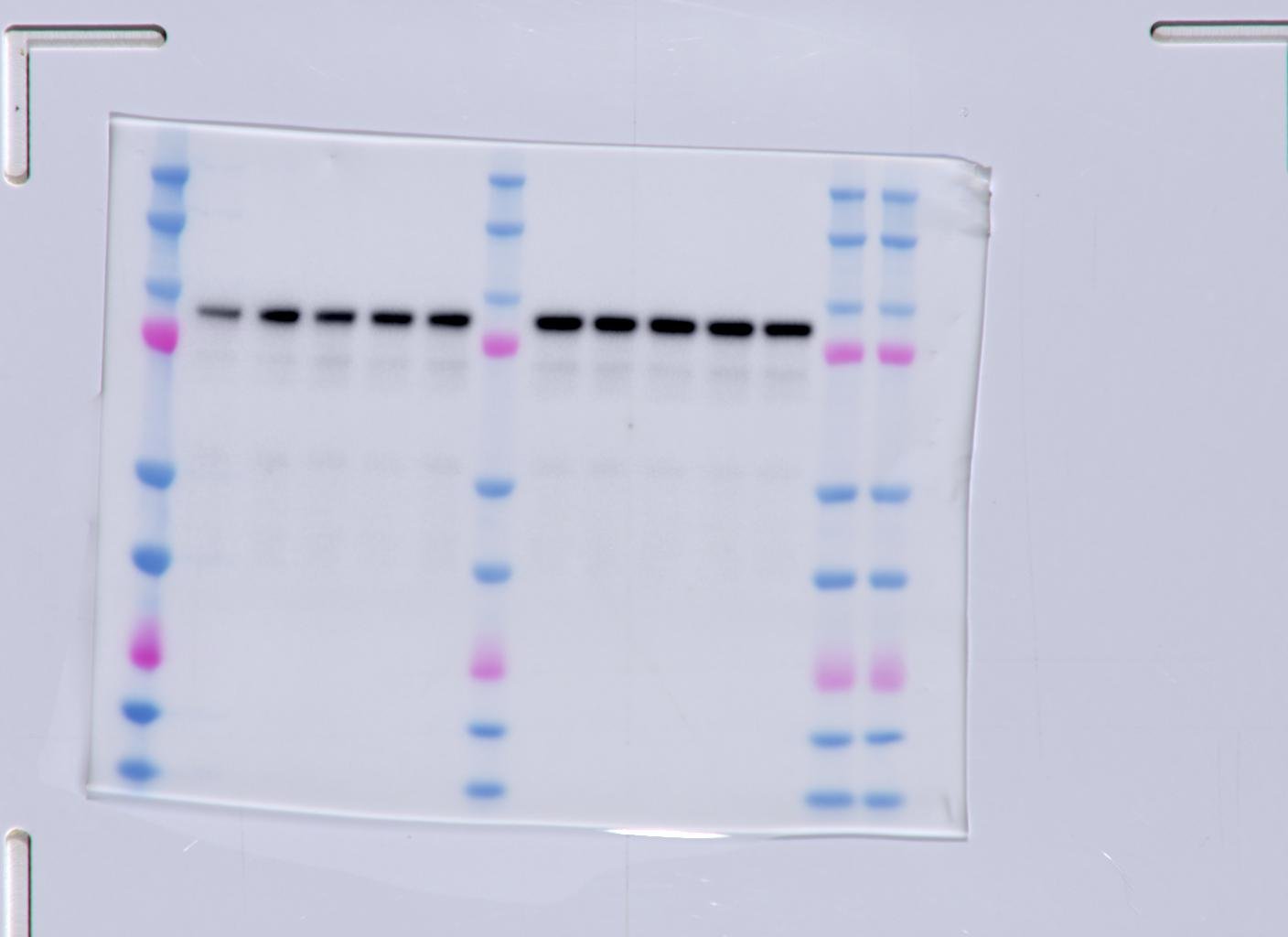

Supplement: Figure 5—source data 1. [file elife-88206-fig5-data1.zip › Figure 5 - source data/Figure 5 - source data 16/Western 16 - B12 - total bcatenin - uncropped.jpg]

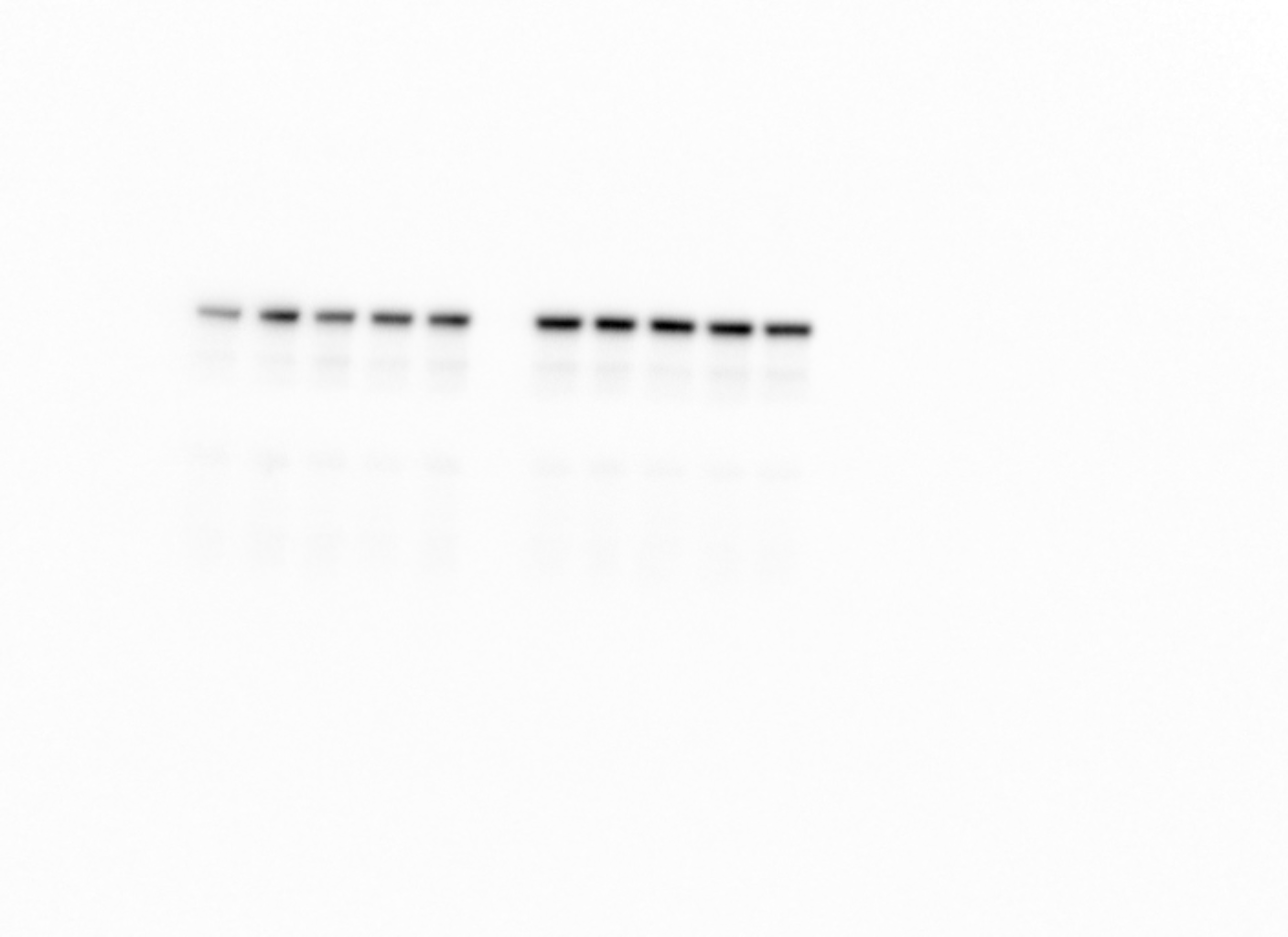

Supplement: Figure 5—source data 1. [file elife-88206-fig5-data1.zip › Figure 5 - source data/Figure 5 - source data 16/Western 16 - B12 - total bcatenin - uncropped.tif]

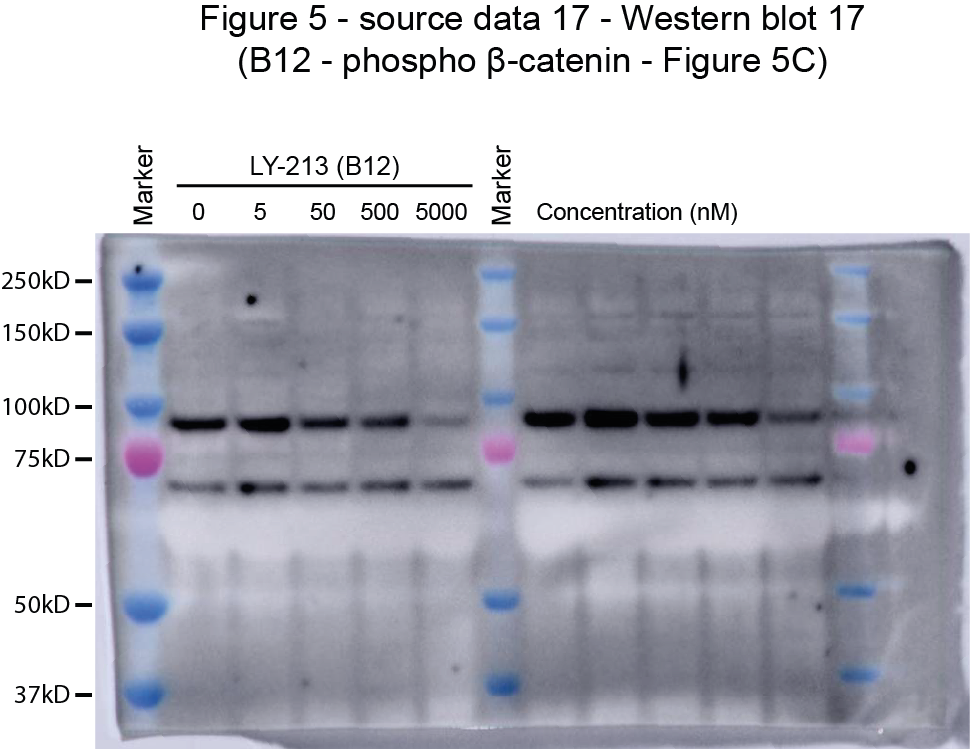

Supplement: Figure 5—source data 1. [file elife-88206-fig5-data1.zip › Figure 5 - source data/Figure 5 - source data 17/Western blot 17 - B12 - phospho bcatenin - labeled.png]

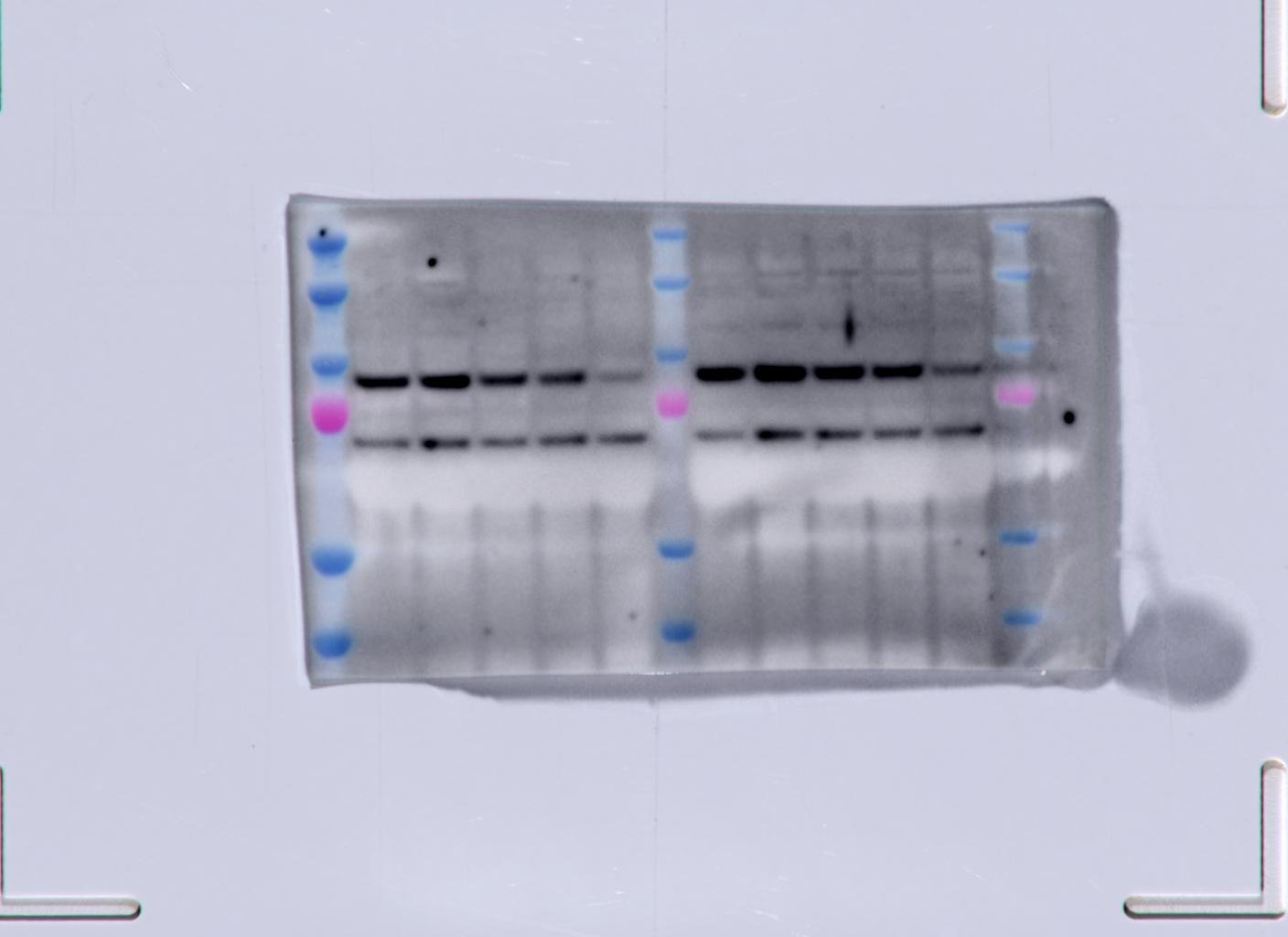

Supplement: Figure 5—source data 1. [file elife-88206-fig5-data1.zip › Figure 5 - source data/Figure 5 - source data 17/Western blot 17 - B12 - phospho bcatenin - uncropped.jpg]

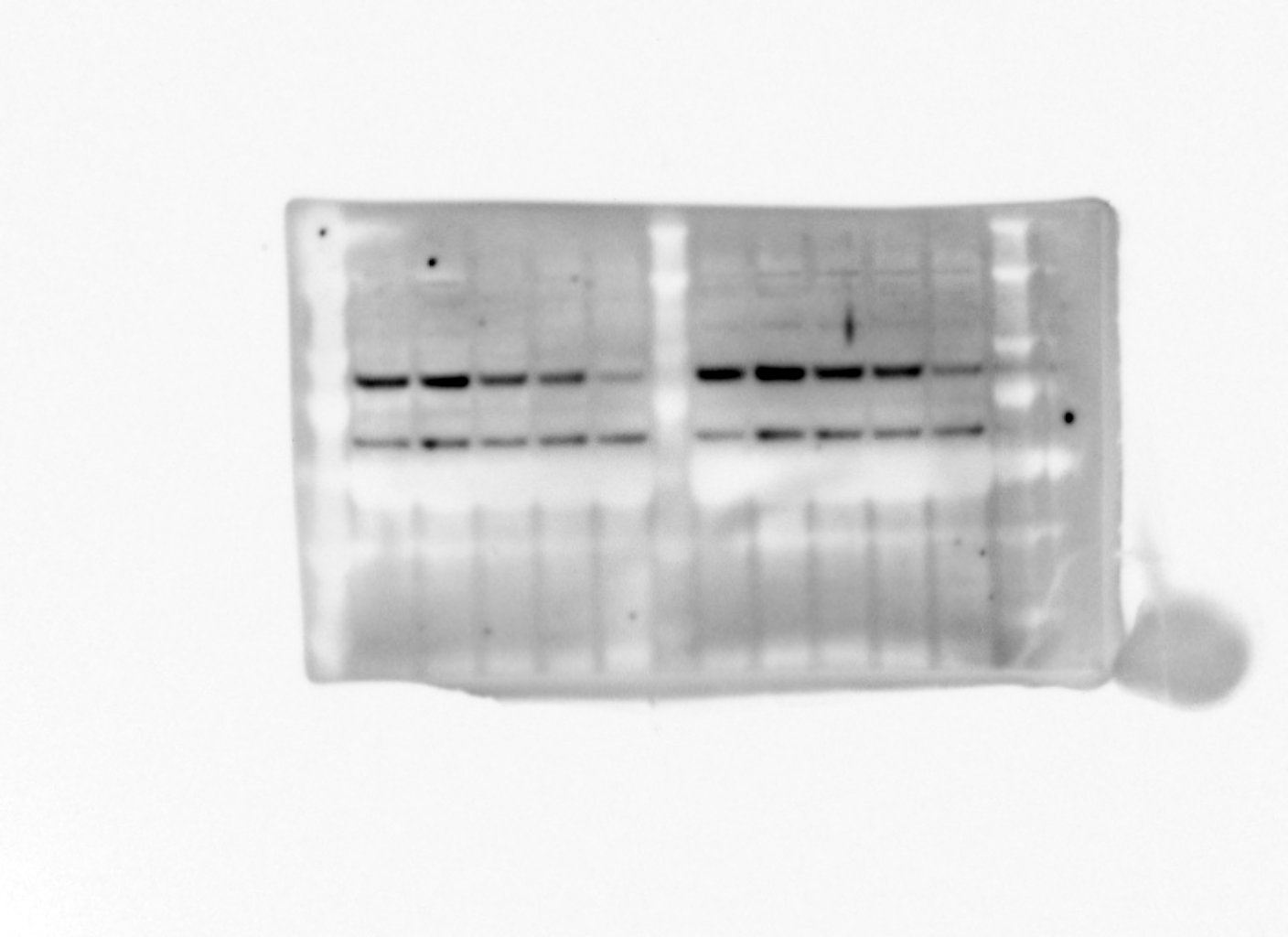

Supplement: Figure 5—source data 1. [file elife-88206-fig5-data1.zip › Figure 5 - source data/Figure 5 - source data 17/Western blot 17 - B12 - phospho bcatenin - uncropped.tif]

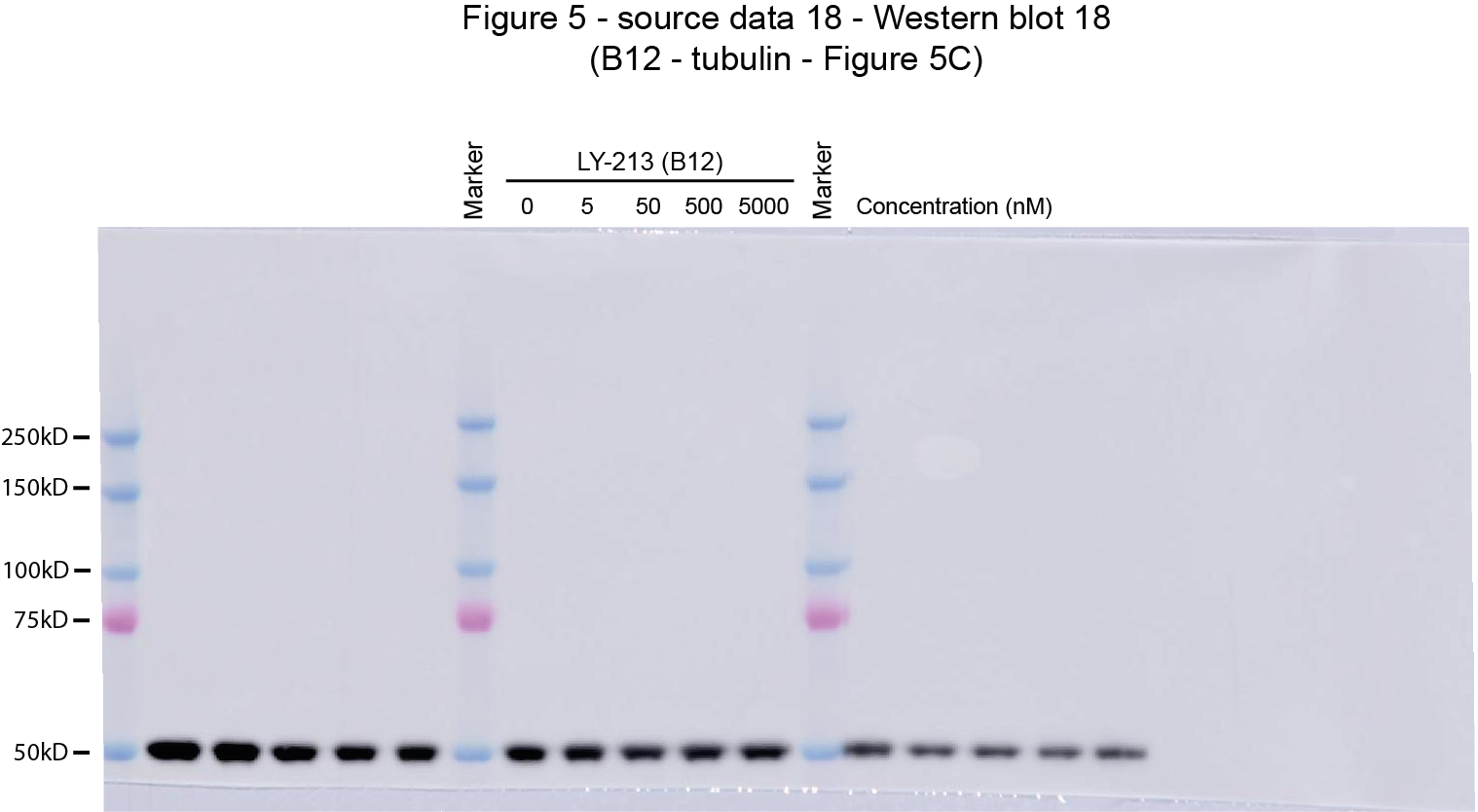

Supplement: Figure 5—source data 1. [file elife-88206-fig5-data1.zip › Figure 5 - source data/Figure 5 - source data 18/Western blot 18 - B12 - tubulin- labeled.png]

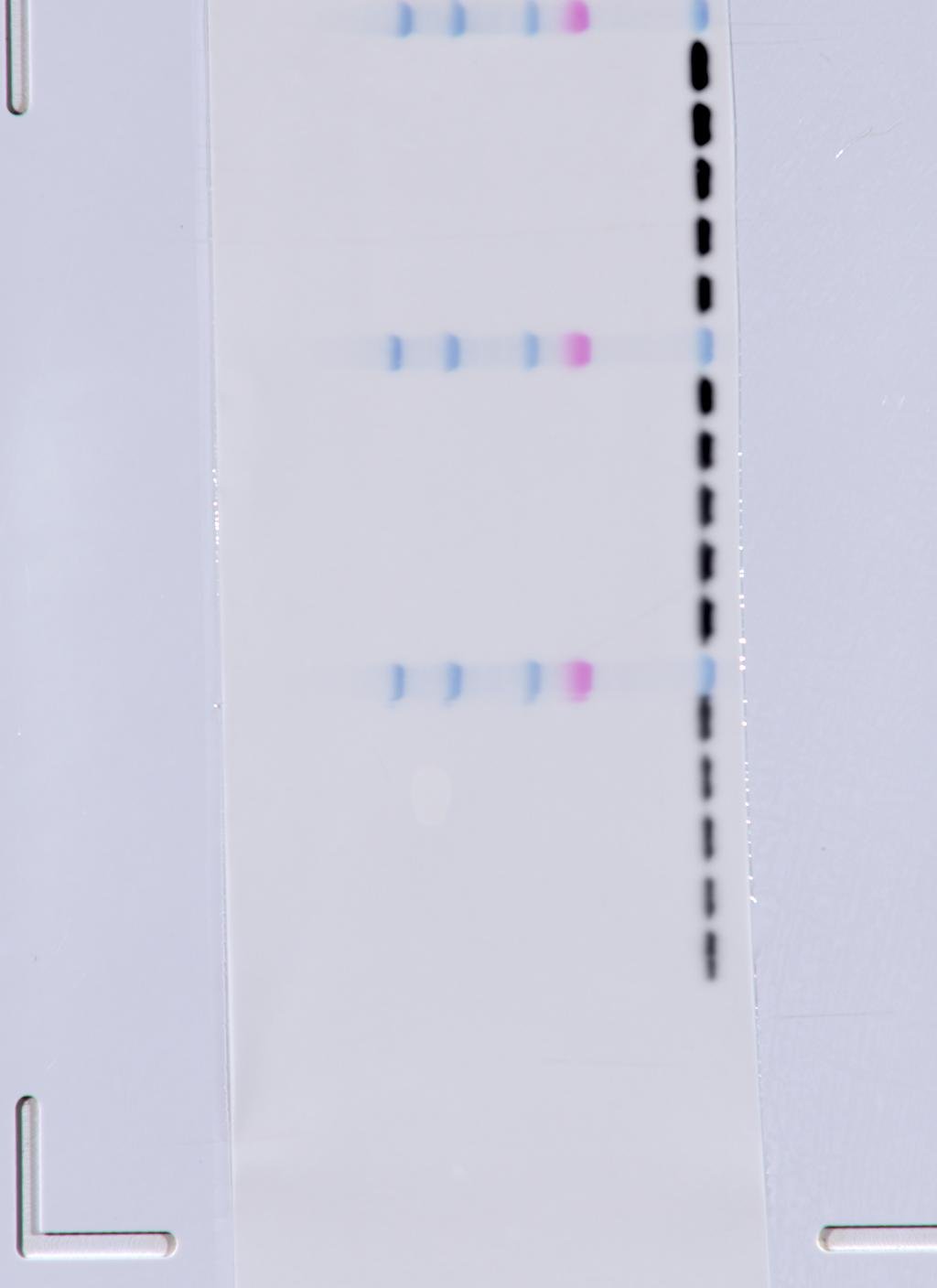

Supplement: Figure 5—source data 1. [file elife-88206-fig5-data1.zip › Figure 5 - source data/Figure 5 - source data 18/Western blot 18 - B12 - tubulin- uncropped.jpg]

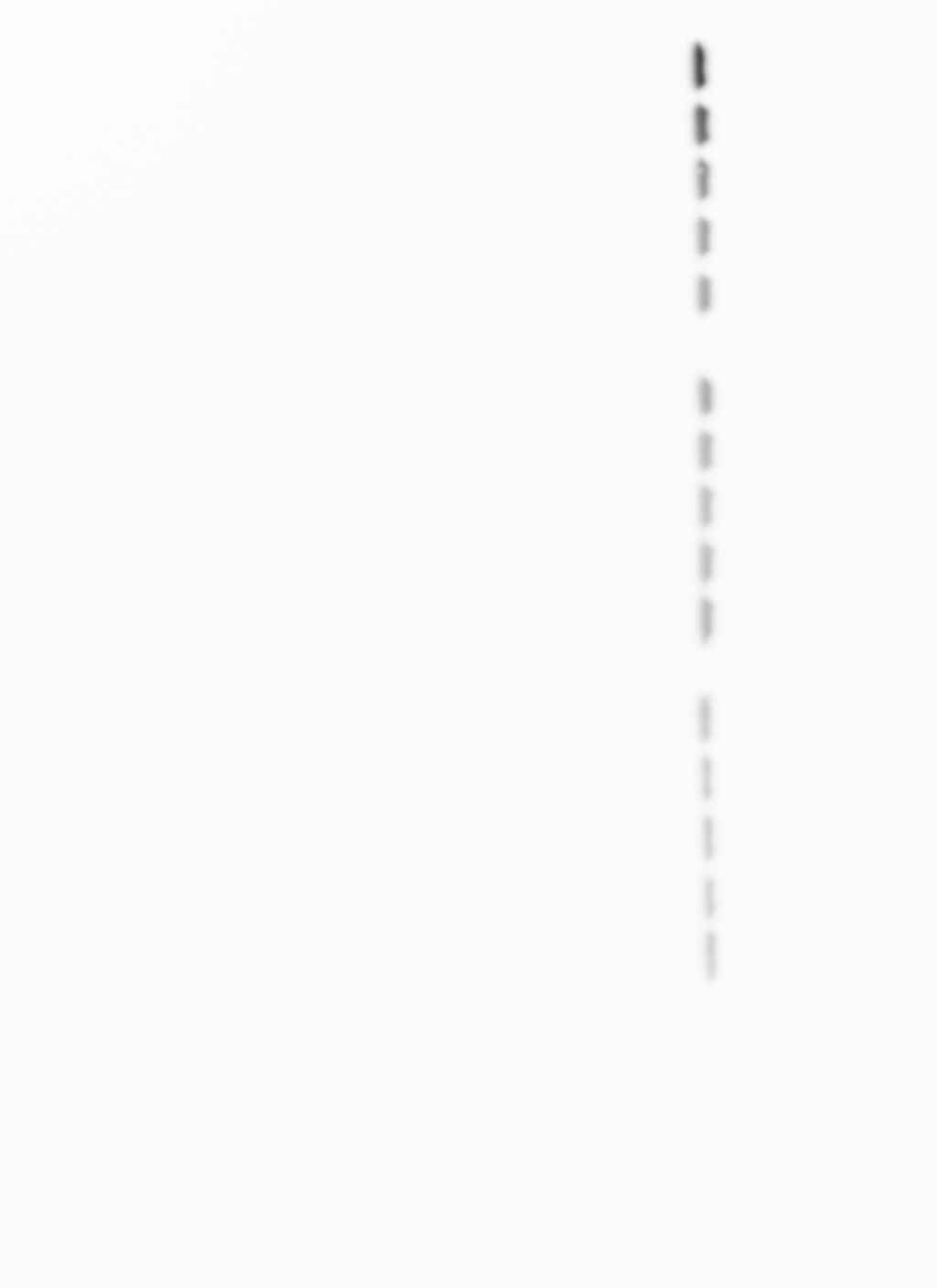

Supplement: Figure 5—source data 1. [file elife-88206-fig5-data1.zip › Figure 5 - source data/Figure 5 - source data 18/Western blot 18 - B12 - tubulin- uncropped.tif]

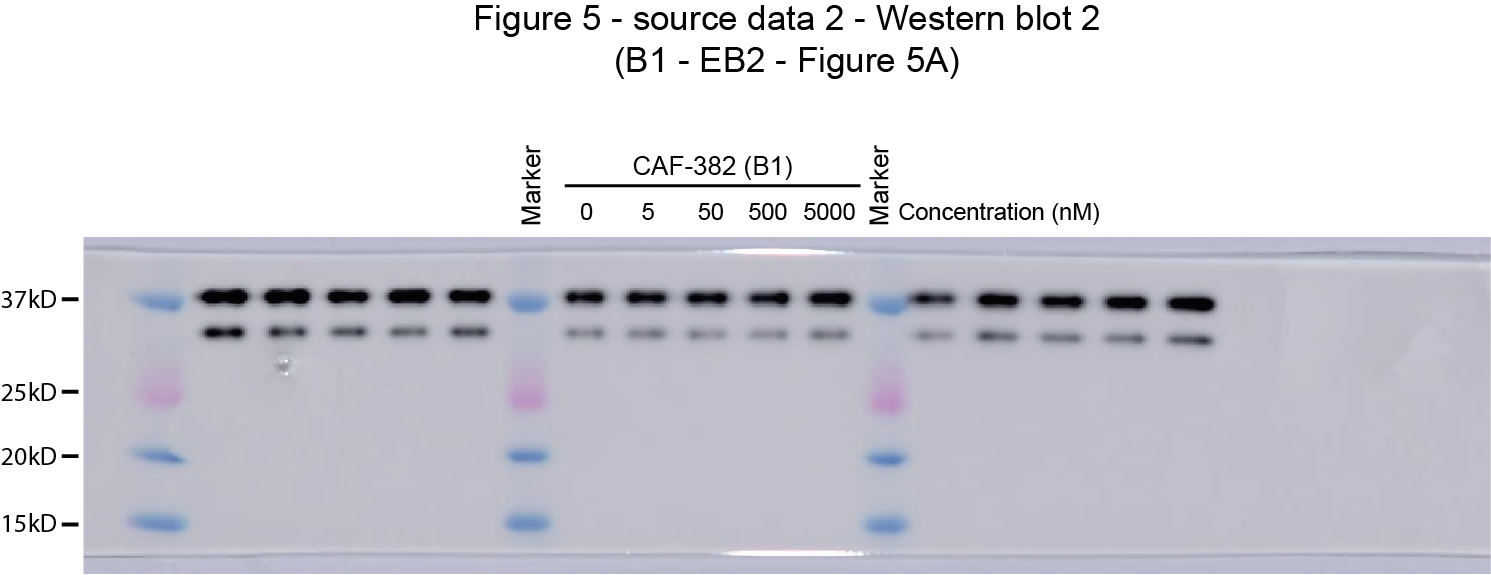

Supplement: Figure 5—source data 1. [file elife-88206-fig5-data1.zip › Figure 5 - source data/Figure 5 - source data 2/Western blot 2 - B1 - EB2 - labeled.png]

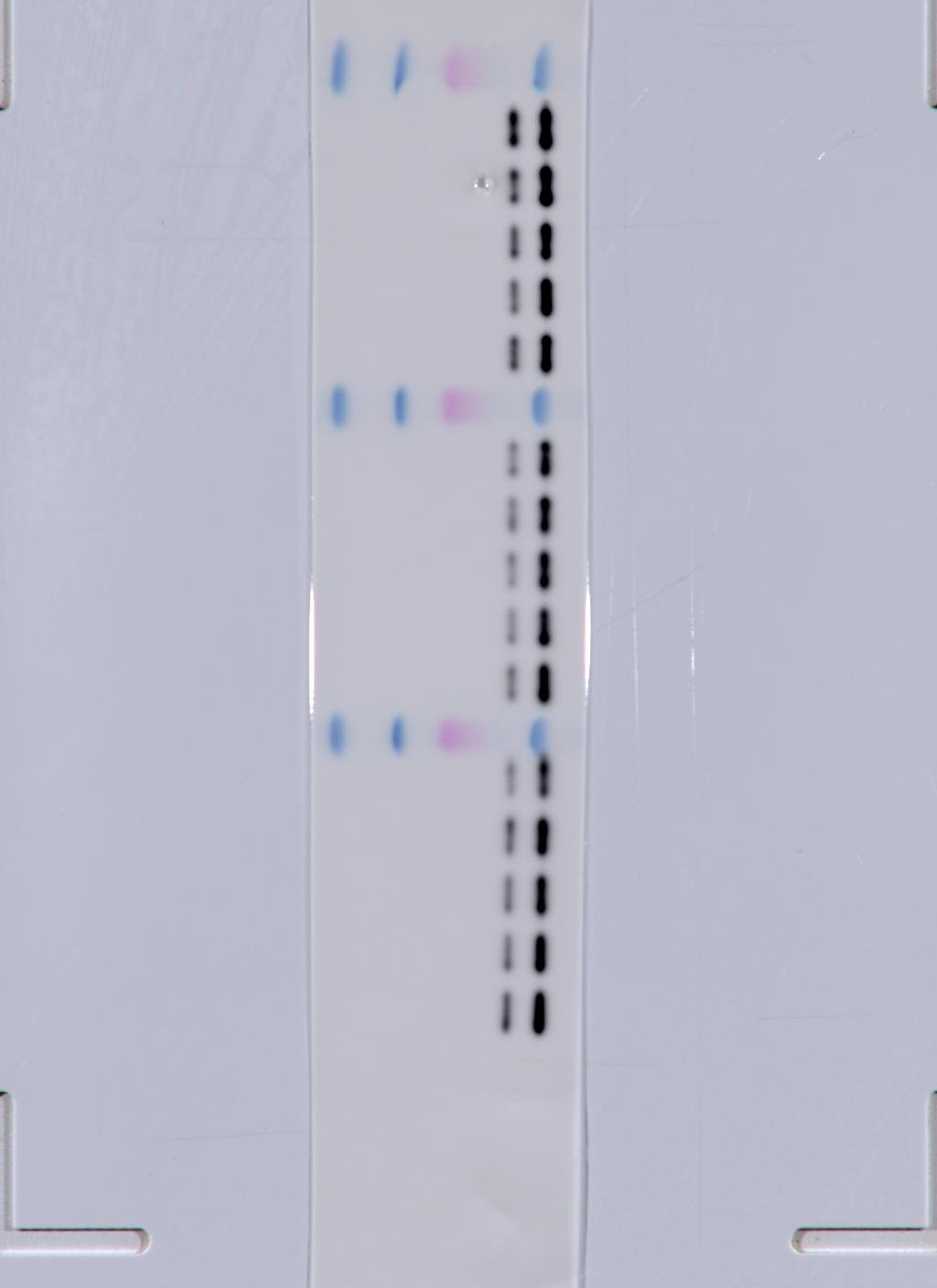

Supplement: Figure 5—source data 1. [file elife-88206-fig5-data1.zip › Figure 5 - source data/Figure 5 - source data 2/Western blot 2 - B1 - EB2 - uncropped.jpg]

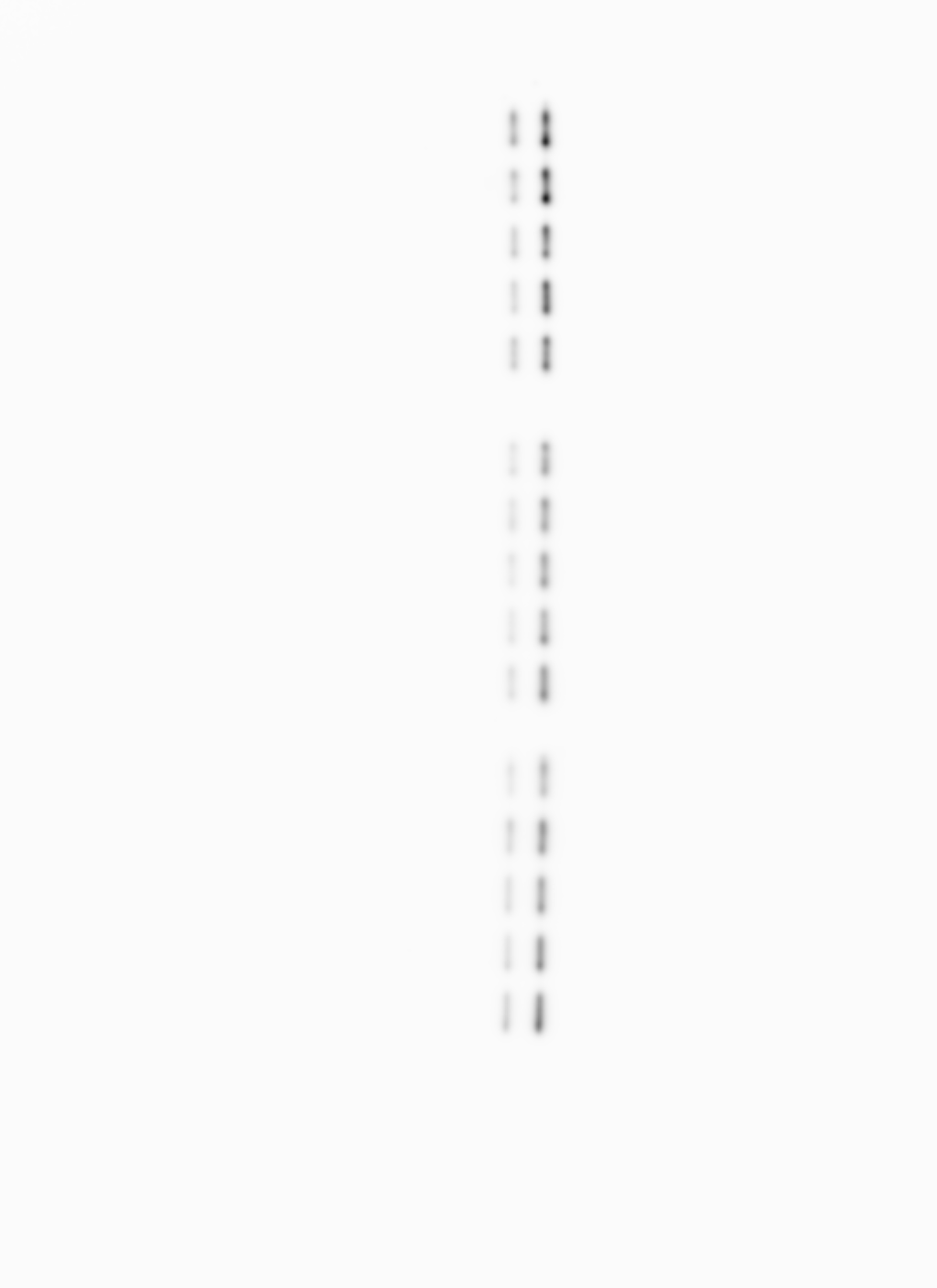

Supplement: Figure 5—source data 1. [file elife-88206-fig5-data1.zip › Figure 5 - source data/Figure 5 - source data 2/Western blot 2 - B1 - EB2 - uncropped.tif]

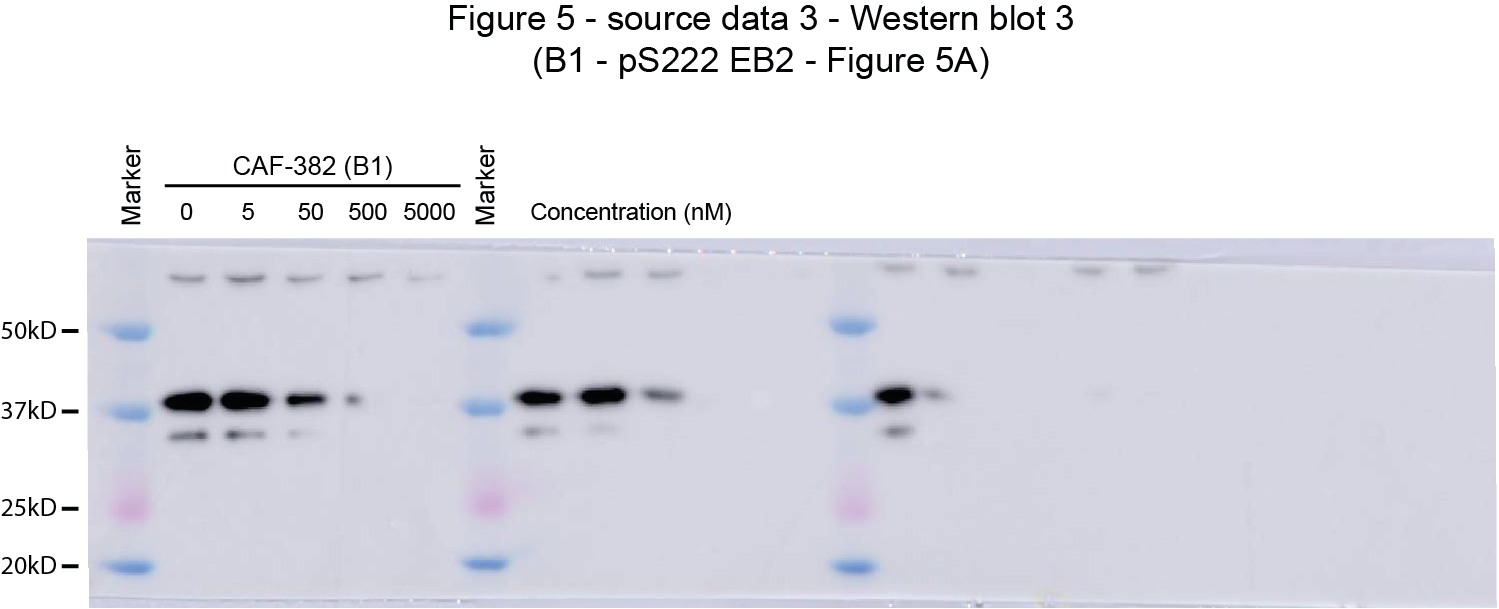

Supplement: Figure 5—source data 1. [file elife-88206-fig5-data1.zip › Figure 5 - source data/Figure 5 - source data 3/Western blot 3 - B1 - pEB2 - labeled.png]

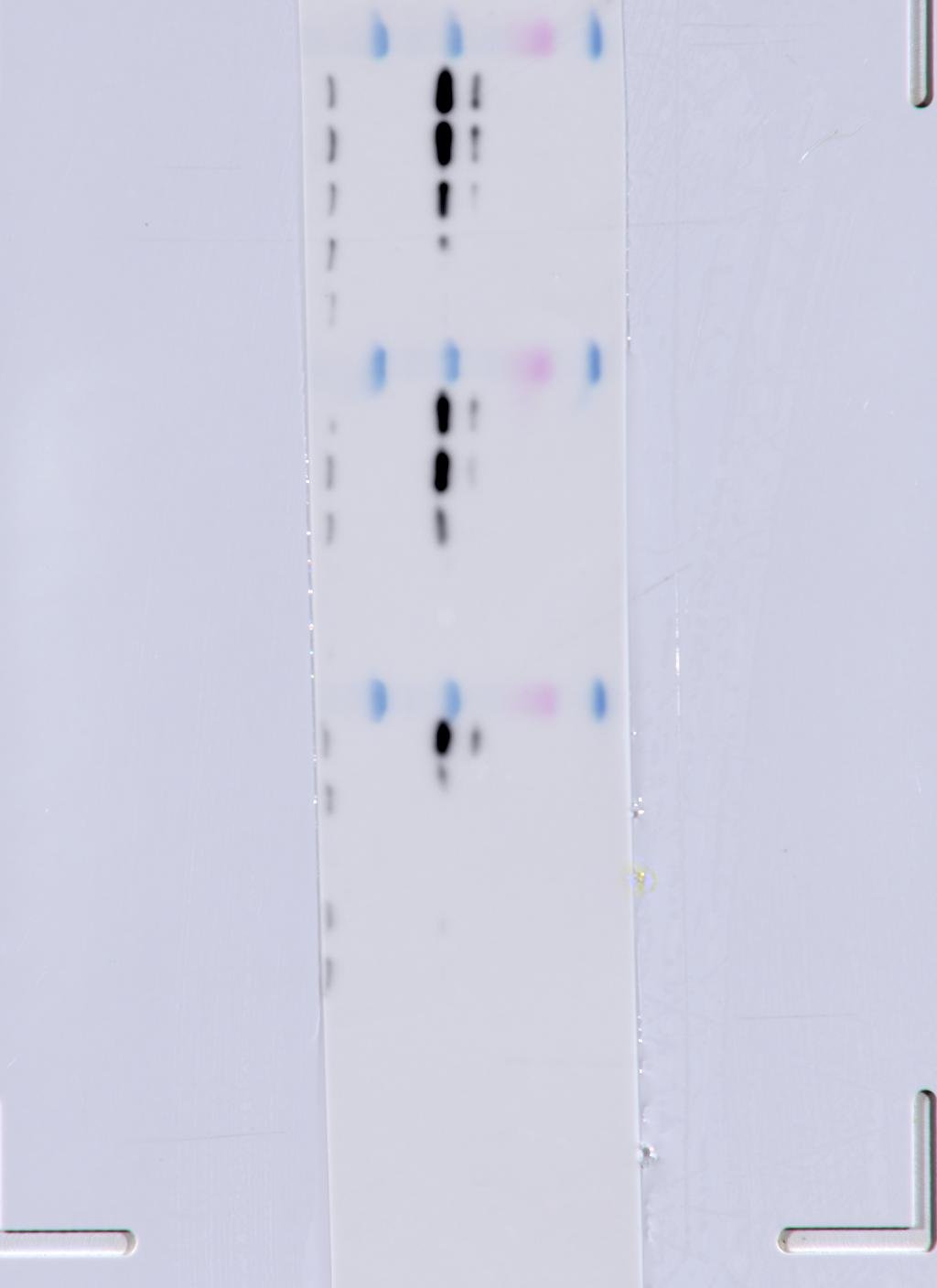

Supplement: Figure 5—source data 1. [file elife-88206-fig5-data1.zip › Figure 5 - source data/Figure 5 - source data 3/Western blot 3 - B1 - pEB2 - uncropped.jpg]

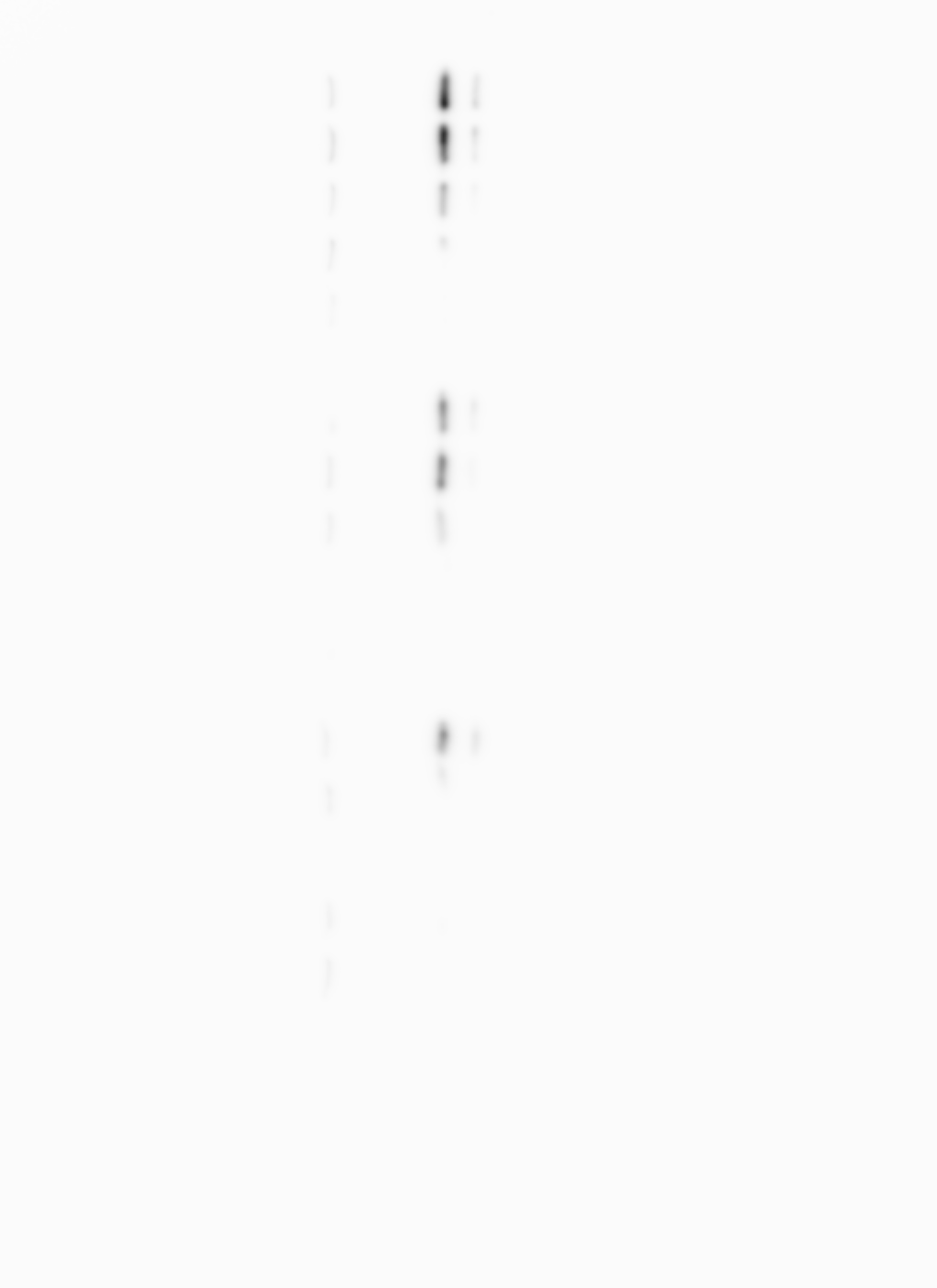

Supplement: Figure 5—source data 1. [file elife-88206-fig5-data1.zip › Figure 5 - source data/Figure 5 - source data 3/Western blot 3 - B1 - pEB2 - uncropped.tif]

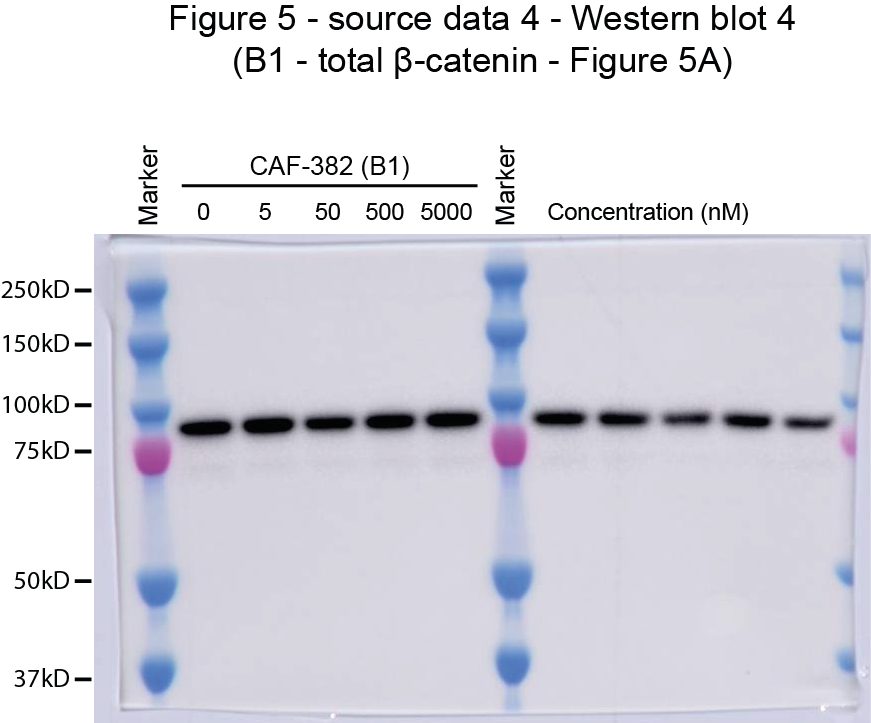

Supplement: Figure 5—source data 1. [file elife-88206-fig5-data1.zip › Figure 5 - source data/Figure 5 - source data 4/Western blot 4 - B1 - total bcatenin - labeled.png]

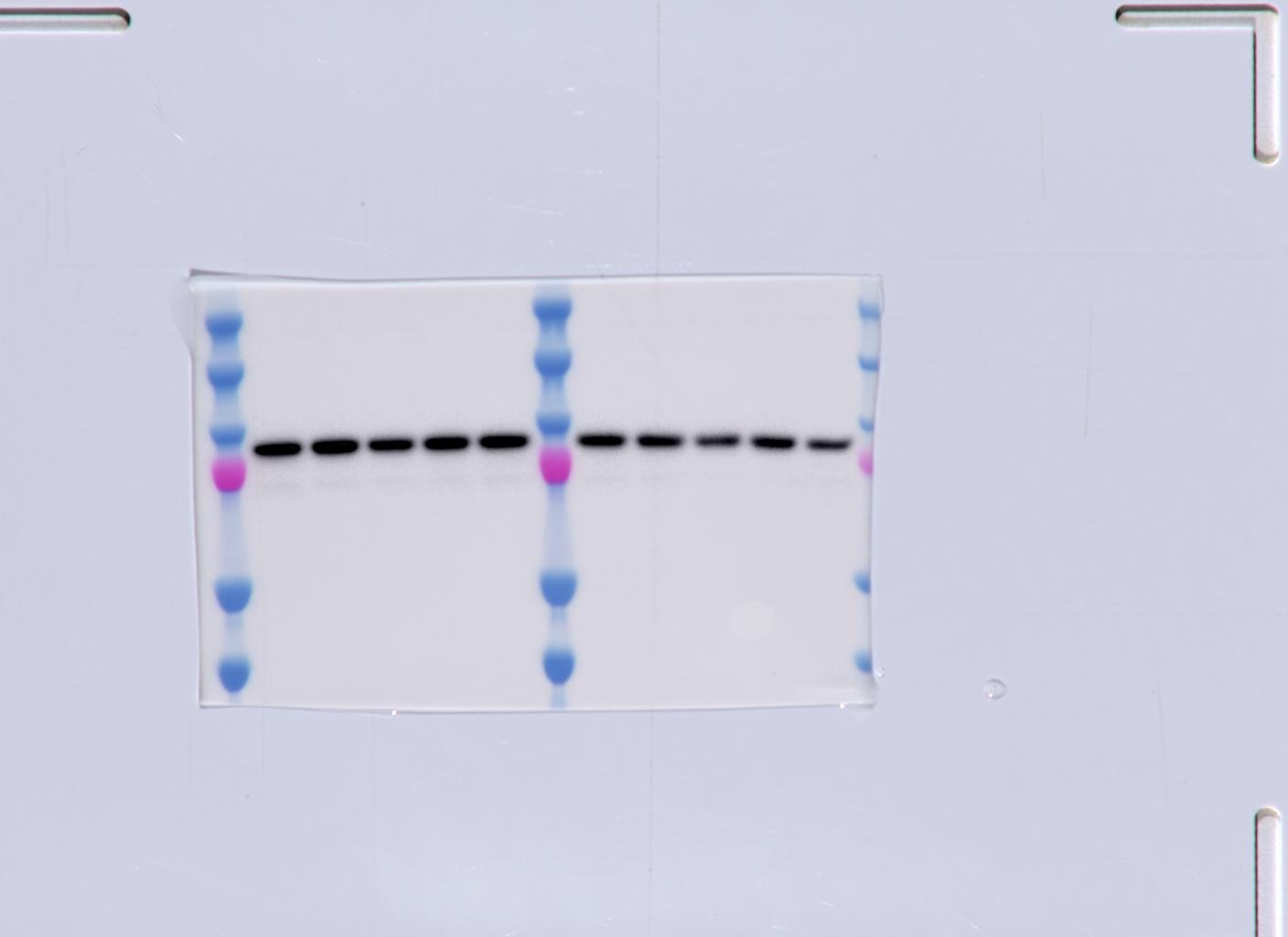

Supplement: Figure 5—source data 1. [file elife-88206-fig5-data1.zip › Figure 5 - source data/Figure 5 - source data 4/Western blot 4 - B1 - total bcatenin - uncropped.jpg]

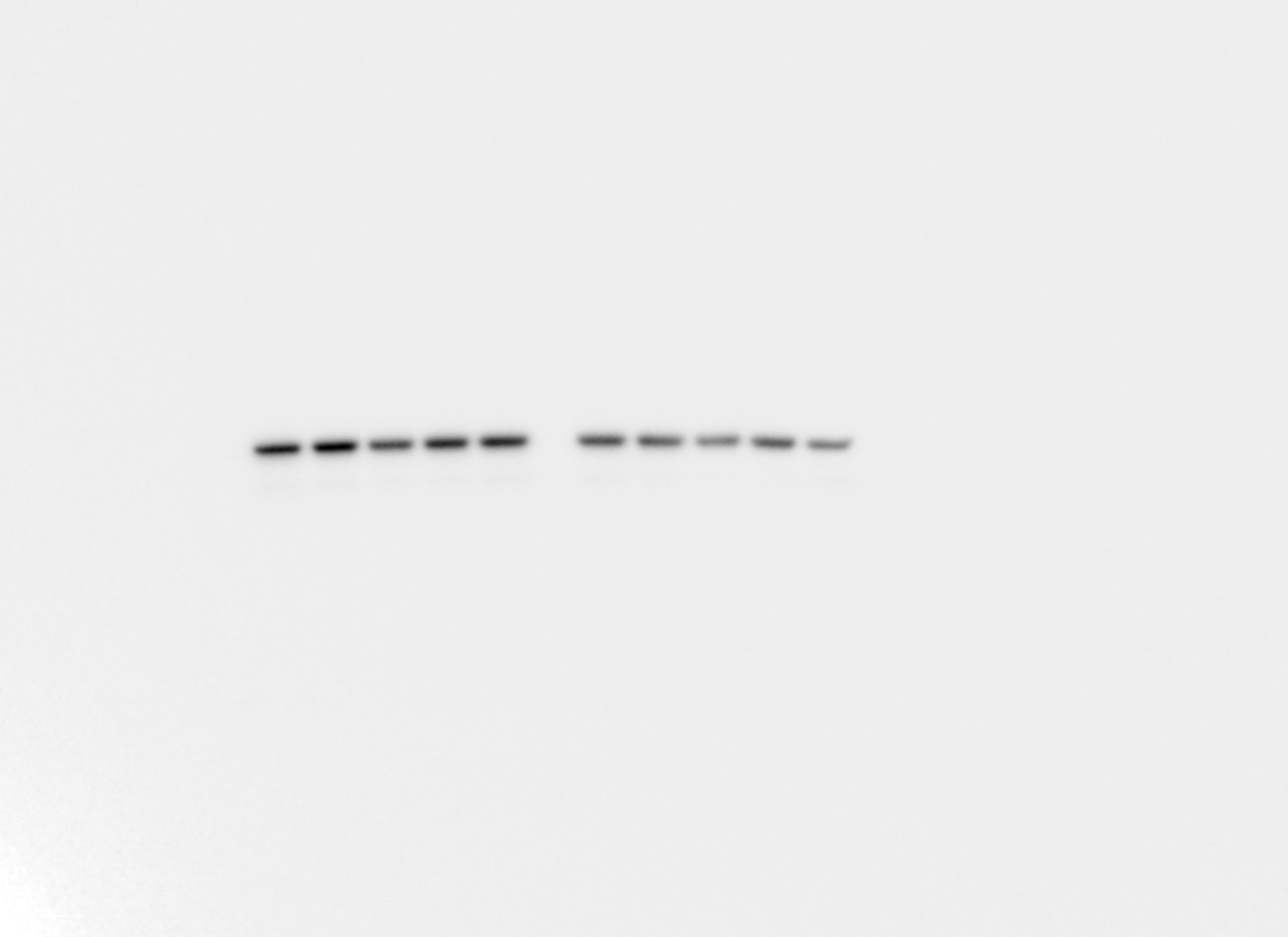

Supplement: Figure 5—source data 1. [file elife-88206-fig5-data1.zip › Figure 5 - source data/Figure 5 - source data 4/Western blot 4 - B1 - total bcatenin - uncropped.tif]

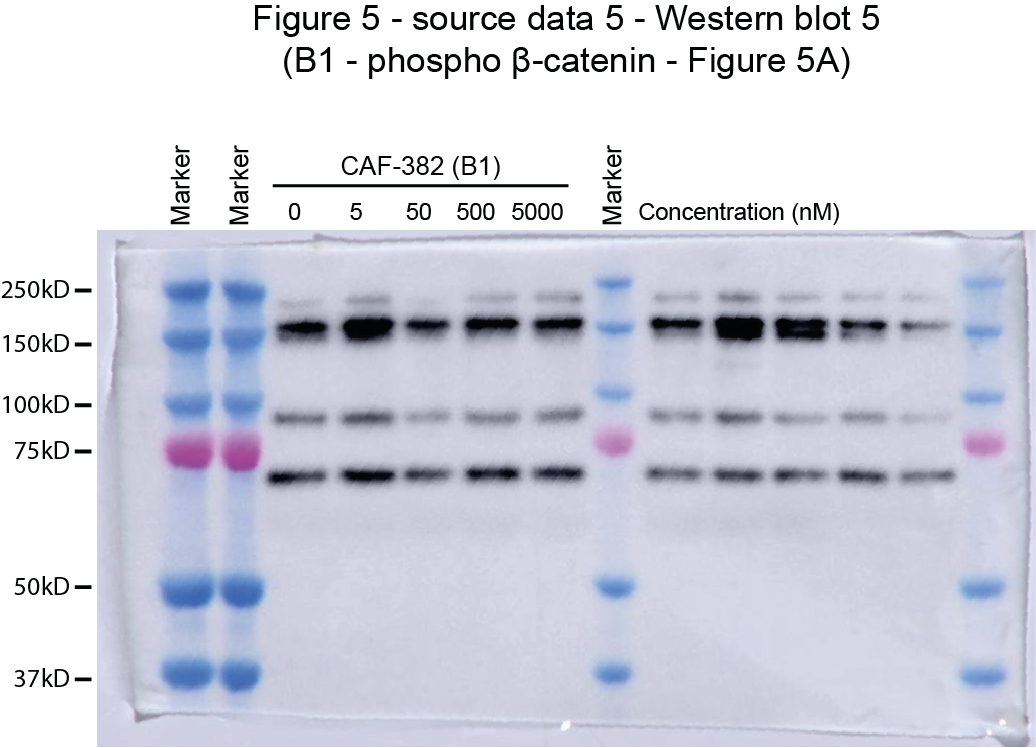

Supplement: Figure 5—source data 1. [file elife-88206-fig5-data1.zip › Figure 5 - source data/Figure 5 - source data 5/Western blot 5 - B1 - phospho bcatenin - labeled.png]

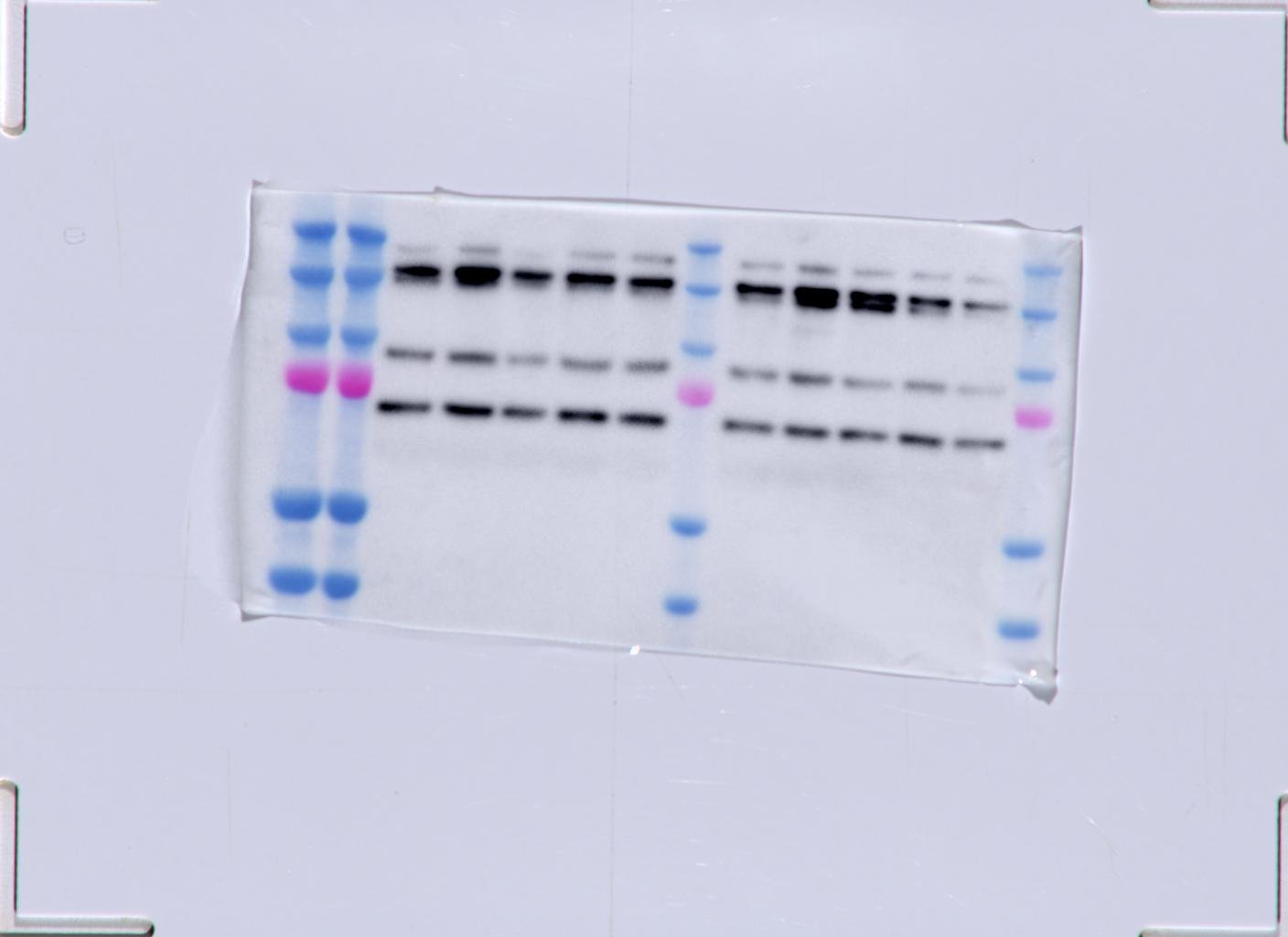

Supplement: Figure 5—source data 1. [file elife-88206-fig5-data1.zip › Figure 5 - source data/Figure 5 - source data 5/Western blot 5 - B1 - phospho bcatenin - uncropped.jpg]

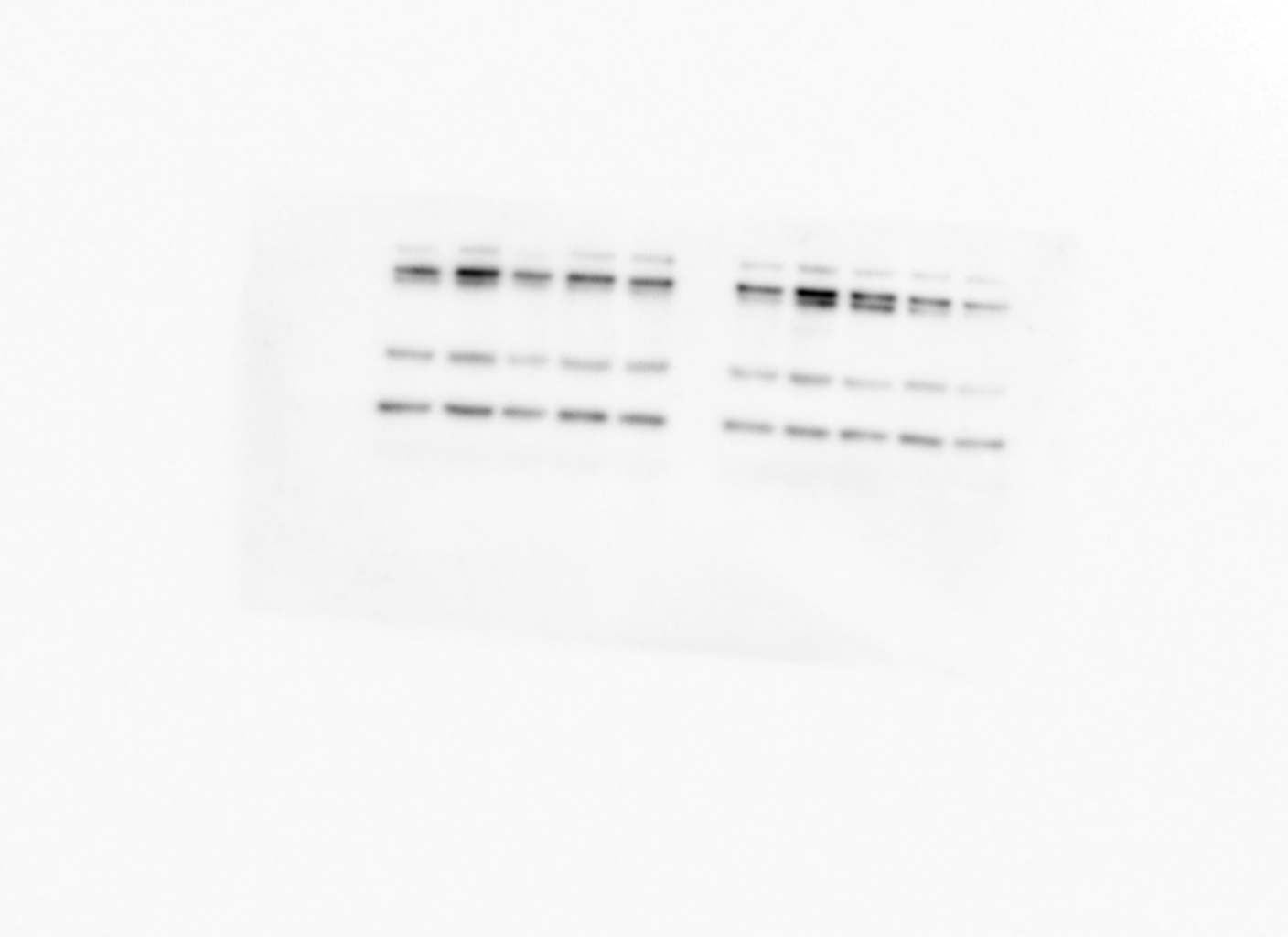

Supplement: Figure 5—source data 1. [file elife-88206-fig5-data1.zip › Figure 5 - source data/Figure 5 - source data 5/Western blot 5 - B1 - phospho bcatenin - uncropped.tif]

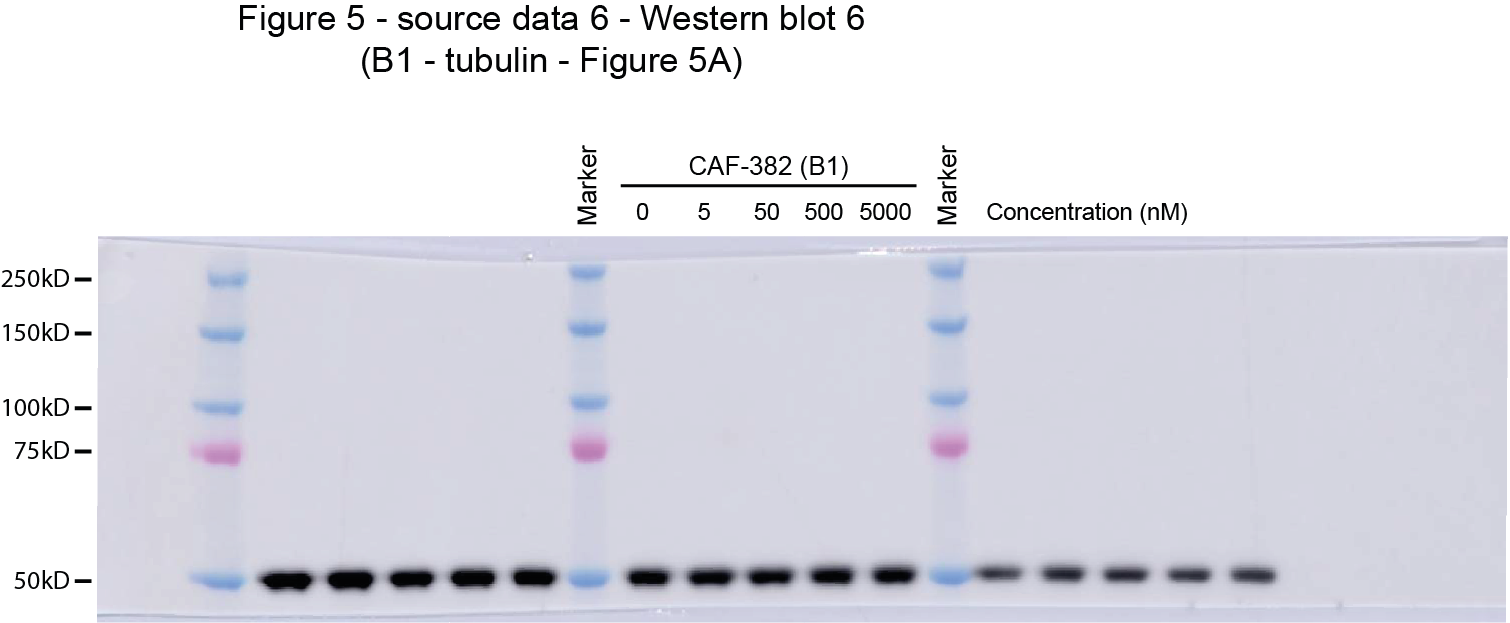

Supplement: Figure 5—source data 1. [file elife-88206-fig5-data1.zip › Figure 5 - source data/Figure 5 - source data 6/Western blot 6 - B1 - tubulin - labeled .png]

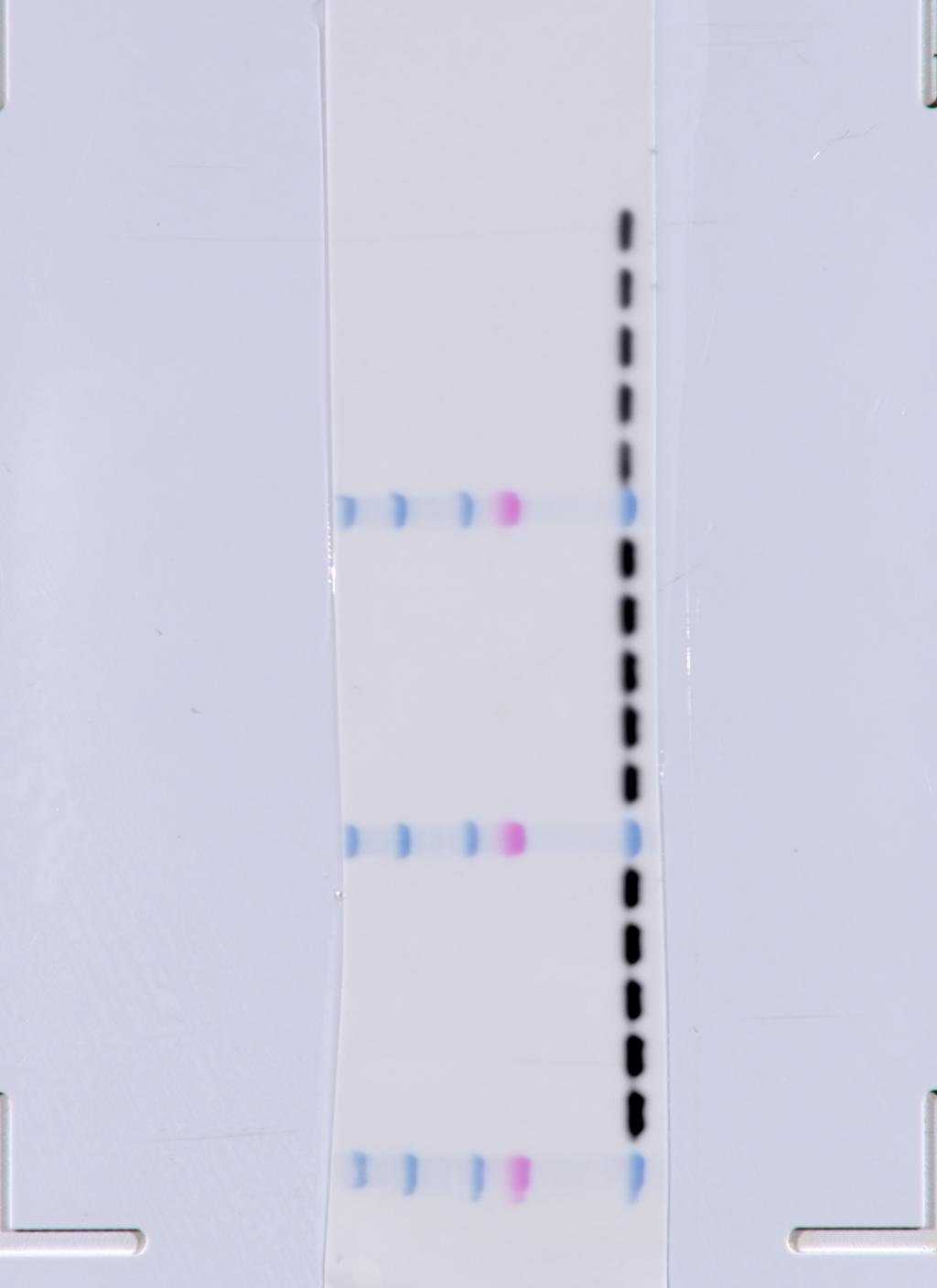

Supplement: Figure 5—source data 1. [file elife-88206-fig5-data1.zip › Figure 5 - source data/Figure 5 - source data 6/Western blot 6 - B1 - tubulin - uncropped.jpg]

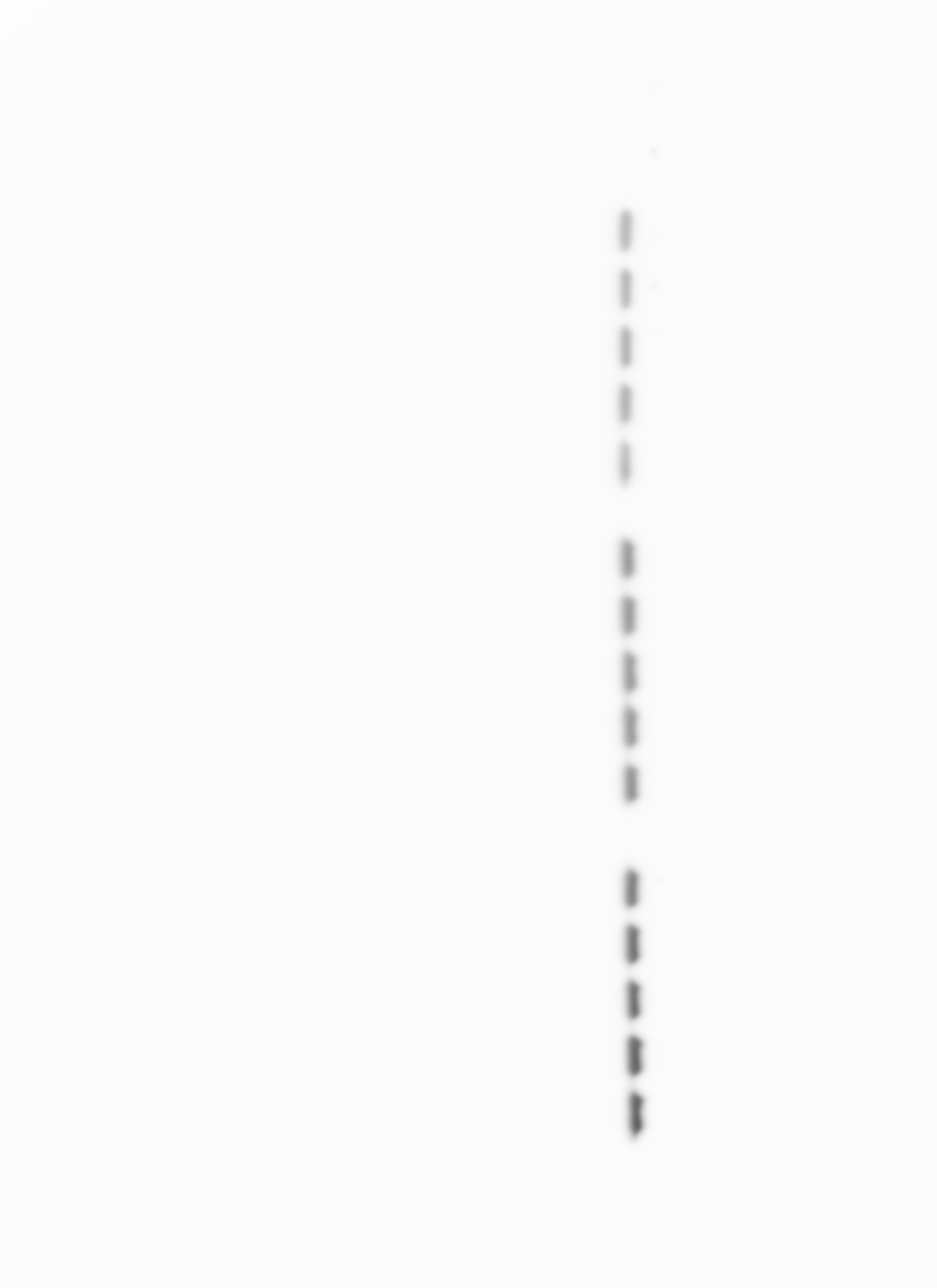

Supplement: Figure 5—source data 1. [file elife-88206-fig5-data1.zip › Figure 5 - source data/Figure 5 - source data 6/Western blot 6 - B1 - tubulin - uncropped.tif]

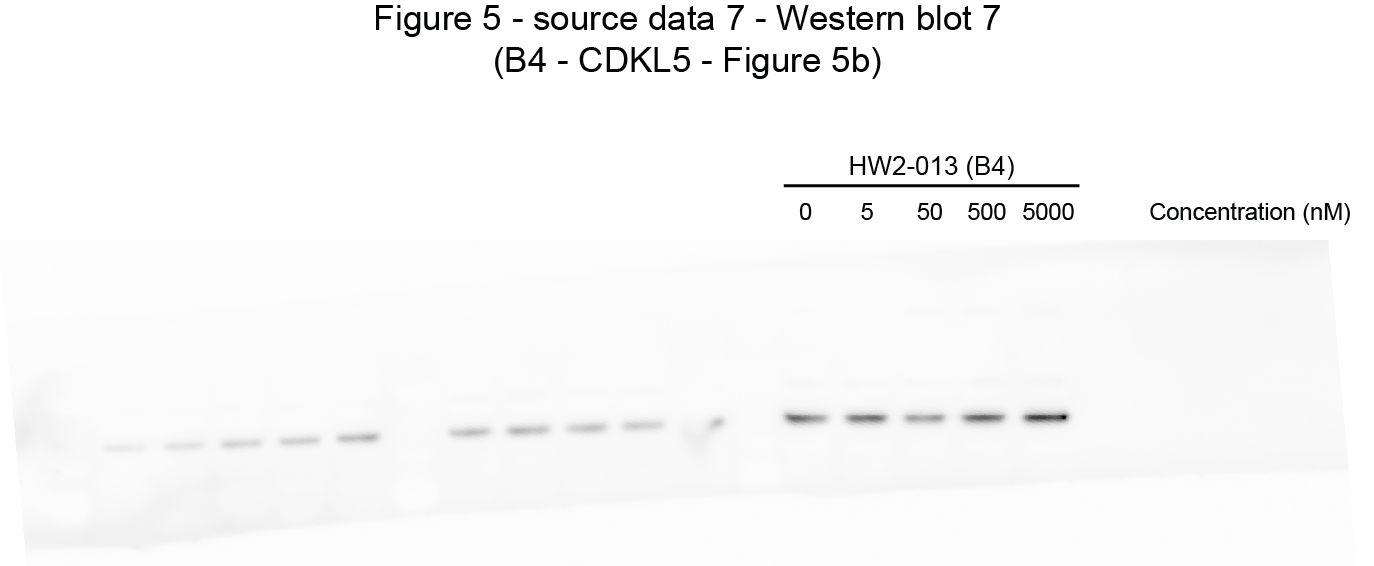

Supplement: Figure 5—source data 1. [file elife-88206-fig5-data1.zip › Figure 5 - source data/Figure 5 - source data 7/Western blot 7 - B4 - CDKL5 - labeled.png]

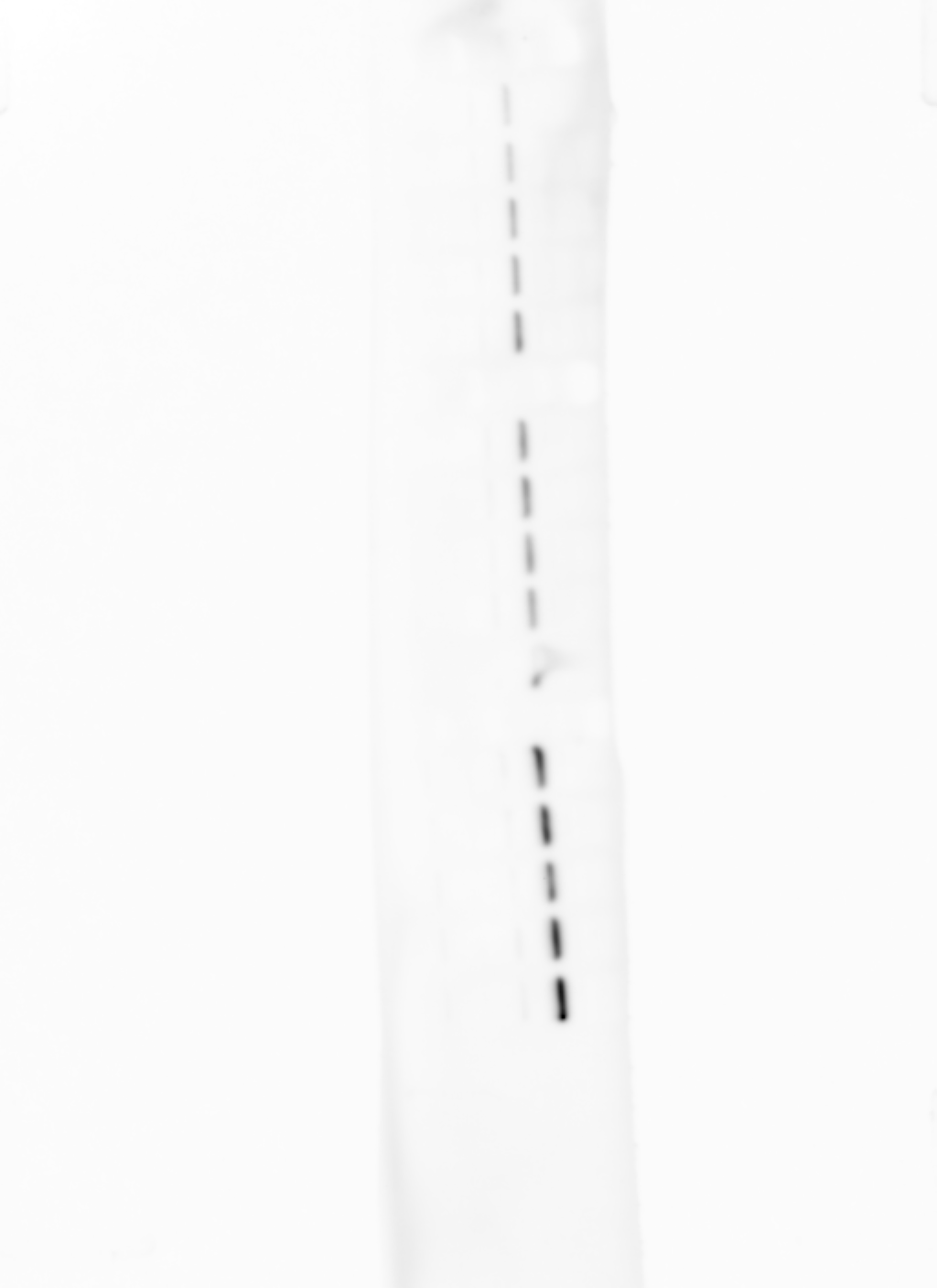

Supplement: Figure 5—source data 1. [file elife-88206-fig5-data1.zip › Figure 5 - source data/Figure 5 - source data 7/Western blot 7 - B4 - CDKL5 - uncropped.tif]

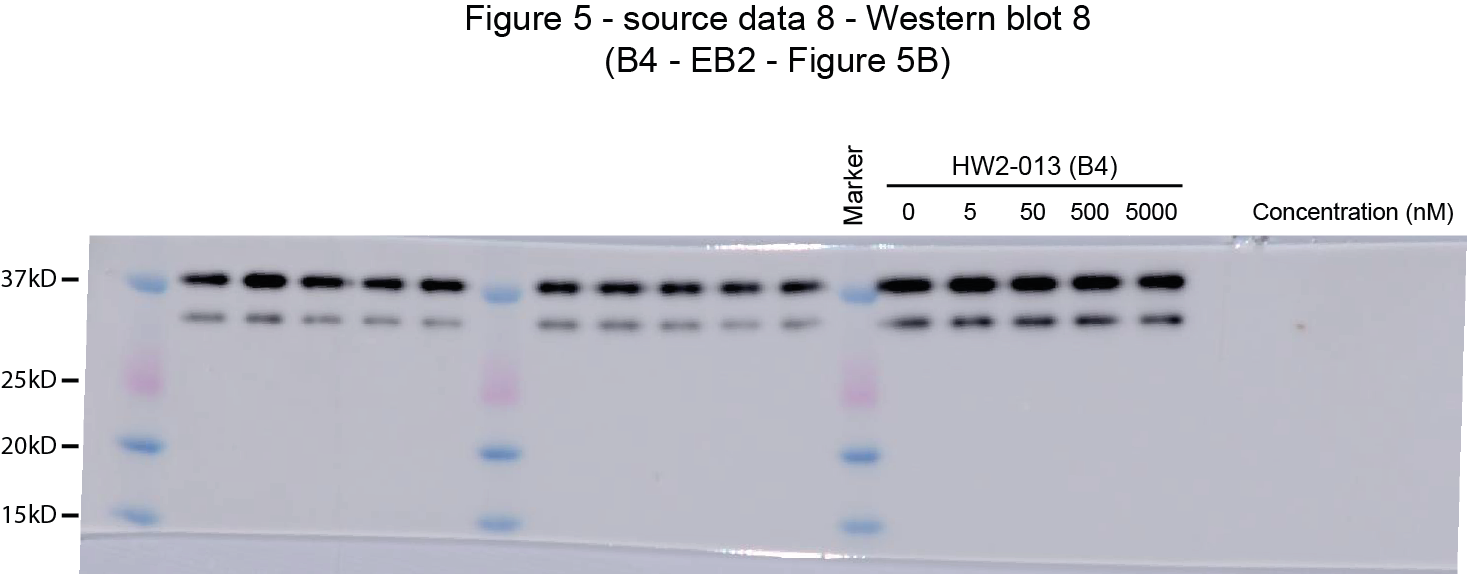

Supplement: Figure 5—source data 1. [file elife-88206-fig5-data1.zip › Figure 5 - source data/Figure 5 - source data 8/Western blot 8 - B4 - EB2 - labeled.png]

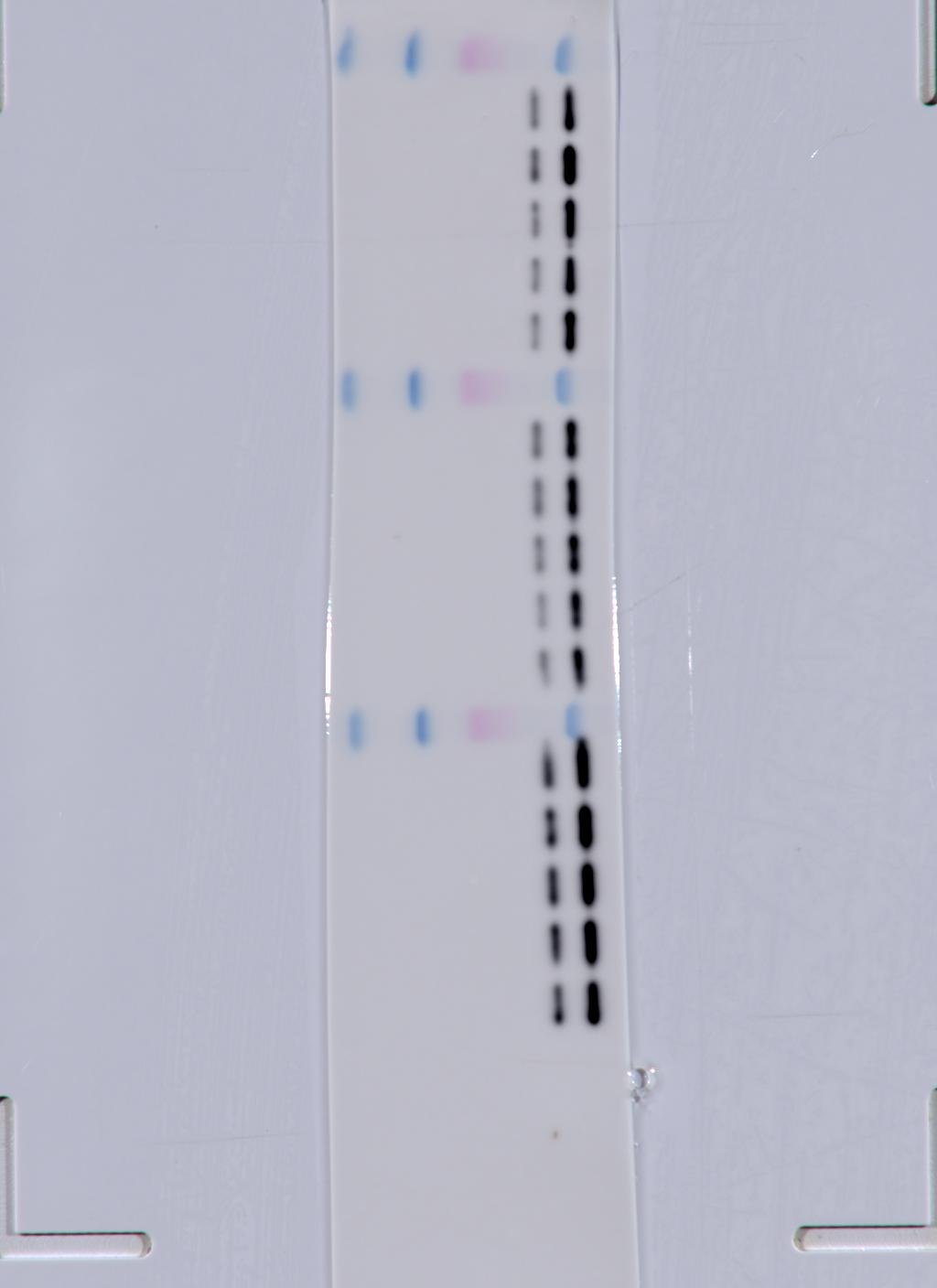

Supplement: Figure 5—source data 1. [file elife-88206-fig5-data1.zip › Figure 5 - source data/Figure 5 - source data 8/Western blot 8 - B4 - EB2 - uncropped.jpg]

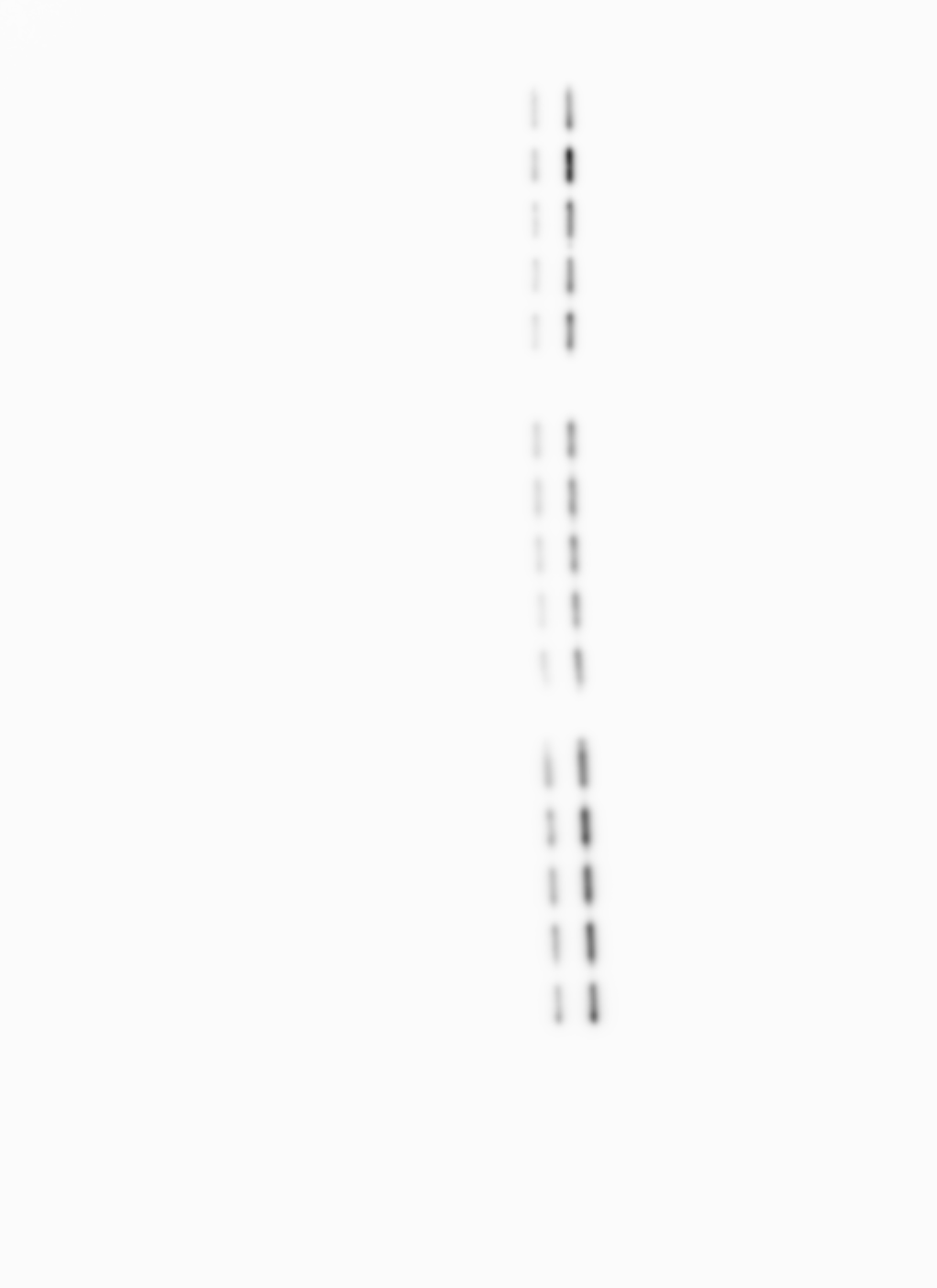

Supplement: Figure 5—source data 1. [file elife-88206-fig5-data1.zip › Figure 5 - source data/Figure 5 - source data 8/Western blot 8 - B4 - EB2 - uncropped.tif]

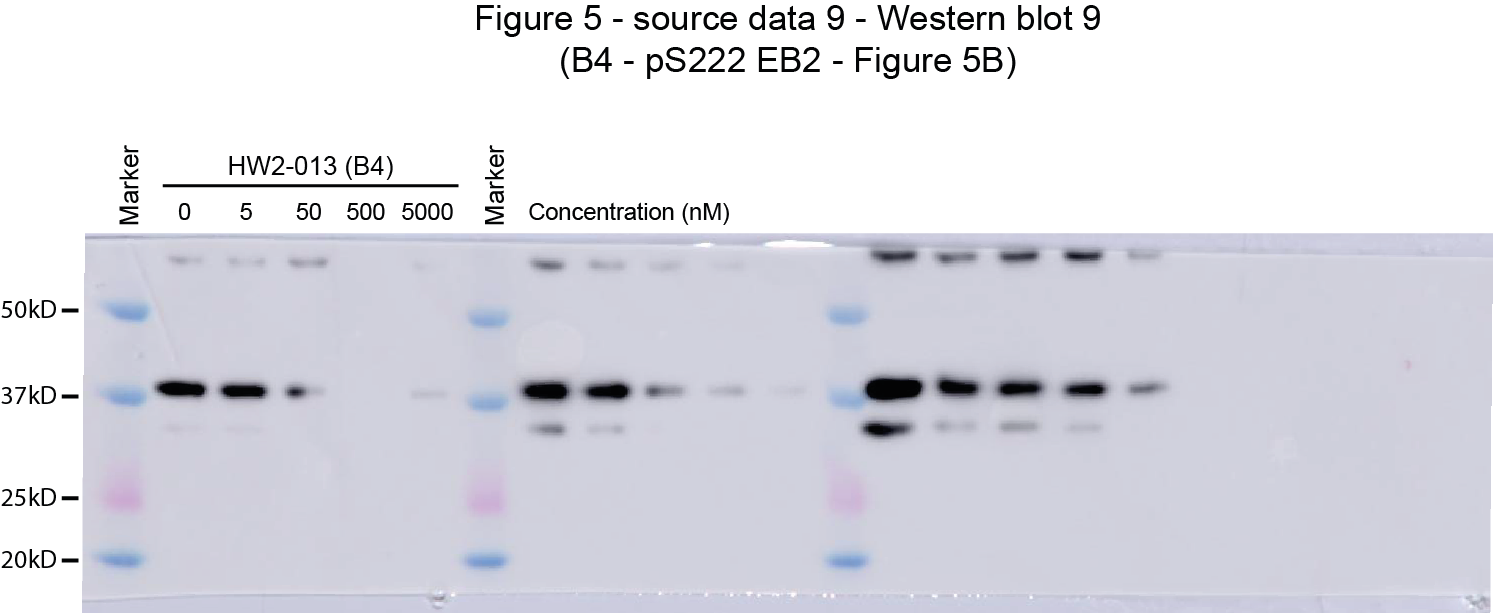

Supplement: Figure 5—source data 1. [file elife-88206-fig5-data1.zip › Figure 5 - source data/Figure 5 - source data 9/Western blot 9 - B4 - pEB2 - labeled.png]

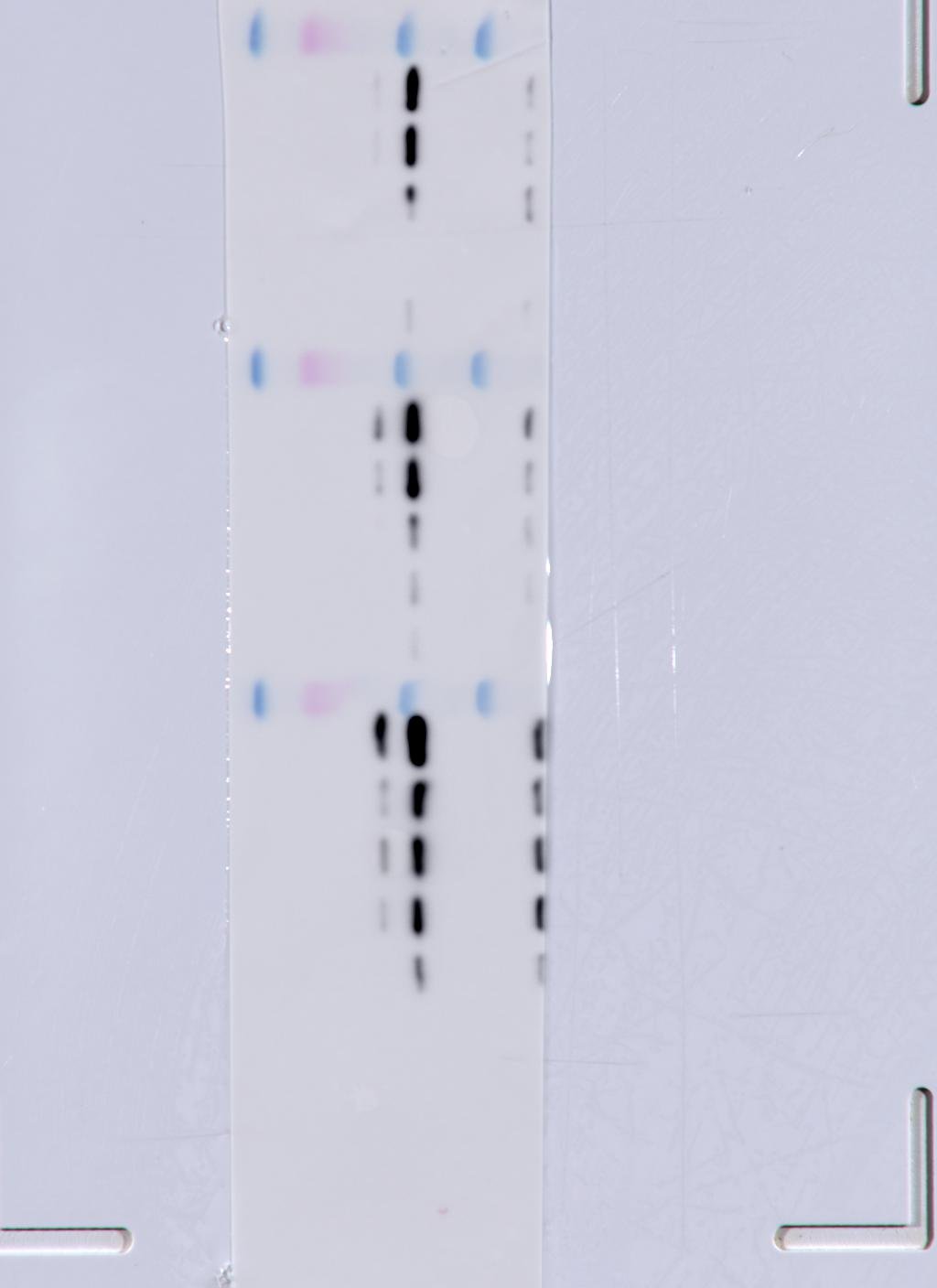

Supplement: Figure 5—source data 1. [file elife-88206-fig5-data1.zip › Figure 5 - source data/Figure 5 - source data 9/Western blot 9 - B4 - pEB2 - uncropped.jpg]

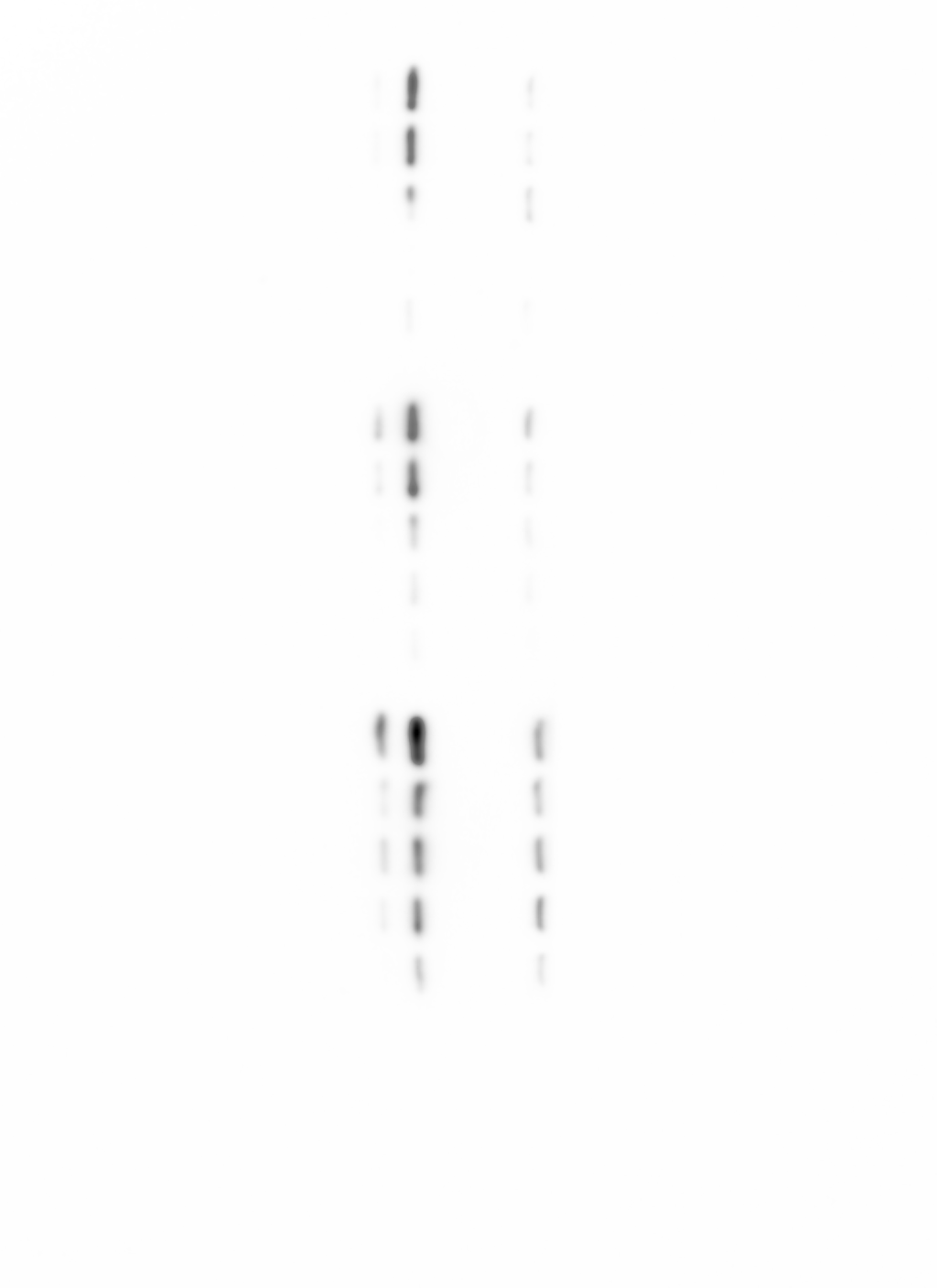

Supplement: Figure 5—source data 1. [file elife-88206-fig5-data1.zip › Figure 5 - source data/Figure 5 - source data 9/Western blot 9 - B4 - pEB2 - uncropped.tif]

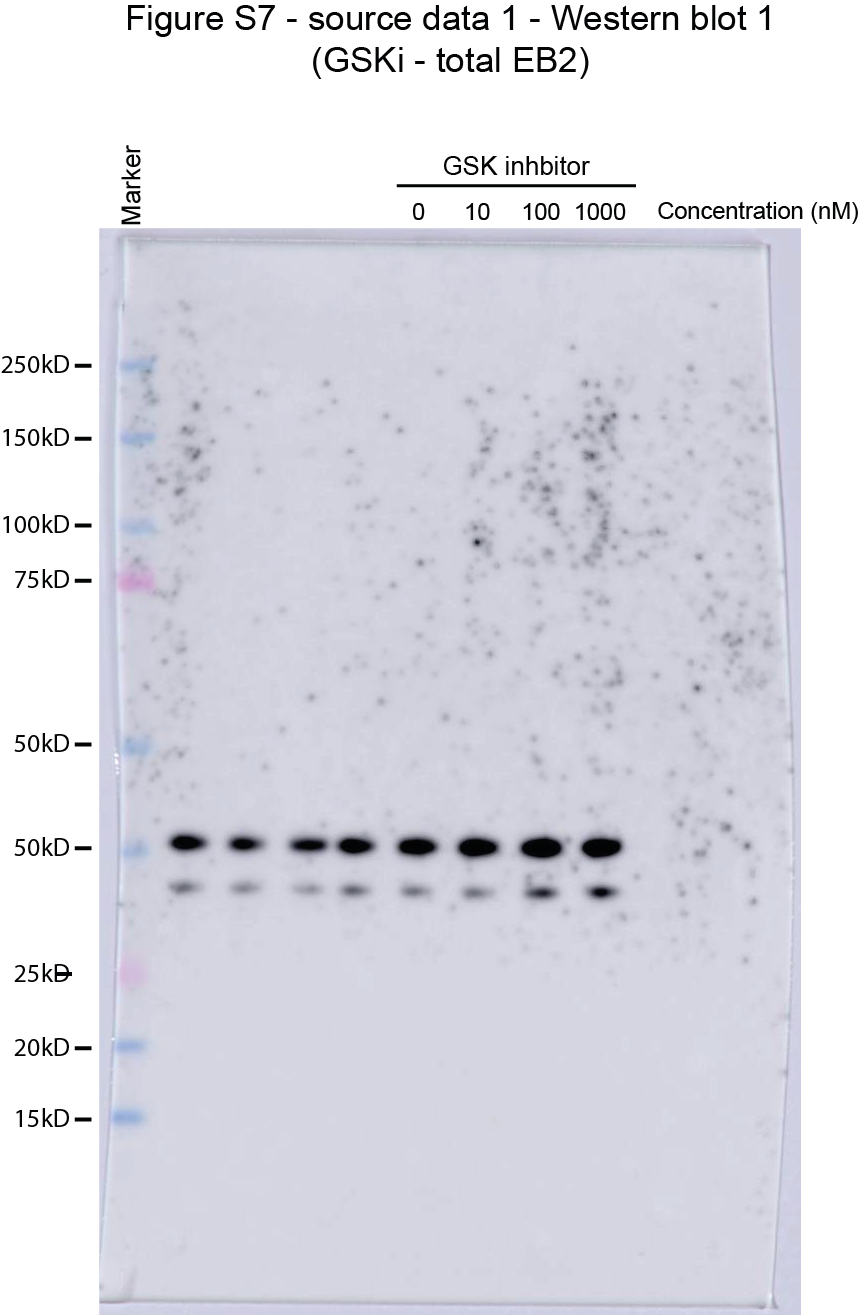

Supplement: Figure 5—figure supplement 1—source data 1. [file elife-88206-fig5-figsupp1-data1.zip › Figure S7 - source data/Figure S7 - source data 1/Western blot 1 - GSKi - EB2 - labeled.png]
