# Supplementary material for: Discovery and characterization of a specific inhibitor of serine-threonine kinase cyclin-dependent kinase-like 5 (CDKL5) demonstrates role in hippocampal CA1 physiology
Source: eLife. 2023 Jul 25;12:e88206. doi: 10.7554/eLife.88206 (PMC10406435; doi:10.7554/eLife.88206)
Supplement: Figure 4—source data 1. [file elife-88206-fig4-data1.zip › Figure 2 source 2.pdf]

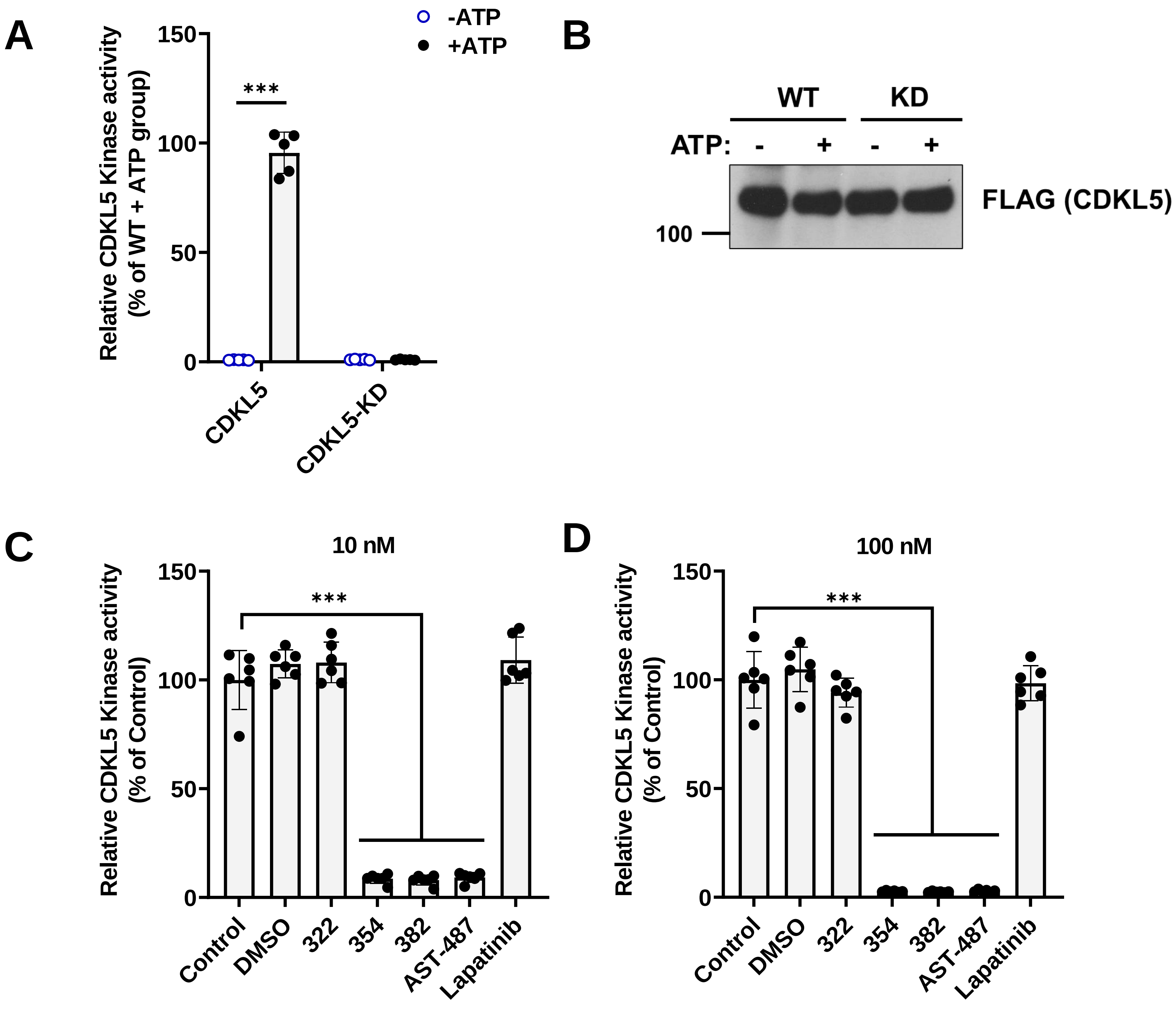

FLAG-tagged CDKL5 (human) WT or Kinase dead (KD) constructs were subcloned into pT7CFE1-CHis plasmid (Thermo Fisher). These constructs were then used for in vitro translation using a HeLa cell lysate-based Kit (1-Step Human Coupled IVT Kit—DNA, 88881, Life Technologies). The in vitro-translated proteins were then purified using His Pur cobalt spin columns (Thermo Scientific). For in vitro kinase assays, recombinant CDKL5 and myelin basic protein (Active Motif, 31314) as substrate were incubated in a kinase buffer (Cell Signaling, 9802) supplemented with or without adenosine 5′-triphosphate (ATP) at 30 °C for 30 minutes followed by kinase assays using ADP-Glo Kinase Assay kit (Promega). (**A**) Representative graph from a kinase assay showing that purified WT CDKL5 retains kinase activity, while the KD protein was functionally inactive. (**B**) Representative western blot showing equal levels of WT and KD proteins in the kinase assay experiments. (**C-D**) Purified WT CDKL5 was used for kinase assays as described above in the presence of indicated compounds at 10 or 100 nM concentrations. The results show that 354 and 382 can potently inhibit CDKL5.
These methods are described in the following reference: PMID: 32317630.

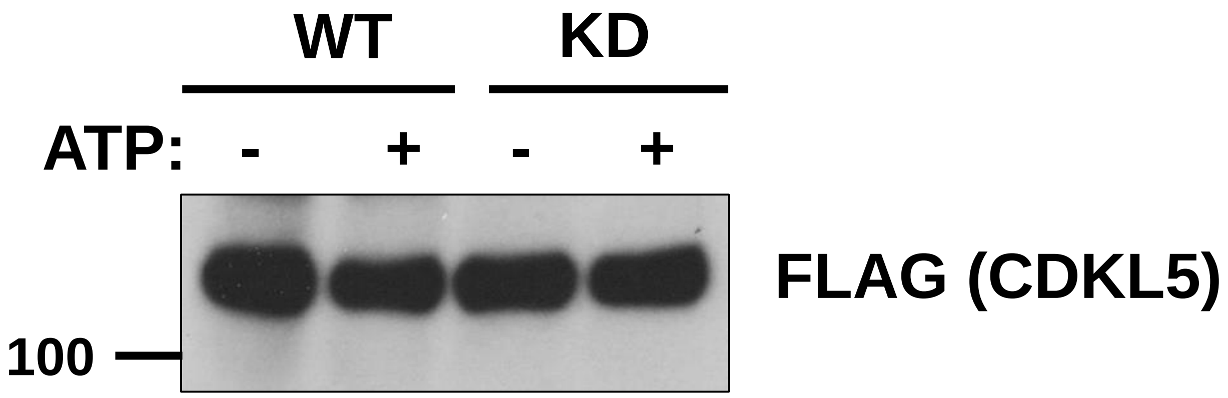

WT
KD
ATP: - + - +
FLAG (CDKL5)
100

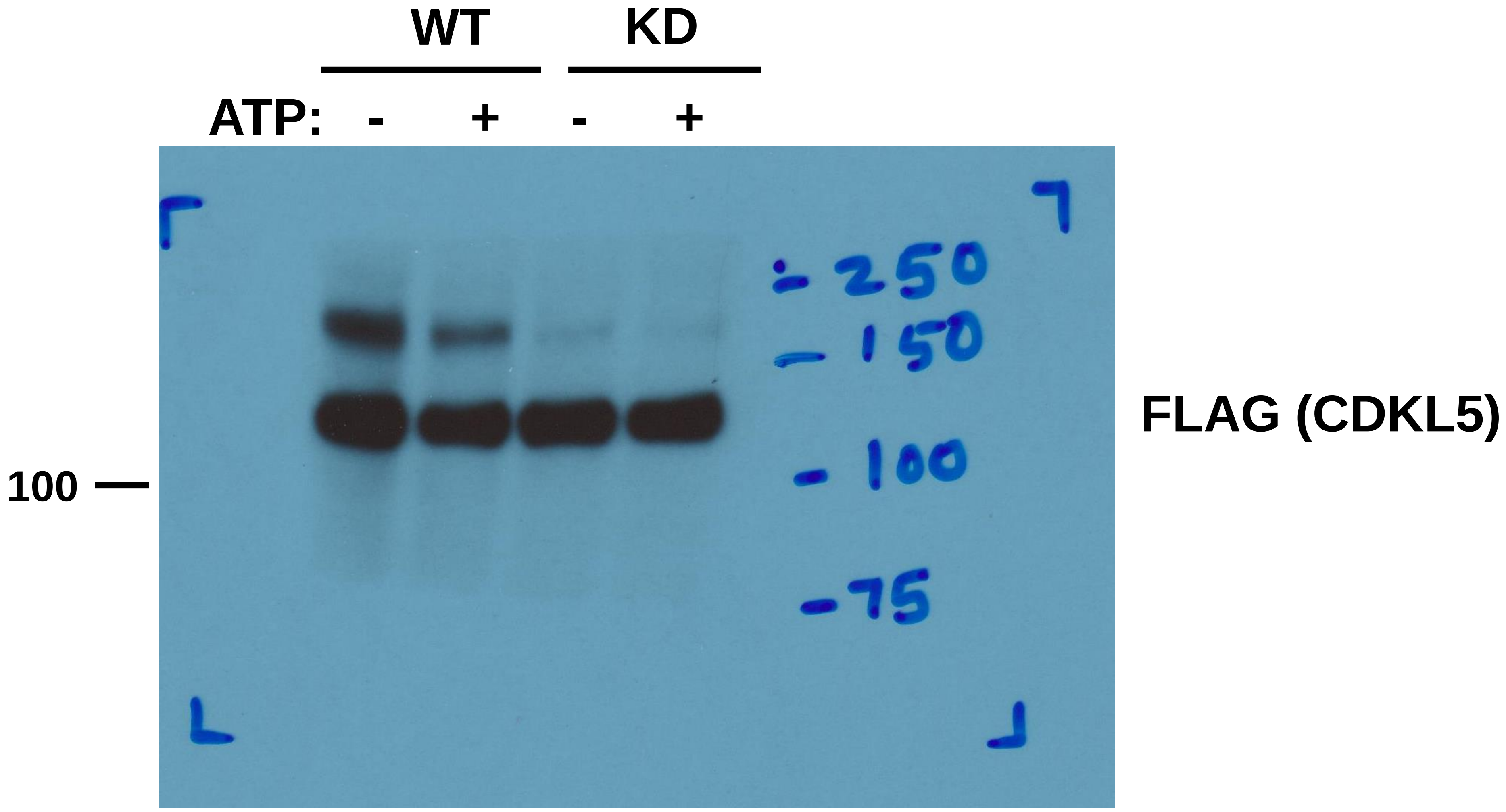

WT
KD
ATP: - + - +
FLAG (CDKL5)
100
250
150
100
75
