# Supplementary figures and images for: Discovery and characterization of a specific inhibitor of serine-threonine kinase cyclin-dependent kinase-like 5 (CDKL5) demonstrates role in hippocampal CA1 physiology (part 2 of 2)
Source: eLife. 2023 Jul 25;12:e88206. doi: 10.7554/eLife.88206 (PMC10406435; doi:10.7554/eLife.88206)

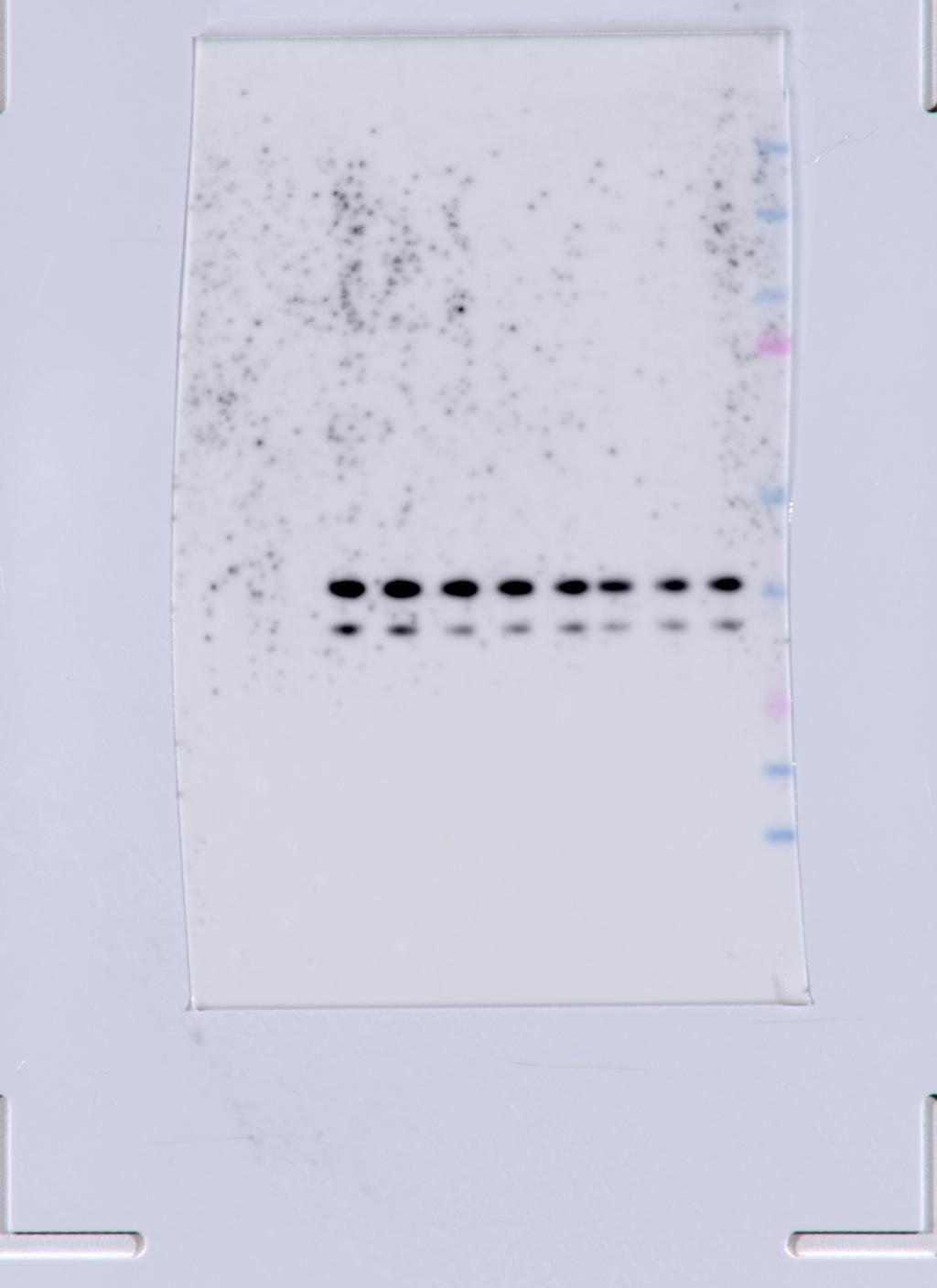

Supplement: Figure 5—figure supplement 1—source data 1. [file elife-88206-fig5-figsupp1-data1.zip › Figure S7 - source data/Figure S7 - source data 1/Western blot 1 - GSKi - EB2 - uncropped.jpg]

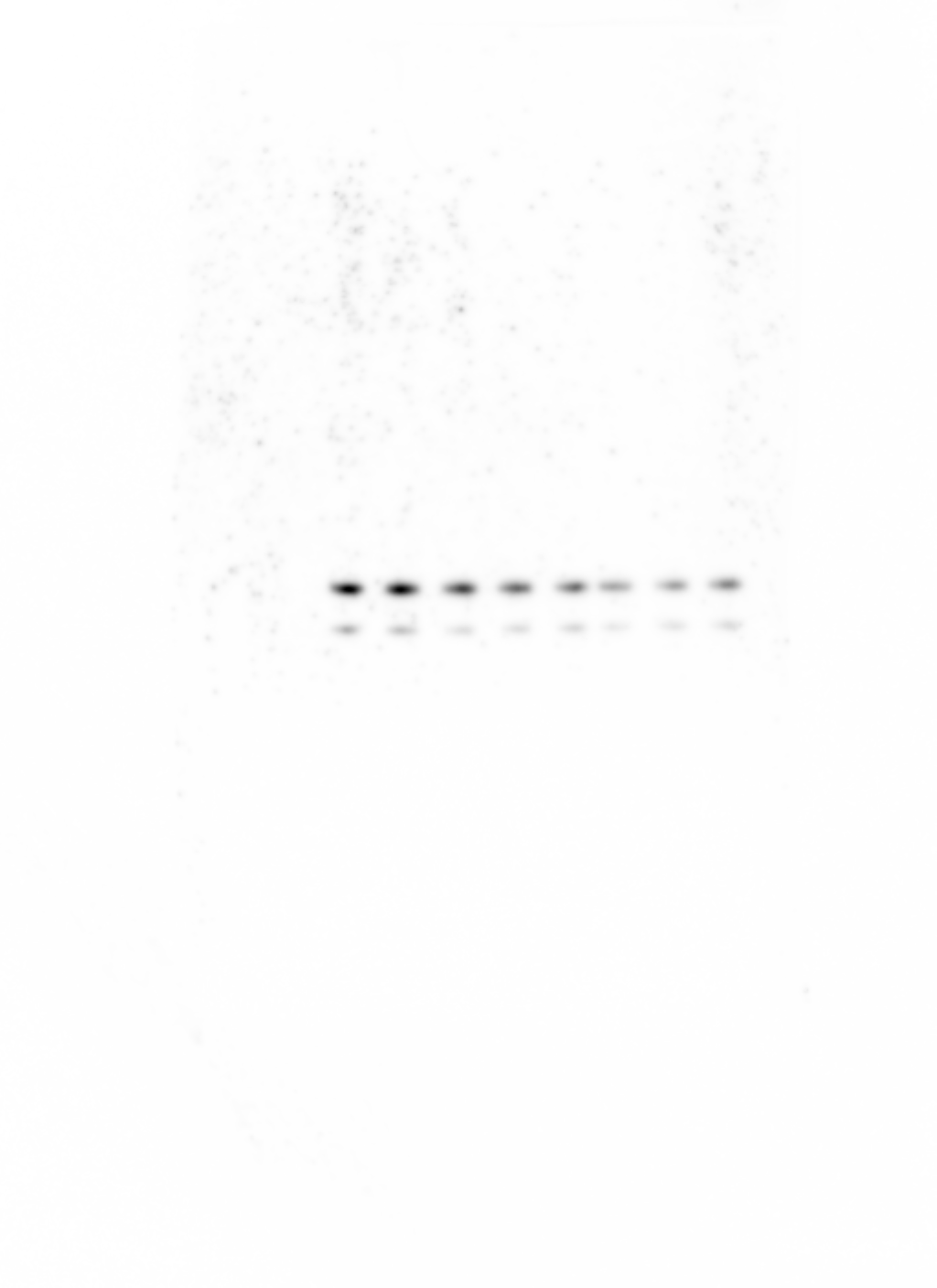

Supplement: Figure 5—figure supplement 1—source data 1. [file elife-88206-fig5-figsupp1-data1.zip › Figure S7 - source data/Figure S7 - source data 1/Western blot 1 - GSKi - EB2 - uncropped.tif]

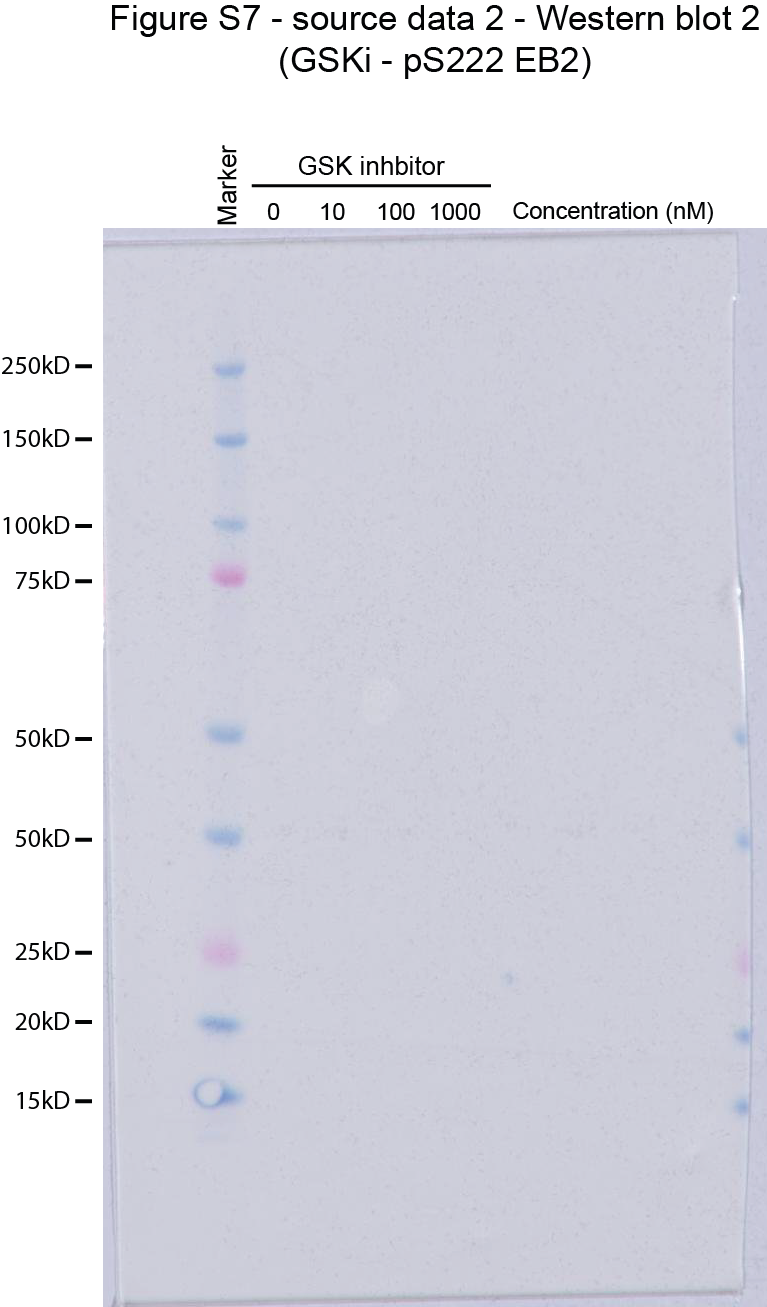

Supplement: Figure 5—figure supplement 1—source data 1. [file elife-88206-fig5-figsupp1-data1.zip › Figure S7 - source data/Figure S7 - source data 2/Western blot 3 - GSKi - pEB2 - labeled.png]

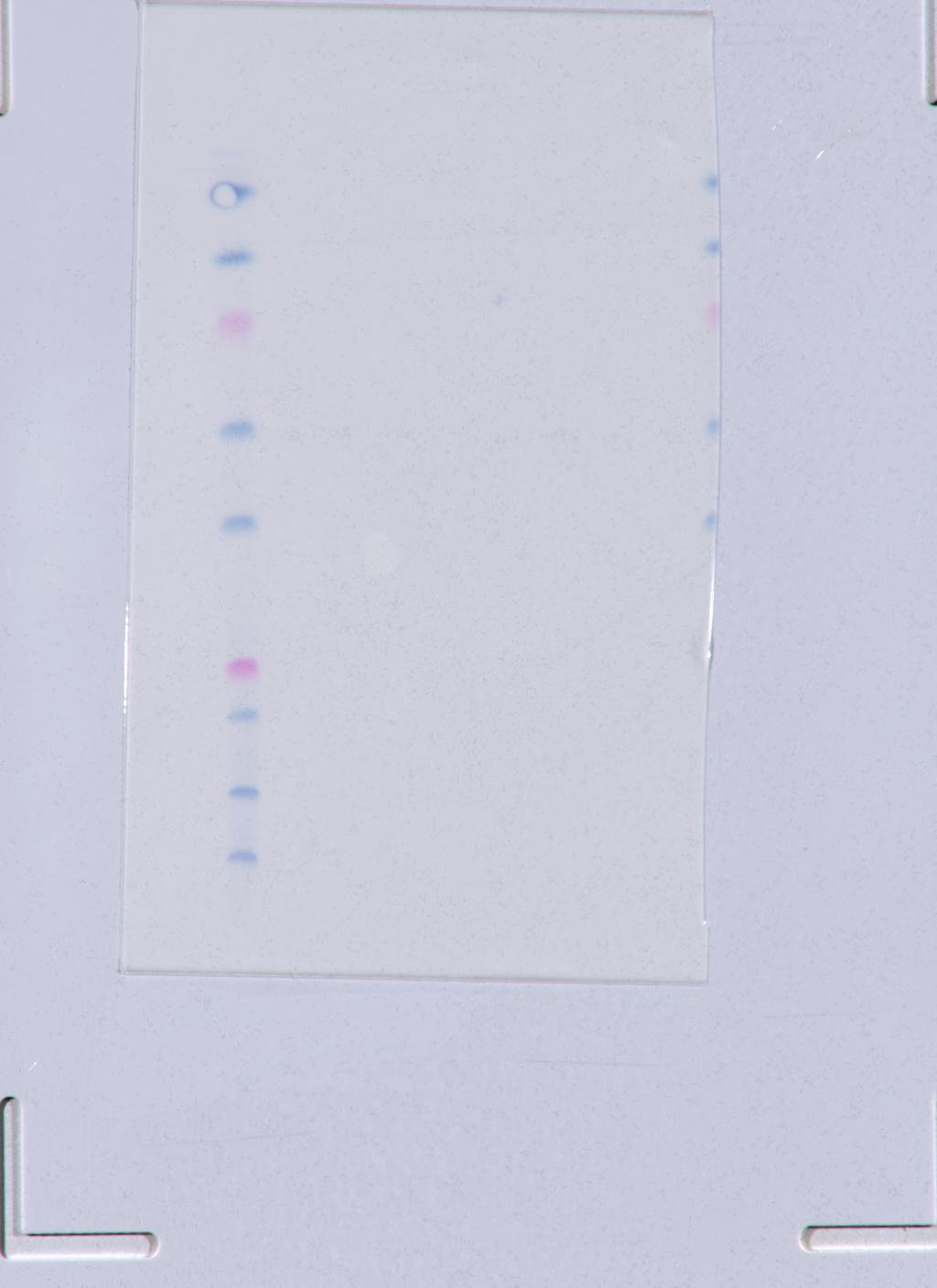

Supplement: Figure 5—figure supplement 1—source data 1. [file elife-88206-fig5-figsupp1-data1.zip › Figure S7 - source data/Figure S7 - source data 2/Western blot 3 - GSKi - pEB2 - uncropped.jpg]

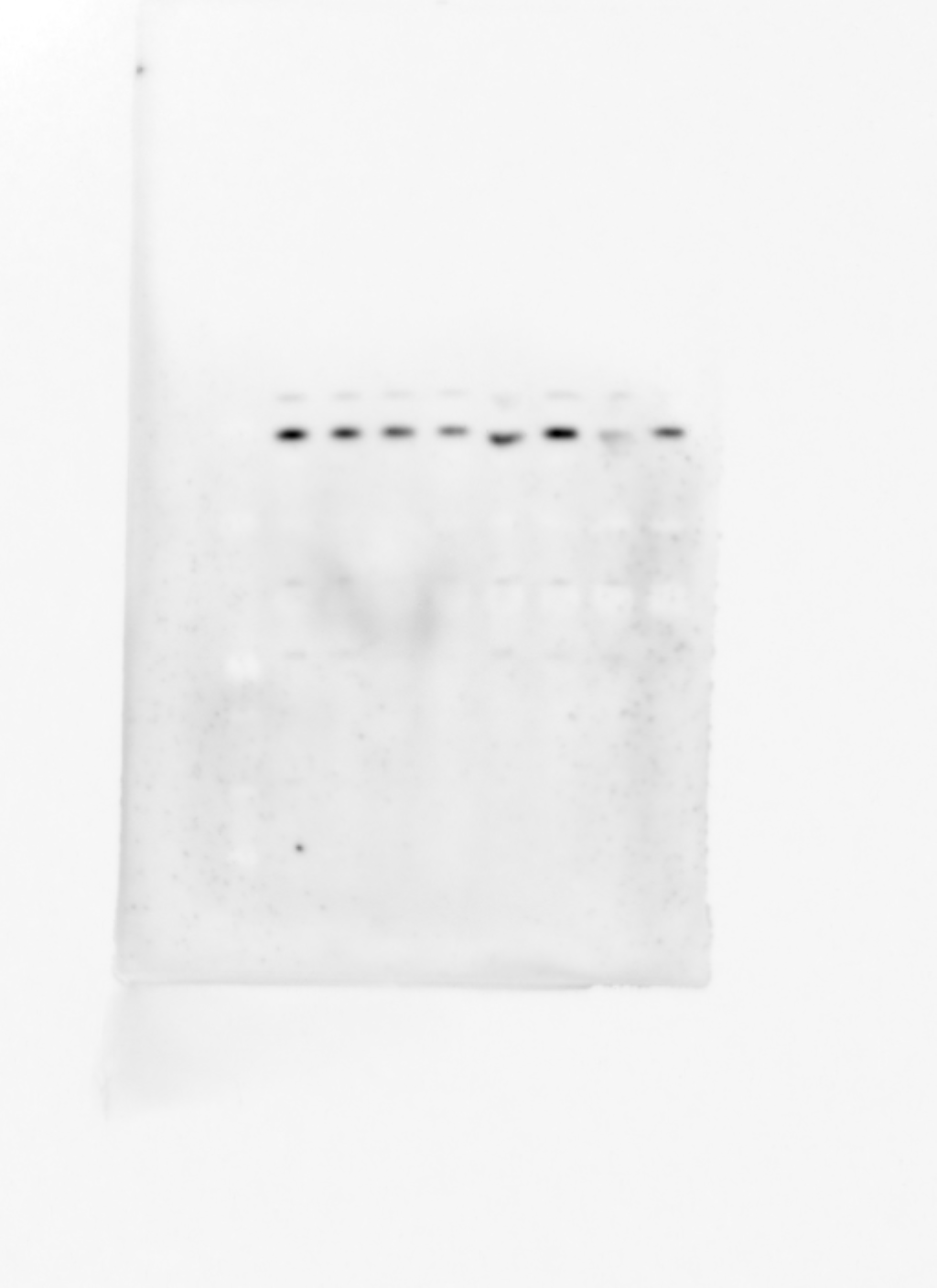

Supplement: Figure 5—figure supplement 1—source data 1. [file elife-88206-fig5-figsupp1-data1.zip › Figure S7 - source data/Figure S7 - source data 2/Western blot 3 - GSKi - pEB2 - uncropped.tif]

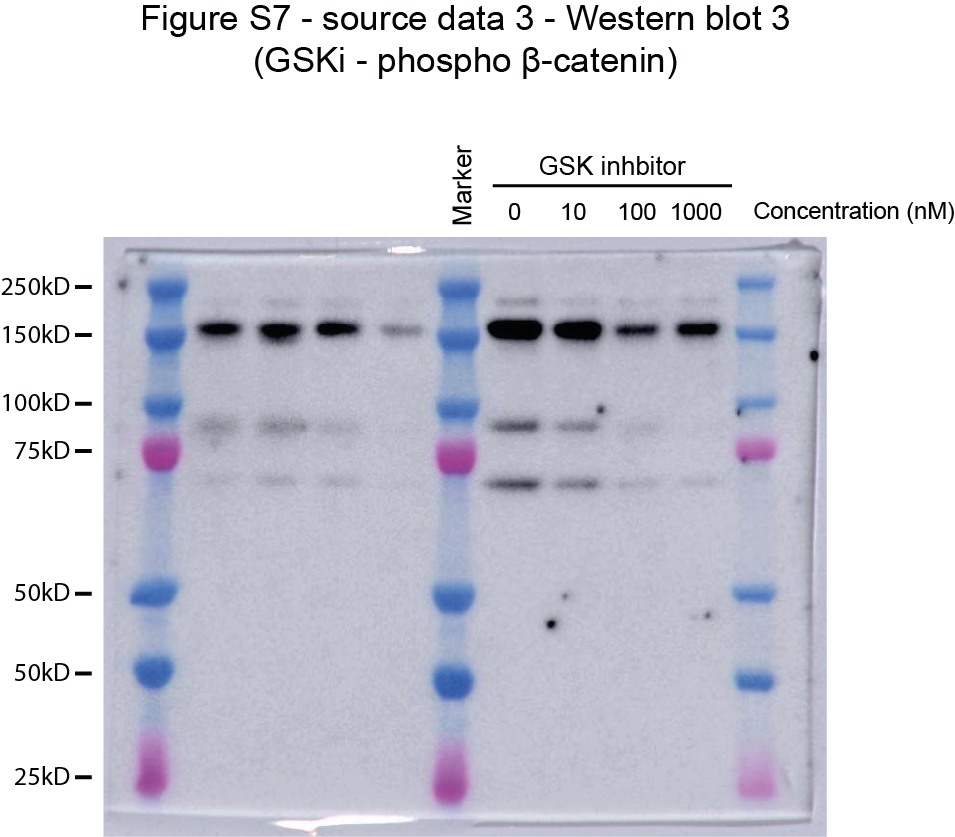

Supplement: Figure 5—figure supplement 1—source data 1. [file elife-88206-fig5-figsupp1-data1.zip › Figure S7 - source data/Figure S7 - source data 3/Western blot 3 - GSKi - phospho bcatenin - labeled.png]

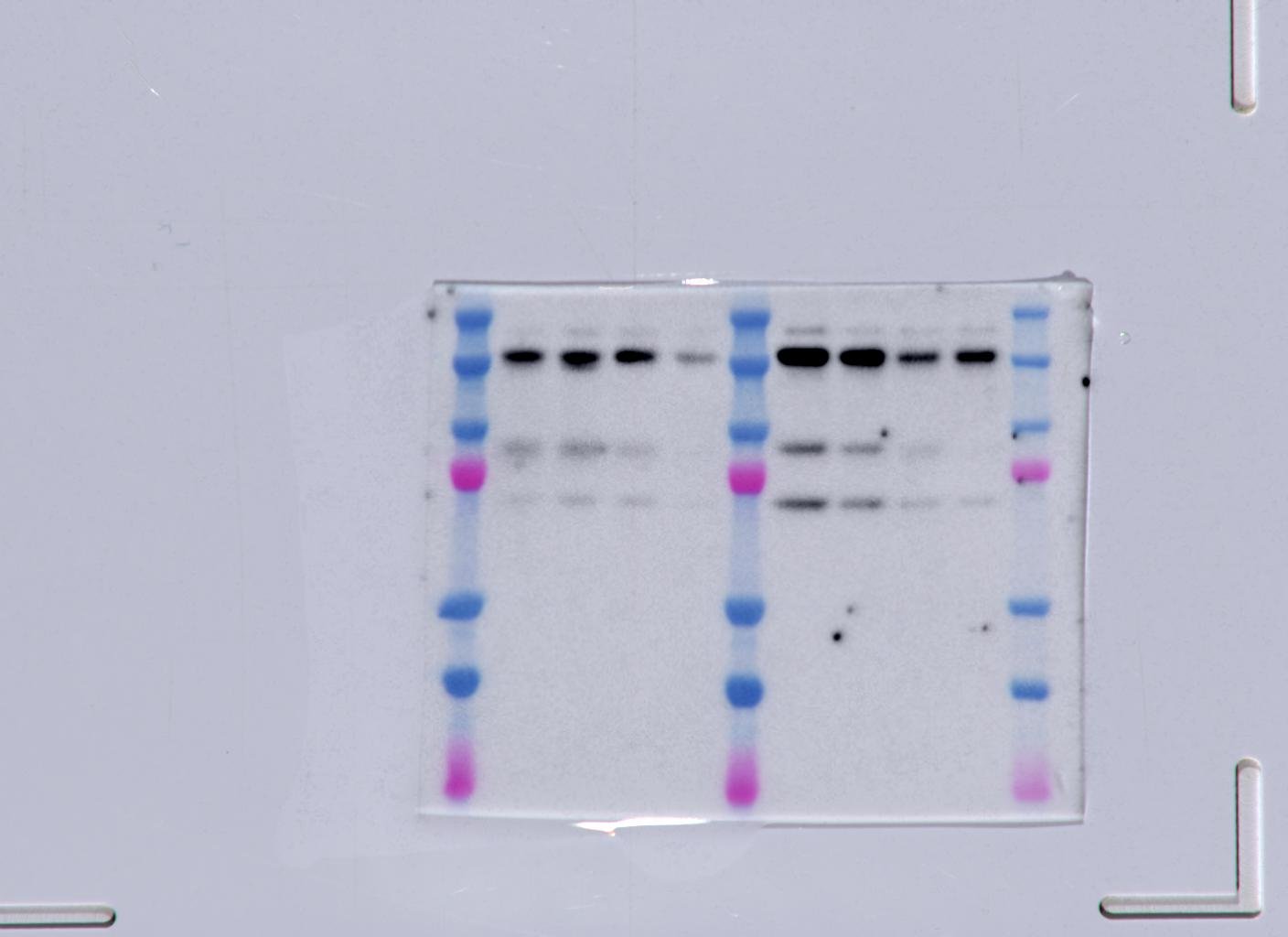

Supplement: Figure 5—figure supplement 1—source data 1. [file elife-88206-fig5-figsupp1-data1.zip › Figure S7 - source data/Figure S7 - source data 3/Western blot 3 - GSKi - phospho bcatenin - uncropped.jpg]

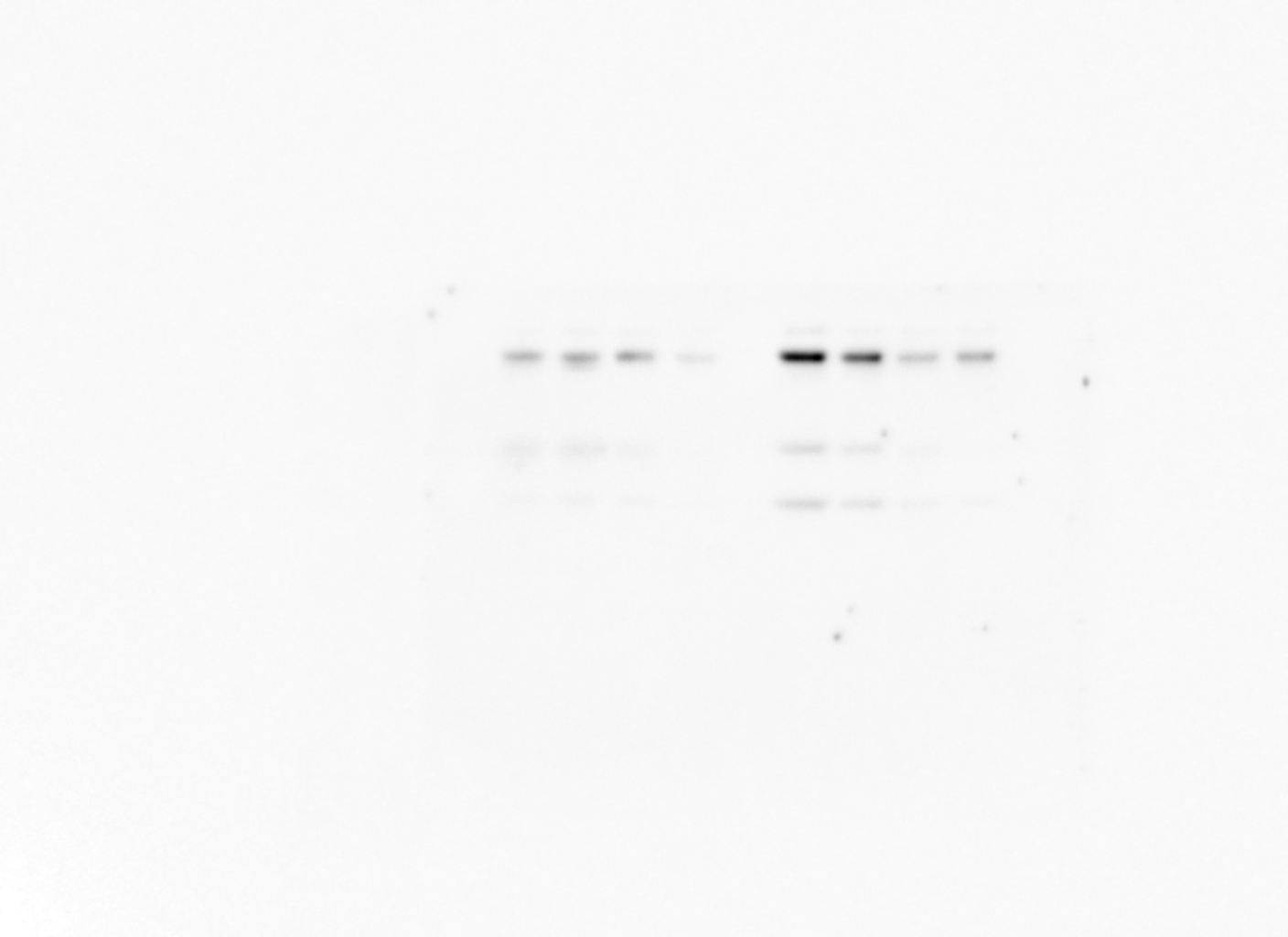

Supplement: Figure 5—figure supplement 1—source data 1. [file elife-88206-fig5-figsupp1-data1.zip › Figure S7 - source data/Figure S7 - source data 3/Western blot 3 - GSKi - phospho bcatenin - uncropped.tif]

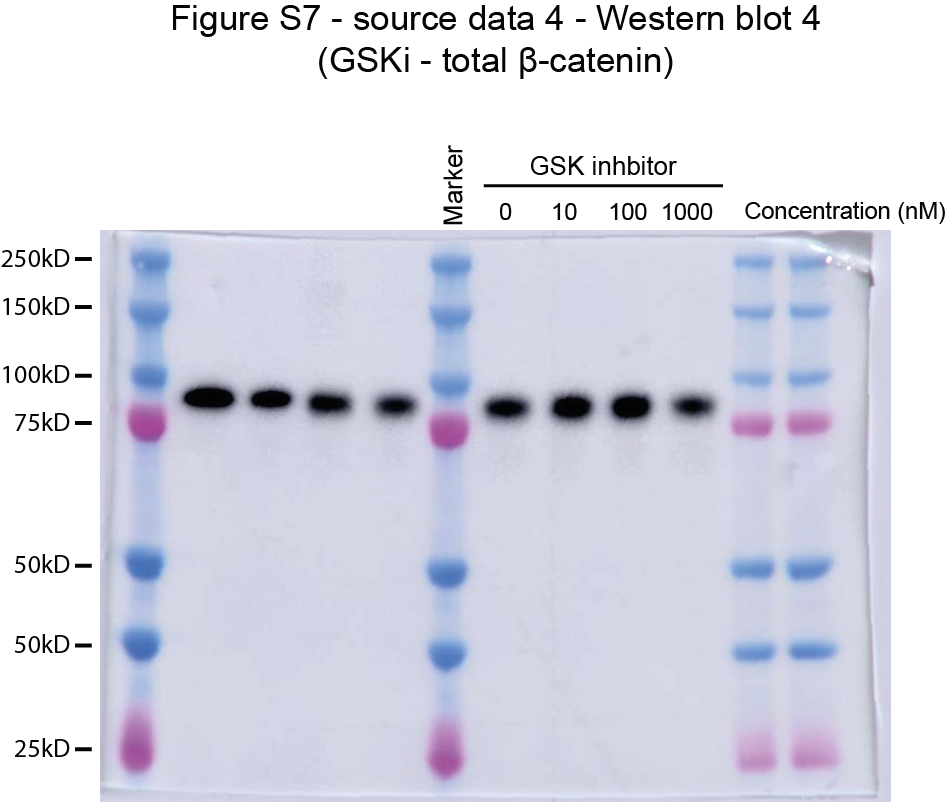

Supplement: Figure 5—figure supplement 1—source data 1. [file elife-88206-fig5-figsupp1-data1.zip › Figure S7 - source data/Figure S7 - source data 4/Western blot 4 - GSKi - total bcatenin - labeled.png]

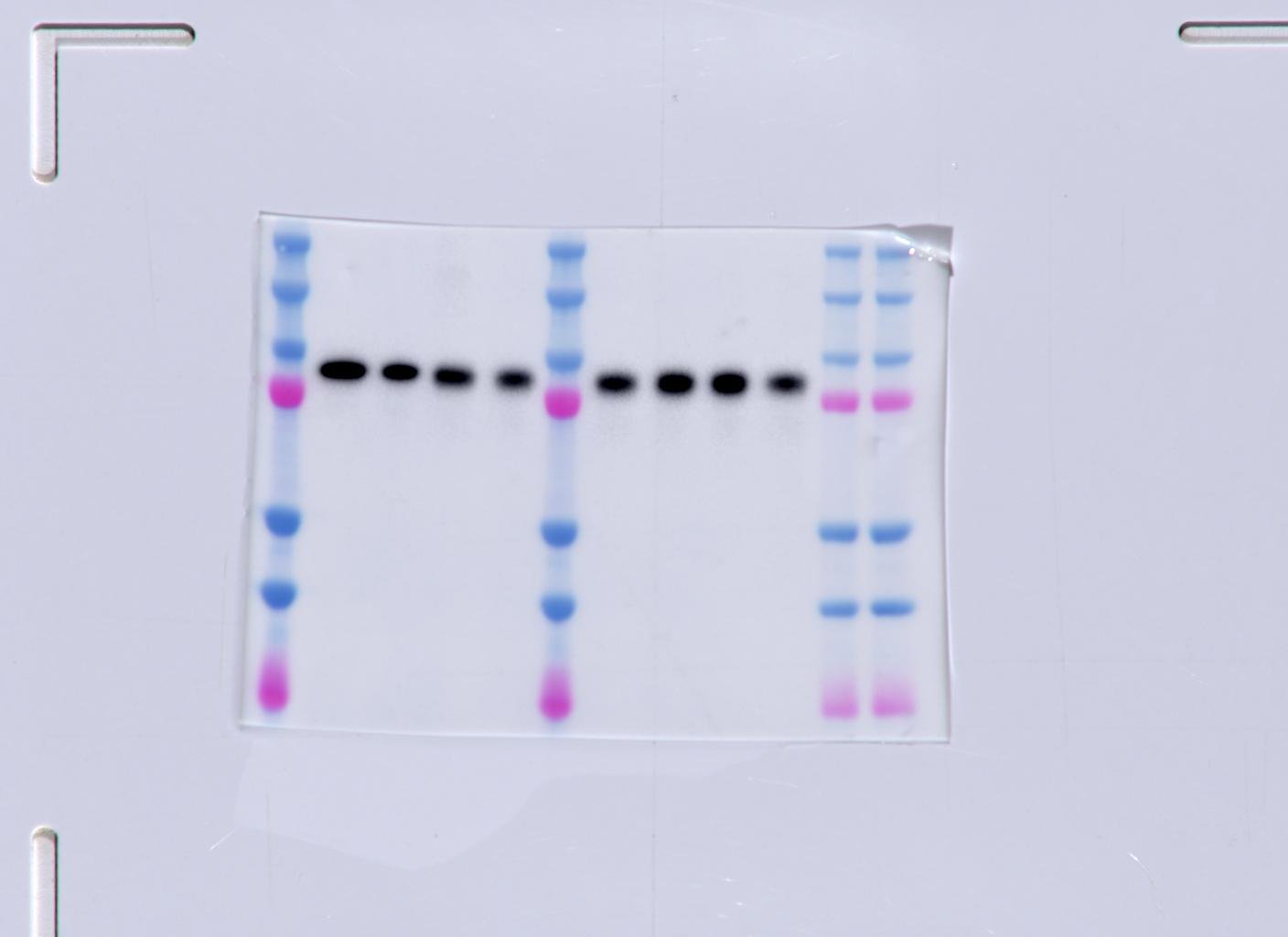

Supplement: Figure 5—figure supplement 1—source data 1. [file elife-88206-fig5-figsupp1-data1.zip › Figure S7 - source data/Figure S7 - source data 4/Western blot 4 - GSKi - total bcatenin - uncropped.jpg]

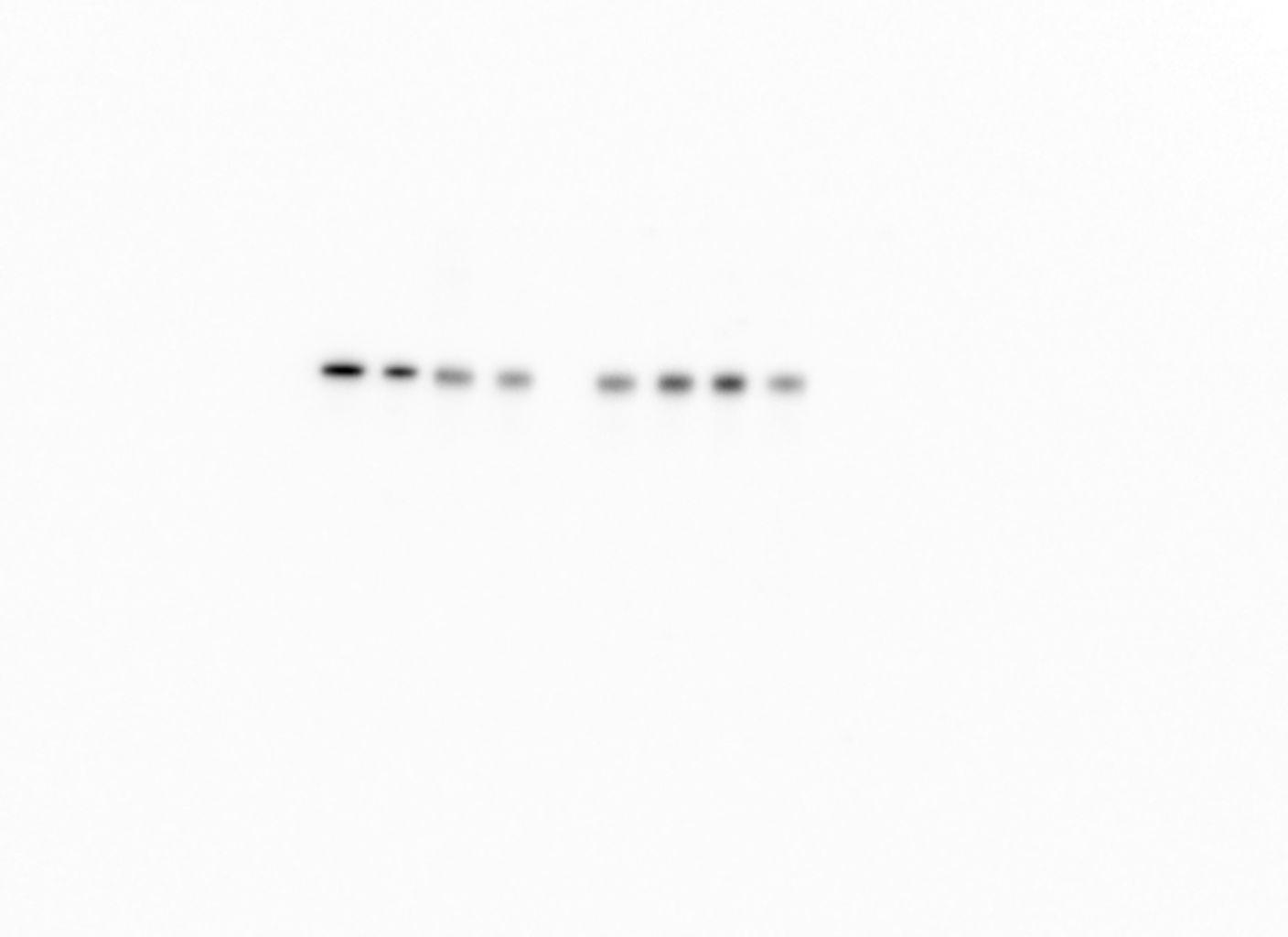

Supplement: Figure 5—figure supplement 1—source data 1. [file elife-88206-fig5-figsupp1-data1.zip › Figure S7 - source data/Figure S7 - source data 4/Western blot 4 - GSKi - total bcatenin - uncropped.tif]

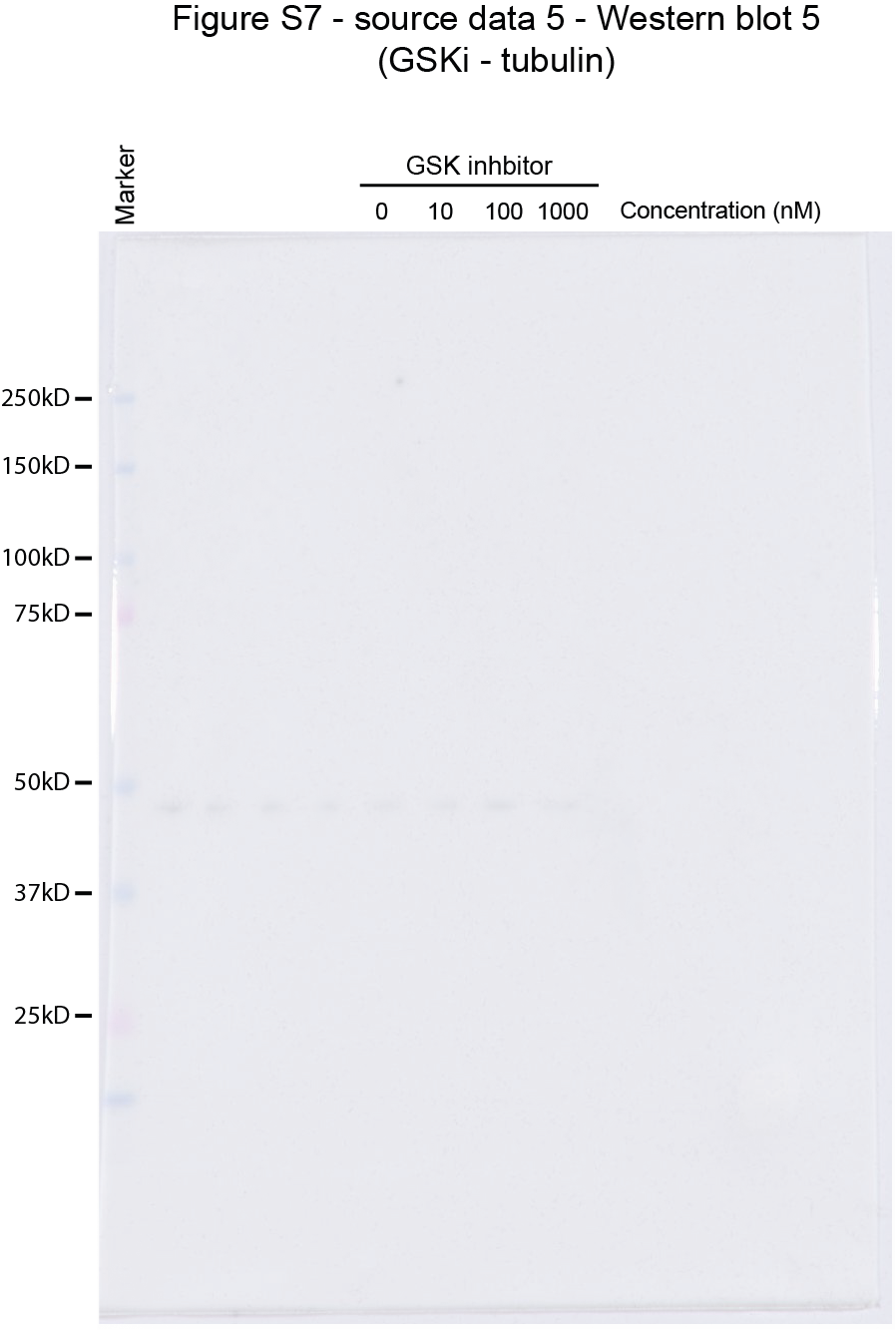

Supplement: Figure 5—figure supplement 1—source data 1. [file elife-88206-fig5-figsupp1-data1.zip › Figure S7 - source data/Figure S7 - source data 5/Westerm blot 5 - GSKi - tubulin - labeled.png]

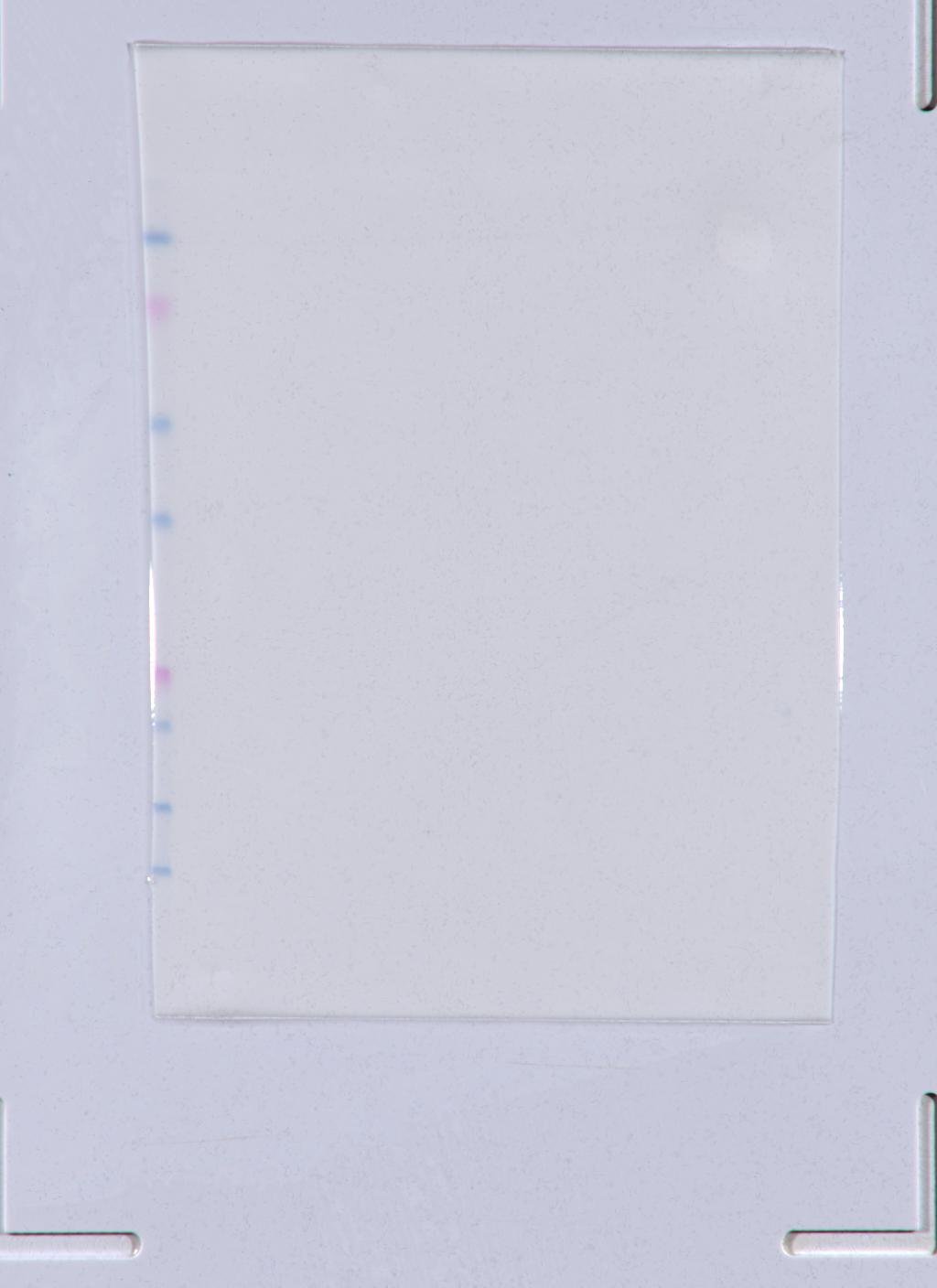

Supplement: Figure 5—figure supplement 1—source data 1. [file elife-88206-fig5-figsupp1-data1.zip › Figure S7 - source data/Figure S7 - source data 5/Westerm blot 5 - GSKi - tubulin - uncropped.jpg]

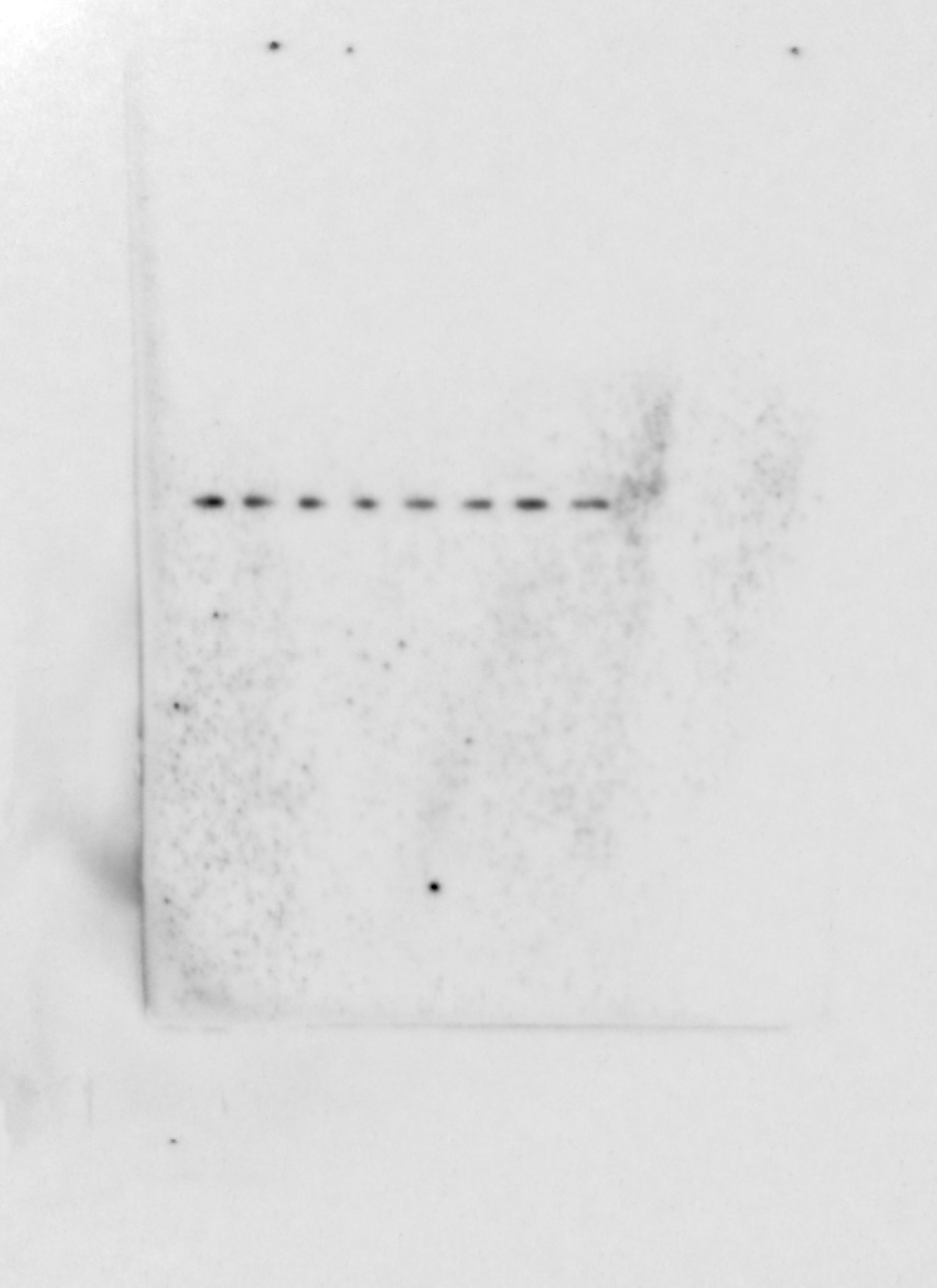

Supplement: Figure 5—figure supplement 1—source data 1. [file elife-88206-fig5-figsupp1-data1.zip › Figure S7 - source data/Figure S7 - source data 5/Westerm blot 5 - GSKi - tubulin - uncropped.tif]

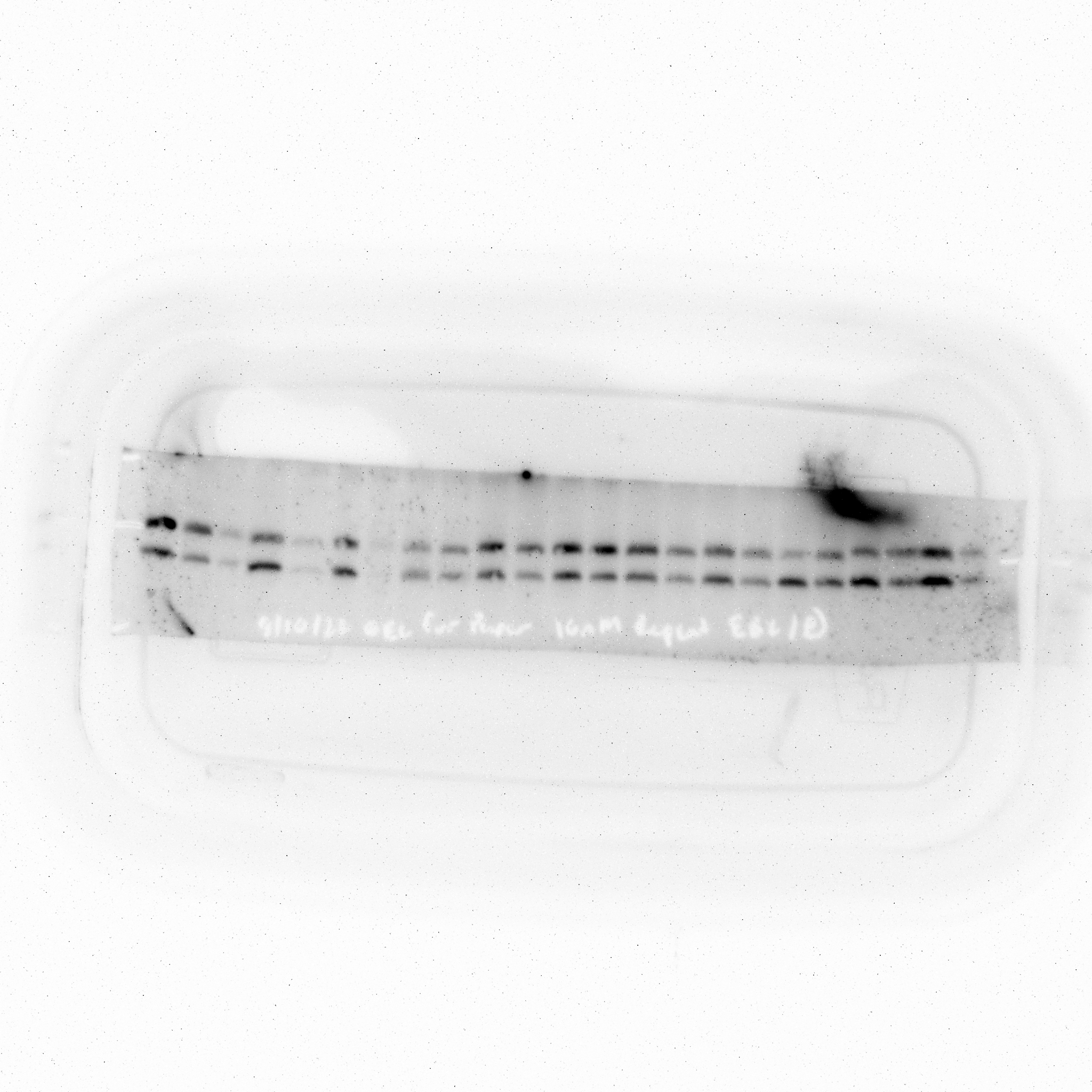

Supplement: Figure 6—source data 1. [file elife-88206-fig6-data1.zip › 081122 eb2P blotfor paper and 10nM repeat full no label inverted.tif]

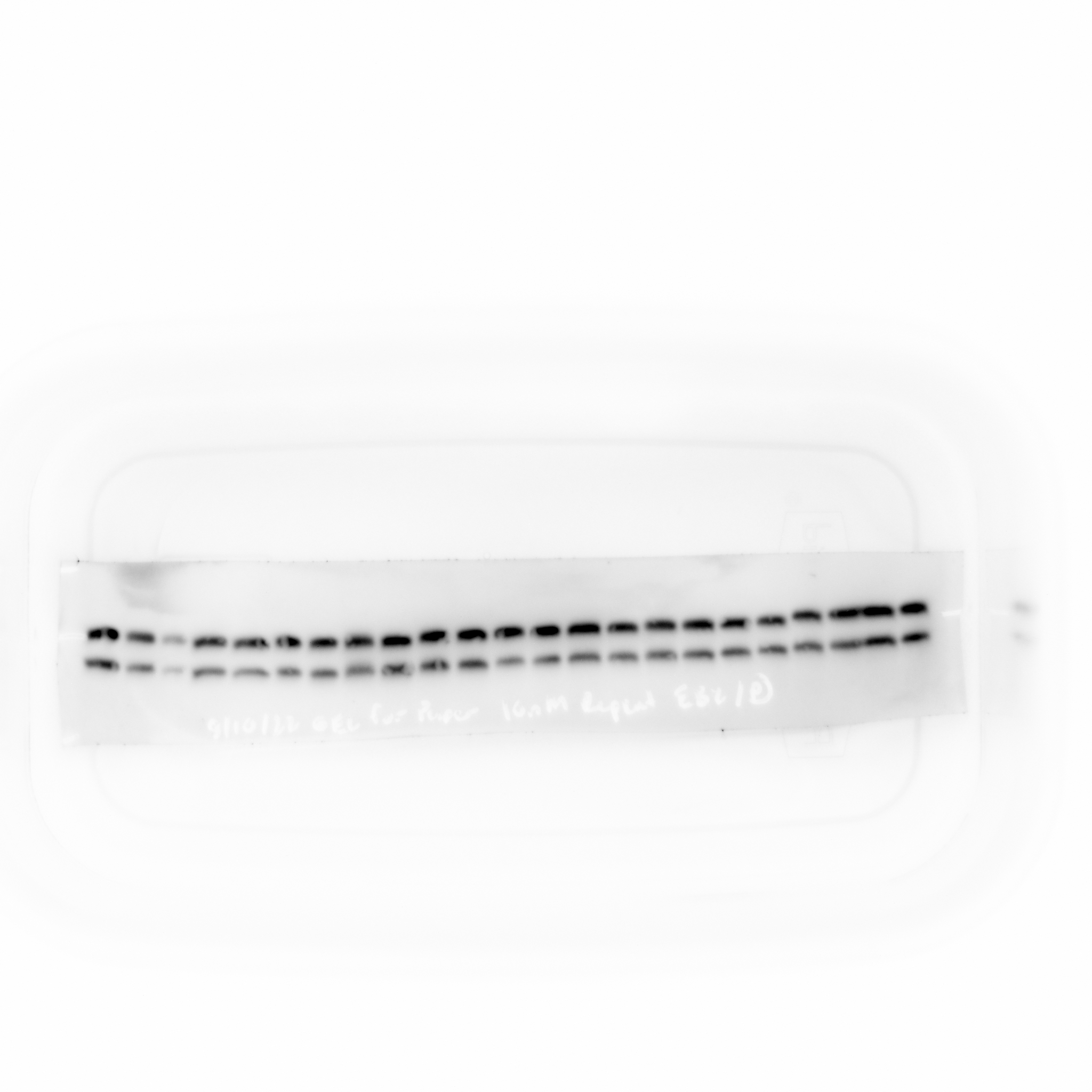

Supplement: Figure 6—source data 2. [file elife-88206-fig6-data2.zip › 081122 eb2noP blotfor paper and 10nM repeat full blot no label inverted.tif]

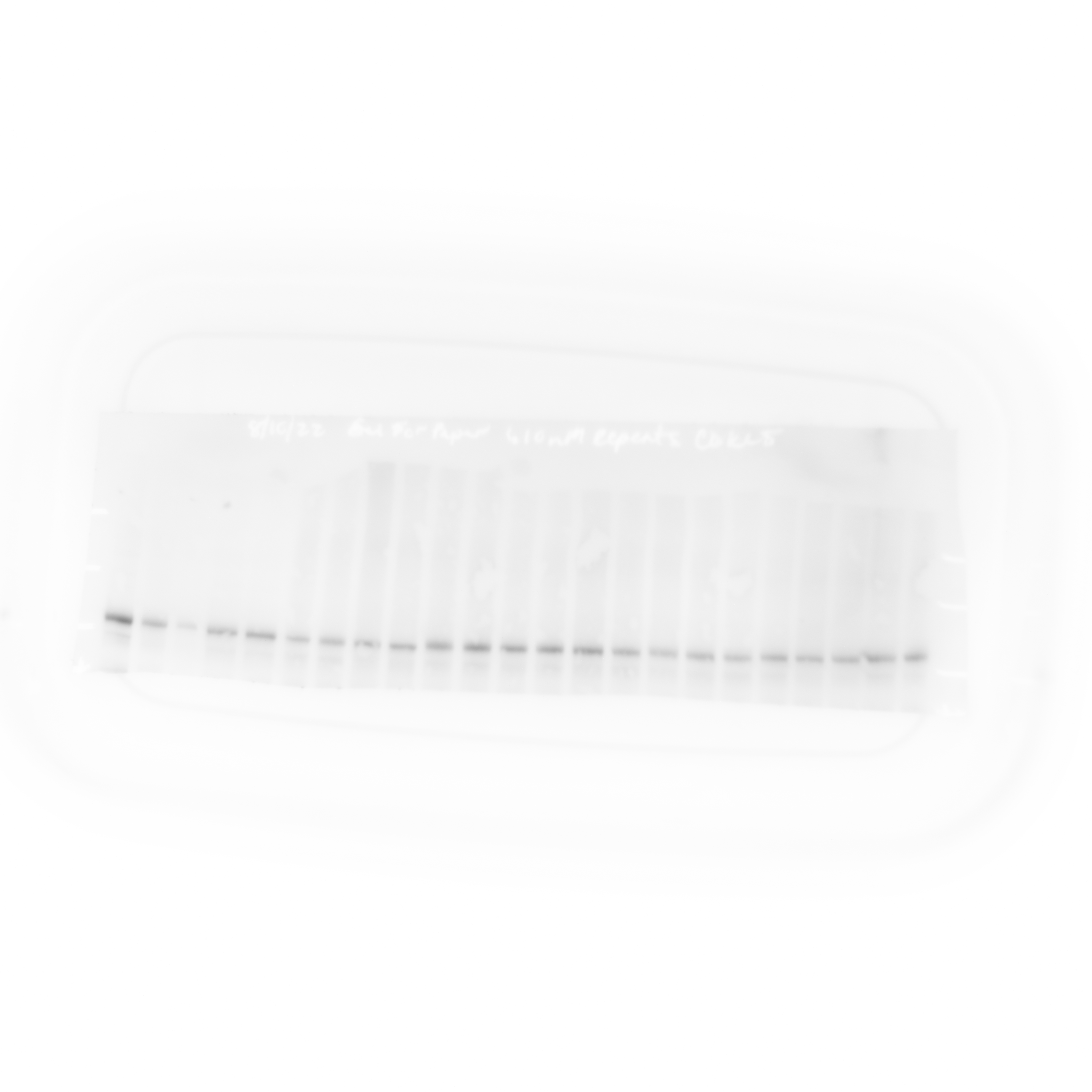

Supplement: Figure 6—source data 3. [file elife-88206-fig6-data3.zip › 081122 CDKL5 blotfor paper and 10nM no label inverted.tif]
